# Promising benefits of six-month *Phaeodactylum tricornutum* microalgae supplementation on cognitive function and inflammation in healthy older adults with age-associated memory impairment

**DOI:** 10.3389/fragi.2025.1540115

**Published:** 2025-04-30

**Authors:** Emily Goodbody, Jonathan Maury, Andrea Doolan, Gillian DunnGalvin, Charles Kakilla, Rémi Pradelles, Timothy G. Dinan

**Affiliations:** ^1^ Atlantia Food Clinical Trials, Heron House Offices, Blackpool, Ireland; ^2^ Microphyt, Research and Development Department, Baillargues, France; ^3^ APC Microbiome Ireland, University College Cork, Cork, Ireland; ^4^ Department of Psychiatry and Neurobehavioural Science, University College Cork, Cork, Ireland

**Keywords:** healthy aging, dietary supplement, microalgae, cognitive impairment, memory, neuroprotection, fucoxanthin

## Abstract

**Introduction:**

Aging is often associated with cognitive decline and memory impairment, with several factors including inflammatory cytokines and oxidative stress markers implicated in this natural process. Microalgae extracts are a natural source of many bioactive compounds that reduce inflammation and oxidative stress, and represent an innovative dietary approach to ameliorate age-related cognitive decline and memory impairment. This proof-of-concept CONSORT-compliant, double-blind, randomized controlled study was conducted to evaluate the effect of daily supplementation of microalgae extract on cognitive function, mood, stress and inflammation of healthy older adults with mild cognitive impairment over a 24-week period.

**Methods:**

Sixty-six volunteers with age-associated memory impairment (AAMI; age 55–75 years) were randomly assigned to ingest a placebo (PL, maltodextrin) or 550 mg of *Phaeodactylum tricornutum* (Pt) extract (4.4 mg of fucoxanthin, PUFAs and saturated fatty acids). Participants performed the COMPASS cognitive test battery to measure spatial, working, and episodic memory, attention, vigilance, and executive function. Sleep quality, mood, stress states and inflammation markers were also evaluated. All endpoints were collected at baseline, weeks 12 and 24.

**Results:**

There were no between-group differences for the primary outcome (Corsi Block Span Score) or other cognitive function parameters, at either 12 week or 24 weeks. However, within groups, 24 weeks of daily intake of microalgae extract derived from Phaeodactylum tricornutum attenuated age-induced readouts in Stroop task overall reaction time (p = 0.005; Cohen’s d = 0.8) and Word recall delayed score (p = 0.010; Cohen’s d = 0.5) as compared to baseline week 0, while no significance was reported for these readouts in the placebo group. Similar findings were reported for participants’ perceived stress (p = -0.04; Cohen’s d = 0.4). There was a significant decrease in blood hs-CRP from 3.9mg/L to 2.1mg/L following 24-weeks of Pt extract supplementation as compared to placebo (p = 0.002; Cohen’s d = 0.8), while there was no adverse impact on safety clinical blood tests or reported side effects, with the product deemed safe and tolerable.

**Discussion:**

The findings suggest promising benefits of daily intake of microalgae extract on cognitive function and immune markers in older subjects with AAMI. Future research into the preventive role of Pt extract in age-assocaited cognitive decline is warranted.

**Clinical Trial Registration:**

Identifier #NCT04832412.

## 1 Introduction

Age-related cognitive decline refers to a typical, non-pathological reduction in cognitive abilities, such as processing speed, attention span, and both short-term (working) and long-term memory (Salthouse, 2009). These declines in cognitive processes result from normal and complex physiological, psychological and social interaction changes directly correlated with aging (Saadeh et al., 2020; Galkin et al., 2022; Gellert and Alonso-Perez, 2024), and it is estimated that brain disorders may surpass the combined impact of cardiovascular diseases and cancers in the coming years (Feigin et al., 2020; Lei and Gillespie, 2024). Although the exact physiological mechanisms behind age-related cognitive decline remain somewhat unclear, it is believed that oxidative stress and inflammatory pathways play a significant role in the etiology and progression of this phenomenon (Tönnies and Trushina, 2017; Scarmeas et al., 2018). Oxidative stress occurs when there is an imbalance between the production of reactive oxygen species (molecules such as free radicals that can cause damage to all cell types) and the body’s natural antioxidant defense system, and as we age the levels of natural antioxidants like glutathione, superoxide dismutase and catalase decline (Liguori et al., 2018). Further events that contribute to this imbalance as we age include an accumulation of damaged cells, and mitochondrial dysfunction leading to further ROS due to a less efficient mitochondrial cleansing process (Liguori et al., 2018). Oxidative stress, in turn, can contribute to inflammation in the brain. Evidence suggests that in the ageing brain, the microglial macrophages may become chronically activated, leading to the prolonged production of pro-inflammatory cytokines (e.g. IL-6; TNF-α) and proteins such as C-reactive protein (CRP), which are well-established predictors of cognitive decline (McGrattan et al., 2019; Lewis and Knight, 2021). A perturbation to the balance of pro- and anti-inflammatory cytokines can initiate a cycle of neuroinflammation, resulting in neuronal death, reduced brain volume, and cortical thinning (Gu et al., 2017).

Beyond pharmacological and behavioral preventive approaches, some studies have looked at the effects of specific dietary supplements on cognitive function during aging, including participants with age-associated memory impairment (AAMI) (Valls-Pedret et al., 2015; McGrattan et al., 2019). Specifically, the molecules from natural plant extracts, including those from spearmint, citicoline, bacopa, Ginkgo biloba or Astaxanthin, scientific data showed clinical benefits on cognitive function parameters, including memory, in aging populations (Herrlinger et al., 2018; Liu et al., 2019; McPhee et al., 2021; Queen et al., 2024). For example, Herrlinger et al. showed, in a double-blind, placebo-controlled trial, that a supplementation of 900 mg/day of spearmint (extract from Mentha spicata L.) for 90 days increased the working and spatial working memory accuracy in individuals with AAMI by 15% (Herrlinger et al., 2018). Another class of dietary supplement often associated with cognitive support in ageing are Ω-3 polyunsaturated fatty acids (LC-PUFAs) (Salem et al., 2015; Dighriri et al., 2022), which must be obtained from the diet that are considered necessary to support cell membrane integrity, neuronal development and maintenance, and inflammatory tone regulation.

Microalgae are living microscopic algae that are invisible to the naked eye that can produce an array of bioactive molecules (such as long-chain omega-3 fatty acids, especially EPA and docosahexaenoic acid (DHA), pigments, peptides, and sterols) that can be harvested using ecological and sustainable production methods to meet major societal challenges (Matos et al., 2017; Chen et al., 2022; Çelekli et al., 2024). A prominent example would be astaxanthin, a carotenoid well known for its anti-oxidative capacity (Satoh et al., 2009; Nakagawa et al., 2011; Katagiri et al., 2012; Ito et al., 2018). Indeed, a recent study showed the positive impact of 6 mg/day of astaxanthin (from *Haematococcus pluvialis* extract) supplementation taken for 12 weeks on cognitive parameters (psychomotor and processing speed) in participants with cognitive impairment (Aziz et al., 2020). Fucoxanthin, another prominent carotenoid present in microalgae, is well-recognized for its neuroprotection activities (Hu et al., 2018; Aziz et al., 2020). Fucoxanthin, is another prominent carotenoid present in microalgae, that can be produced and extracted simultaneously with LC-PUFAs from *Phaeodactylum tricornutum* (Pt) at an industrial scale (Delbrut et al., 2018; Wang et al., 2018). This is a distinctive feature of these microalgae, which in combination cannot be found naturally in other living organisms. Recently, the Food and Drugs Administration has approved the New Dietary Ingredient status of this microalgae extract with the following daily dose and duration recommendations for fucoxanthin: one month for an extract containing 8.8 mg/day, while doses 4.4 mg/day and lower can be taken daily indefinitely. Clinical evidence for efficacy of fucoxanthin in cognitive performance is limited, with one study demonstrating that 1 month of daily supplementation with a high dose of microalgae Pt extract containing 8.8mg of fucoxanthin improved several cognitive function parameters such as memory, executive function and perceptions of sleep quality in older individuals with self-reported cognitive decline (Yoo et al., 2024). Previous studies have also shown some benefits of a daily lower dose of Pt extract containing 4.4 mg of fucoxanthin on cognitive function, mood state and inflammation parameters in a mice model of aging and experienced gamers (Leonard et al., 2023; Maury et al., 2024). Therefore, we hypothesized that 6 months of daily supplementation with a microalgae Pt extract (Mi136) containing 4.4 mg of fucoxanthin would improve cognitive function, sleep quality and mood parameters and reduce blood inflammation status and perceived stress in healthy older adults with AAMI.

## 2 Materials and methods

### 2.1 Study design

**FIGURE 1:**
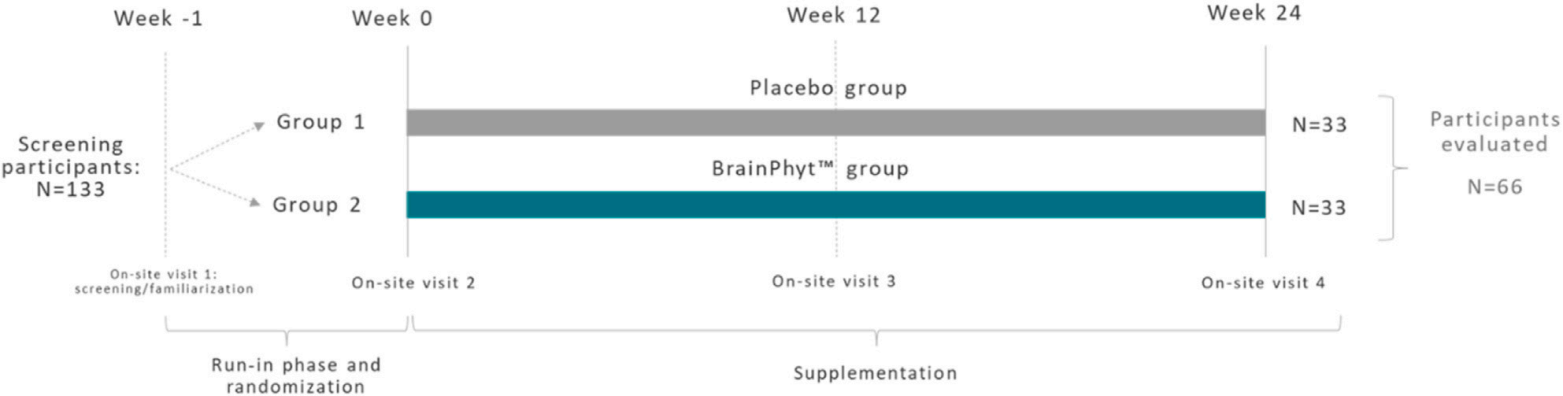
Overview of experiment study timeline for the placebo (PL) and experimental (Pt extract) groups.

This study was a randomized, double-blinded, placebo-controlled, parallel, single-centre clinical study. The total study duration per participant included a run-in phase followed by an intervention phase of 24 weeks (Figure 1).

The study was managed by Atlantia Clinical Trials company, an Irish contract research organization, and the recruitment and follow-up of participants took place in their capabilities (Atlantia Clinical Trials, Floor 1, Heron House, Blackpool, Cork, Ireland. T23 R^5^0 R). The Clinical Research Ethics Committee of the Cork Teaching Hospitals, Lancaster Hall 6 Little Hanover Street, Cork, approved the study protocol and associated documents. The study was conducted in accordance with the ethical principles set forth in the current version of the Declaration of Helsinki (seventh version, October 2013), the International Council for Harmonization (formerly the International Conference on Harmonization; ICH), the guidelines for Good Clinical Practice (ICH GCP, November 2016) and all applicable local regulatory requirements.

All participants provided written informed consent. Participants who met the eligibility criteria and successfully completed the 3–21-day run-in were randomized on a 1:1 basis to the two arms of this study to determine whether they received the study product or a placebo. Product assignment was conducted following a randomization list generated by an external statistician.

### 2.2 Study participants

**FIGURE 2:**
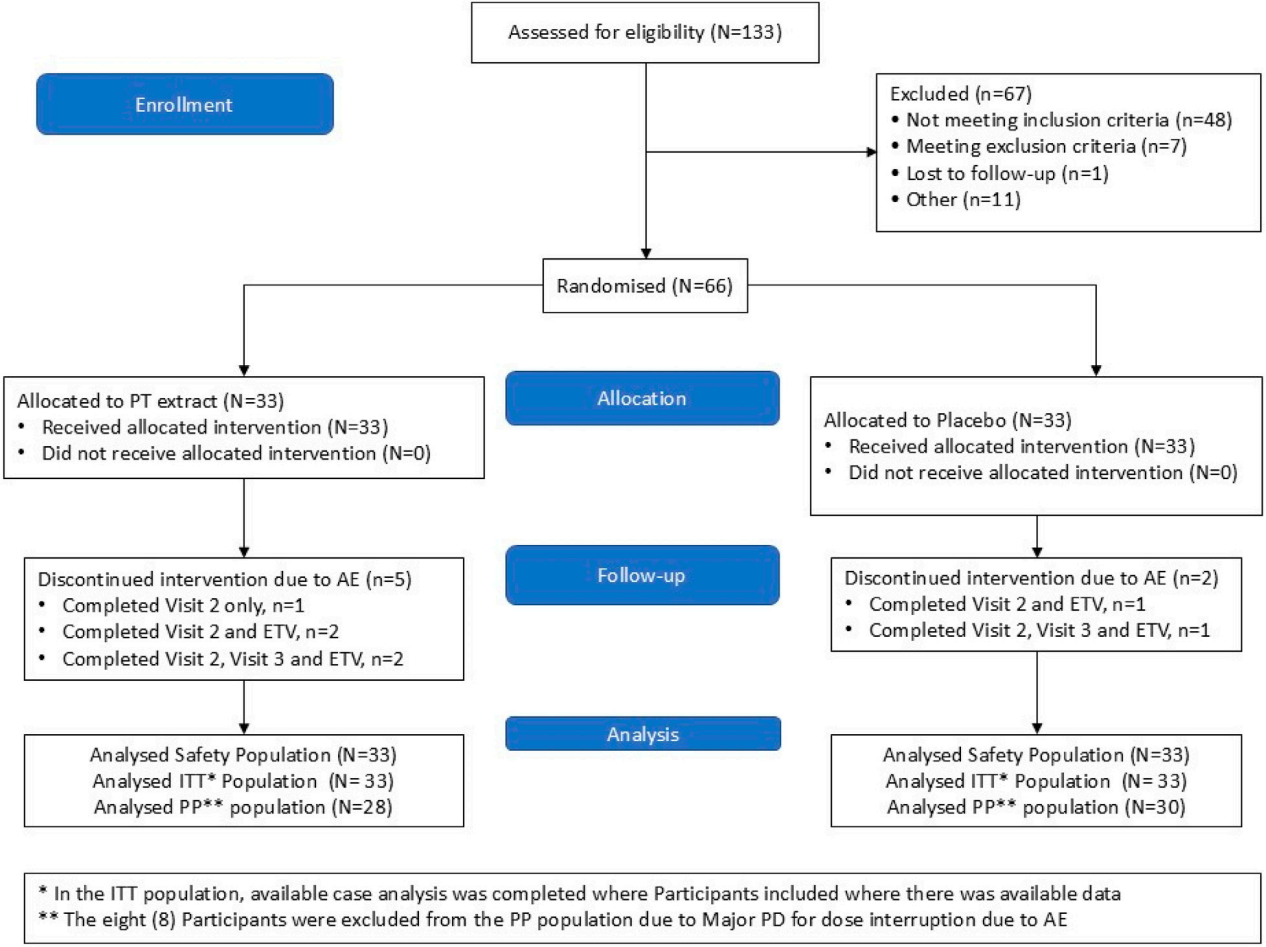
CONSORT diagram. A total of 133 participants were screened. Men and women with age-associated memory impairment were randomly assigned to one of the two treatments. A total of 33 subjects completed the study in both groups.

Participants were recruited between September 2022 and December 2022 (Figure 2. CONSORT diagram). The study population consisted of 66 healthy, free-living, older adults aged between ≥55 and ≤75 years, with age-associated memory decline (AAMI), defined as the absence of dementia as determined by a score of ≥24 on the Mini-Mental State Examination (MMSE) questionnaire and a score of ≥25 on the Memory Assessment Clinic-Questionnaire (MAC-Q), assessed at screening. Male and female participants were enrolled in the study. Significant consideration was given to record participant’s alcohol consumption based on alcoholic beverages as per Irish guidelines (Weekly low-risk alcohol guidelines, 2025), smoking status (number of packs per day from past and current smokers over life), and caffeine consumption as part of the exclusion criteria. In addition to their sex and age, participants were also asked about their ethnicity, education, and marital status. Individuals were included if they self-reported memory decline, were able to comply with the study protocol, were willing to maintain habitual diet and exercise routines and had a consistent sleep duration the evening before study visits. Participants were deemed ineligible if they were diagnosed with dementia, anxiety and depression, if they were pregnant, breastfeeding, wished to become pregnant during the study or were not using an effective method of contraception, were taking supplements recognized to improve cognitive function within 4-week of randomization, were using oral or injectable corticosteroids, had uncontrolled hypertension, diabetes, significant cardiovascular complaints, or significant neurological disease, planned a major lifestyle change, had a history of alcohol or substance abuse in the previous 12 months, had a history of heavy smoking in the last 3 months or had a history of heavy caffeinated beverage consumption in the previous 2 weeks. If the participants fully completed the study without major protocol deviations, they received a total of €450.

### 2.3 Study product and supplementation

The study product was a commercially available patented natural ingredient derived from microalgae: BrainPhyt™ (Mi136). It primarily comprises Fucoxanthin (FX), a specific marine carotenoid pigment and omega-3 long-chain polyunsaturated fatty acids (LC-PUFA). The production composition has been previously published (Maury et al., 2024). The placebo product used was maltodextrin. Participants were expected to consume two capsules daily for 24 weeks. Two capsules equated to either 550 mg (2*275 mg) of BrainPhyt™ (0.8% FX) or 600 mg (2*300 mg) maltodextrin. The placebo was manufactured to mimic the experimental supplement’s appearance, smell and taste. The product manufacturers issued a certificate of analysis verifying the absence of contaminants and the dosage. The dose selected in the present study was based on (1) the United States Food and Drug Administration approved dosage and duration in a context of New Dietary Ingredient application and (2) the results from the pre-clinical dose-response study conducted in a mice model of ageing (Maury et al., 2024).

### 2.4 Outcome measures

#### 2.4.1 Cognitive assessment

The study used the COMPASS Cognitive Assessment System to assess cognitive function components such as spatial working and episodic memories, attention and vigilance or executive function. The COMPASS cognitive test battery comprised a range of tasks utilising pictorial and verbal stimuli to assess cognitive function. Following previously established methods, these included the Corsi Block Task, Digit Vigilance Task, Choice Reaction Time Task, Word Recall Task, Picture Recognition Test, Word Recognition Task, and the Stroop Color–Word Test (Yoo et al., 2024).

As the primary endpoint, the Corsi Block Task Test assessed spatial working memory and attention by requiring participants to memorise and accurately reproduce sequences of blue squares on a grid. The Word Recall Task evaluated episodic memory by prompting participants to recall and record words within a set timeframe. Similarly, the Word Recognition Task and Picture Recognition Test measured episodic memory by distinguishing target stimuli from distractors. The Choice Reaction Time Task assessed response speed and vigilance by requiring participants to identify the direction of prompted arrows. The Digit Vigilance Task evaluated sustained attention and vigilance by prompting responses to varying numerical sequences displayed on-screen. Finally, the Stroop Color–Word Test assessed cognitive attention, executive function and processing speed by presenting color-naming challenges, where participants identified the font color of color-labeled words. This computerized testing battery is validated for age-associated memory impairment (AAMI) populations and is sensitive to acute and chronic nutritional interventions (Herrlinger et al., 2018).

#### 2.4.2 Mood, stress and sleep assessments

The Bond-Lader Mood Rating Scale (Bond-Lader), Leeds Sleep Evaluation Questionnaire (LSEQ) and Cohen’s Perceived Stress Scale (PSS) were administered at weeks 0, 12 and 24.

Bond-Lader scales reflect three key mood factors: calmness, contentment and alertness. LSEQ is a subjective evaluation of sleep and results in four domains: ease of getting sleep, quality of sleep, awakening from sleep and behavior following wakefulness.

#### 2.4.3 Biological samples

A non-fasting blood sample was collected at each study visit to analyze the safety profile. Further, at visits 2, 3 and 4, the sample was analysed for a range of biomarkers (IL-6, TNF-α, IFN-α, C-reactive protein - CRP).

#### 2.4.4 Safety and product compliance assessments

All unused study product returned by the participants at each visit was used to monitor overall product compliance. Consumption of at least 80% was deemed compliant.

Safety and tolerability of the study product were assessed by monitoring the occurrence of any intervention-emergent adverse events (AEs/SAEs), haematological safety parameters, biochemical blood safety parameters, vital signs and participant weight at each study visit.

### 2.5 Statistical analysis

The SPSS IBM V 28.0 software was used to conduct all the analyses on the intent-to-treat (ITT) and Per-Protocol (PP) population, and graphics were produced using R Project for Statistical Computing Version 4.3.0.

The sample size was determined based on a literature review of studies evaluating the effect of microalgae-based ingredients containing fucoxanthin, paraxanthine, ashwagandha or spearmint on cognitive function measures (Herrlinger et al., 2018; Xing et al., 2021; Leonard et al., 2023; 2024). For example, Herrlinger et al. showed that a composite score evaluating spatial working memory (including the Corsi Block test) increased from 1.61 to 1.81 after 3 months of supplementation (Herrlinger et al., 2018). Based on this review, we determined a minimum 10% improvement in cognitive test performance, particularly in Corsi Block Span Score (primary endpoint), with 80% power required. To assess delta change from baseline to Week 24 between the two product groups, a minimum of 30 participants in each product group was needed. A 10% drop-out rate was then built into this calculation. Therefore, the minimum total sample to be randomized was 66 participants.

Summary statistics were provided for continuous and categorical data. The minimum and maximum statistics, mean, median, quartiles and standard deviation were presented for continuous data. Counts and percentages were used for categorical data in frequency and relative frequency tables. The denominator for each percentage was the number of participants within each product group with available data. All analyses requiring significance testing were two-sided at a 5% significance level. Results were viewed as statistically significant if the p-value was less than 0.05. Significance testing was reported with the test statistic, p-value and Cohen’s d effect size. Cohen classified effect sizes as small (d = 0.2), medium (d = 0.5), and large (d ≥ 0.8) (Cohen, 1988). P values are sensitive to sample size and may fail to detect a meaningful effect when the sample size is small. Therefore, as this was a pilot exploratory study, the effect size and clinical review of descriptive summary statistics of mean change and standard deviations were reviewed to aid in the understanding of the hypothesis testing.

Due to the parallel design, the baseline profile of the sample was assessed to determine if there were any statistically significant and clinically meaningful between-group differences (Pt extract vs Placebo) at baseline. The normality of the data was evaluated using the Shapiro-Wilks test. The normality of the data was evaluated using the Shapiro-Wilks test. If this test was significant (p < 0.05), it was viewed that the normality assumption was violated, and the non-parametric alternative was implemented for efficacy analysis. Multiple comparisons were not controlled for in this study. No adjustment is required for the primary endpoint since it is a single endpoint. The secondary and exploratory endpoints may support the primary endpoint, but were considered mutually exclusive and were not corrected for multiple endpoints.

The primary analysis method was an ANCOVA, which was used to determine whether there was a statistically significant difference in change from baseline to Week 24 between product groups while controlling for baseline value. Δ Change Week 24 value was the dependent variable, Product Group was the independent variable, and Week 0 value was the covariate. For non-parametric data, Quade’s Rank ANCOVA was used.

A within-group Posthoc analysis was run to aid the interpretation of the data when the primary analysis had a between-group Cohen’s d ≥ 0.2 (Cohen, 1988). Paired t-tests were used to determine whether there was a statistically significant within-product change from Visit 2 (Week 0) to Visit 4 (Week 24) in the Pt Extract (Active) Group and Placebo Group, respectively, for the efficacy outcome analysis. For non-parametric data, the Wilcoxon Signed Rank test was used.

## 3 Results

### 3.1 Demographic and baseline data

Week 0 demographics data are presented in Table 1. Independent samples t-tests and Mann-Whitney U tests, as appropriate to the normality of the data, showed that there were not any statistically significant differences (p > 0.05) at Week 0 between the Pt and Placebo groups in all parameters. Baseline comparisons on cognitive function parameters showed a significant difference in Stroop–Overall reaction time parameter between the 2 study groups (p = 0.045), while no significant difference was reported for all other parameters.

**TABLE 1:** Demographic data.

Demographic variable	Placebo (n = 33)	Pt extract (n = 33)
Age (years)	64.2 (5.3)	63.6 (6.3)
Systolic blood pressure (mmHg)	132 (15)	131 (13)
Diastolic blood pressure (mmHg)	79 (9)	80 (10)
Heart rate (bpm)	69 (13)	71 (9)
Body weight (kg)	81 (22)	83 (19)
Body mass index (kg/m^2^)	29.2 (6.4)	29.9 (6.5)
Temperature (C)	36.35 (0.5)	36.48 (0.5)
Number of cigarette packs per day	0.81 (0.3)	0.78 (0.3)
Daily caffeine consumption (mg)	233 (91)	203 (95)
Number of standard drinks per week	3.83 (2.3)	4.48 (4.2)
Sex		
Female	23 (70%)	23 (70%)
Male	10 (30%)	10 (30%)
Ethnicity		
White - Any other White background	2 (6.1%)	3 (9.1%)
White - Irish	31 (94%)	30 (91%)
Smoking Status		
Current smoker	3 (9.1%)	3 (9.1%)
Non-smoker	22 (67%)	23 (70%)
Past smoker	8 (24%)	7 (21%)
Alcohol Consumption		
Consumes alcohol	29 (88%)	27 (82%)
Does not consume alcohol	4 (12%)	6 (18%)

Mean (SD), number (%).

### 3.2 Outcome measures

As shown in the Supplementary Material file, ITT and PP statistical analyses were conducted. As the main effects reported no differences when protocol non-compliers were excluded, we presented only the ITT analysis results in this manuscript.

#### 3.2.1 Cognitive assessment

Primary analysis on the primary endpoint (Corsi Block Span Score) and all other cognitive function parameters showed no significant between-group difference after 12 and 24 weeks. Within-group posthoc analysis revealed significant improvement in Stroop (overall reaction time) and Word Recall tasks, as summarized in Table 2 and detailed below. All the data analysis on COMPASS battery tests evaluated in the present study (Corsi Block, Attention and Vigilance reaction time, Digit vigilance, Stroop, Picture and Word recognition, Word recall) were presented and detailed in the Supplementary Tables S1.1–7.6.

**TABLE 2:** Descriptive statistics and treatment differences for stroop overall reaction time and delayed word recall.

Variable(s)	Placebo, N = 33				Pt extract, N = 33				Treatment difference at week 24, controlling for baseline score		
	Week 0	Week 12	Week 24	Within group P value*	Week 0	Week 12	Week 24	Within group P value*	Cohen’s d effect size	Test statistic	P Value
Stroop overall reaction time	1,460.7 (367.6)	1,325.4 (417.6)	1,352.1 (467.9)	0.06	1,368.6 (672.1)	1,166.2 (256.9)	1,111.9 (239.7)	<0.05	0.51	F = 3.62	0.06
Delayed word recall	3.3 (2.5)	4.66 (2.7)	4.13 (2.1)	0.15	3.36 (2.1)	4.46 (2.5)	4.61 (2.9)	0.01	0.26	F = 0.89	0.35

*Change from Week 0 to Week 24.

**FIGURE 3:**
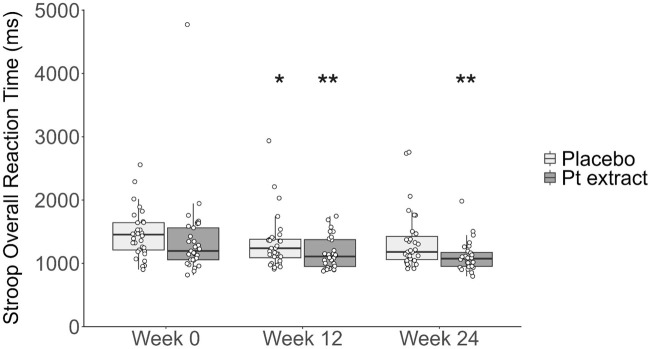
Stroop overall reaction time (ms). The Pt extract did not significantly reduce Stroop overall reaction time at the 12 week timepoint (F(1, 57) = 0.002, *p* = 0.968, partial η^2^ = 0.000) or 24 week timepoint (F(1, 56) = 3.615, *p* = 0.062, partial η^2^ = 0.061) as determined by ANCOVA. When the Wilcoxon signed-rank test was completed for a within-group analysis, the Pt extract significantly reduced stroop overall reaction time at week 12 (*p* = -0.048, z = 1.981 with a small to medium effect size, Cohen’s d = 0.26) and at week 24 (p = 0.005, z = 2.801 with a medium effect size, Cohen’s d = 0.37). Within the placebo group, a decrease in reaction time was seen at week 12 (p = 0.008, z = 2.655 with medium effect size, Cohen’s d = 0.33), while the effect was not apparent at week 24 (z = 1.901, p = 0.057 with a medium effect size, Cohen’s d = 0.24). *p < 0.05; **p < 0.01 as compared with the respective groups at baseline week 0.

The ANCOVA model, controlling for baseline Stroop Overall Reaction Time, showed a trend to significant treatment differences in change in Overall Reaction Time from baseline to Week 24 (F = 3.62, *p = 0.06*) with a Cohen’s d effect of 0.51 indicating a medium effect size. No significant between-group difference was reported after 12 weeks of supplementation. Wilcoxon Signed-Rank Test was used to separately evaluate the within-group change from Week 0 to Week 24 in the Pt extract and placebo groups. As shown in Figure 3, there was a statistically significant within-group reduction in Stroop Overall Reaction Time from Week 0 to Week 12 in both groups. However, the decrease was significant at Week 24 (z = 2.801, p = 0.005) with a large effect size (Cohen’s d = 0.81) for only the Pt extract group. The placebo group did not have a statistically significant reduction, Week 0 to Week 24 (z = 1.901, p = 0.057) and a medium effect size (Cohen’s d = 0.50). Percentage Correct Responses in the Stroop Task were also assessed, and there were no statistically significant results due to ceiling effects at baseline.

**FIGURE 4:**
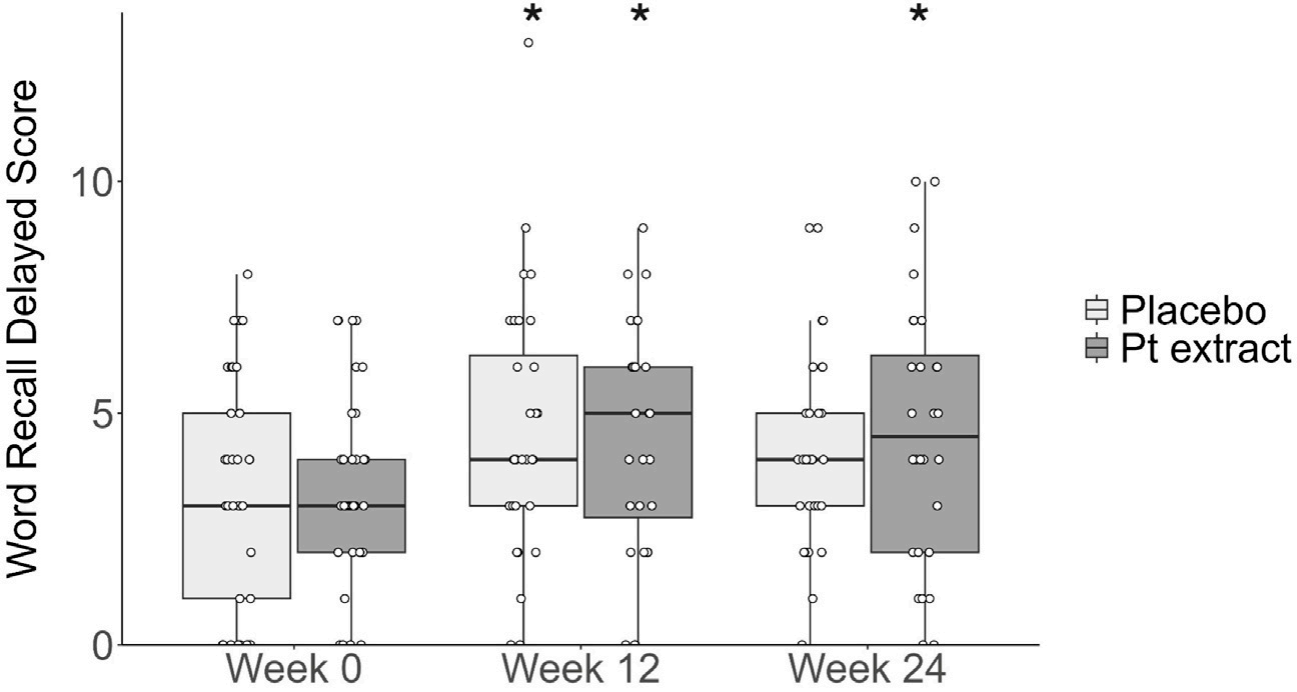
Delayed word recall score. The Pt extract did not significantly improve delayed word recall score at week 12 (F(1, 57) = 0.112, p = 0.739, partial η^2^ = 0.002) or week 24 (F(1, 56) = 0.886, p = 0.351, partial η^2^ = 0.016) as determined by ANCOVA. From the within-group analysis, paired t-tests showed that the Pt extract significantly improved delayed word recall score at week 12 (t(27) = 2.714, p = 0.011, with a large effect size, Cohen’s d = 0.51), and week 24 (t(27)=2.775, *p* = 0.010, with a large effect size, Cohen’s d = 0.53) as compared with baseline week 0. Within the placebo group, an increase was observed at week 12 (t(27) = 2.491, p = 0.018, with a medium to large effect size, Cohen’s d = 0.44) but there was no significant difference at week 24 (t(27) = 1.478, p = 0.150, with a small effect size, Cohen’s d = 0.266). *p < 0.05 as compared to the respective baseline control at week 0.

The Quade’s ANCOVA model, controlling for baseline Delayed Word Recall, showed that there was not a statistically significant difference in change in Delayed Word Recall from baseline to Week 24 (F = 0.89, *p = 0.35*) with a Cohen’s d effect of 0.26 indicating a small effect size. No significant between-group difference was reported after 12 weeks of supplementation. Paired-sample t-tests assessed within-group changes in Delayed Word Recall scores from Week 0 to Week 24 for the Pt extract and placebo groups, respectively. As shown in Figure 4, there was a statistically significant improvement in Delayed Word Recall from Week 0 to Week 24 (t (27) = 2.775, p = 0.010, CI = 0.317–2.112) with a medium effect size (Cohen’s d = 0.53) only in the Pt extract group. For the placebo group, there was not a statistically significant change in Delayed Word Recall from Week 0 to Week 24 (t (30) = 1.478, p = 0.150, CI = −0.246–1.536) with a small effect size (Cohen’s d = 0.27).

#### 3.2.2 Biological samples

**FIGURE 5:**
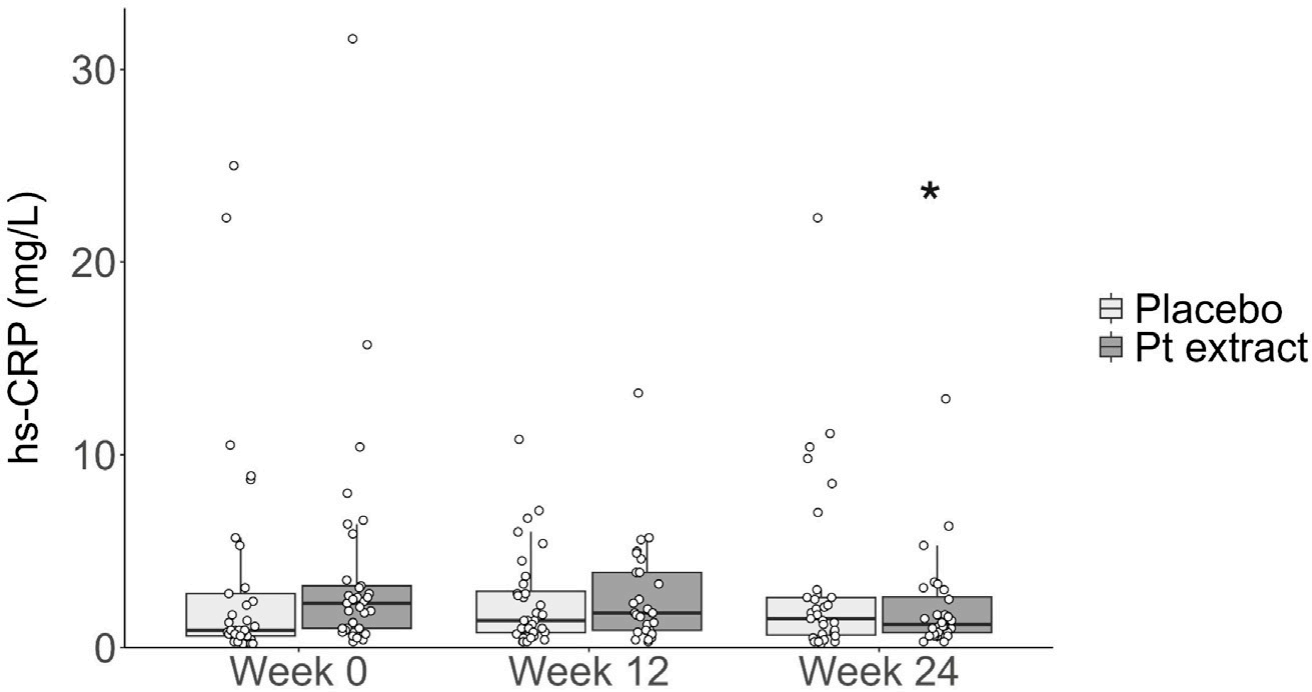
Levels of hs-CRP (mg/L). The Pt extract group significantly reduced hs-CRP as determined by Quade’s ANCOVA at week 24 (F = 10.20, p = 0.002, with a large effect size, Cohen’s d of 0.84) but not at week 12 (F = 1.517, p = 0.223, with a small effect size, Cohen’s d effect of 0.23). When the Wilcoxon signed-rank test was completed for a within-group analysis, the Pt extract significantly reduced hs-CRP at week 24 (z = 3.098, p = 0.002, with a large effect size Cohen’s d = 0.91) but not at week 12 (z = 1.408, p = 0.159, with a small effect size (Cohen’s d = 0.19). The effect was not apparent in the placebo group at both week 12 (z = 0.059, p = 0.953 with a very small effect size, Cohen’s d = 0.01) and week 24 (z = -1.828, p = 0.068 with a small effect size (Cohen’s d = 0.23). *p < 0.05 as compared with Pt extract baseline week 0.

The results from inflammation biomarkers are detailed in the Supplementary Tables S11.1–S13.3. Significant between and within-group effects were shown only in blood Hs-CRP (Table 3 and Figure 5) level.

**TABLE 3:** Descriptive Statistics and Treatment Differences for hs-CRP.

Variable(s)	Placebo, N = 33				Pt extract, N = 33				Treatment difference at week 24, controlling for baseline score		
	Week 0	Week 12	Week 24	Within group P value*	Week 0	Week 12	Week 24	Within group P value*	Cohen’s d effect size	Test statistic	P Value
hs-CRP	3.4 (5.9)	2.4 (2.5)	3.3 (4.7)	0.07	3.9 (5.9)	2.6 (2.7)	2.1 (2.6)	<0.05	0.84	F = 10.21	0.002

*Change from Week 0 to Week 24.

Quade’s ANCOVA model, controlling for baseline hs-CRP, showed that there was a statistically significant difference in change in hs-CRP from baseline to Week 24 (F = 10.20, *p = 0.002*) with a Cohen’s d effect of 0.84 indicating a very large effect size. Wilcoxon signed-rank tests demonstrated a statistically significant reduction in hs-CRP levels in the Pt group from Week 0 to Week 24 (z = 3.098, p = 0.002) with a large effect size (Cohen’s d = 0.91). There was a trend to increase in hs-CRP level from Week 0 to Week 24 in the Placebo group, but this was not statistically significant (z = −1.828, p = 0.068) with a small-medium effect size (Cohen’s d = 0.48).

#### 3.2.3 Mood, stress and sleep assessments

Results from exploratory endpoints related to mood, stress and sleep assessments are presented in the Supplementary file (Supplementary Tables S8.1–S10.5). The main effects of these parameters are presented below.

**FIGURE 6:**
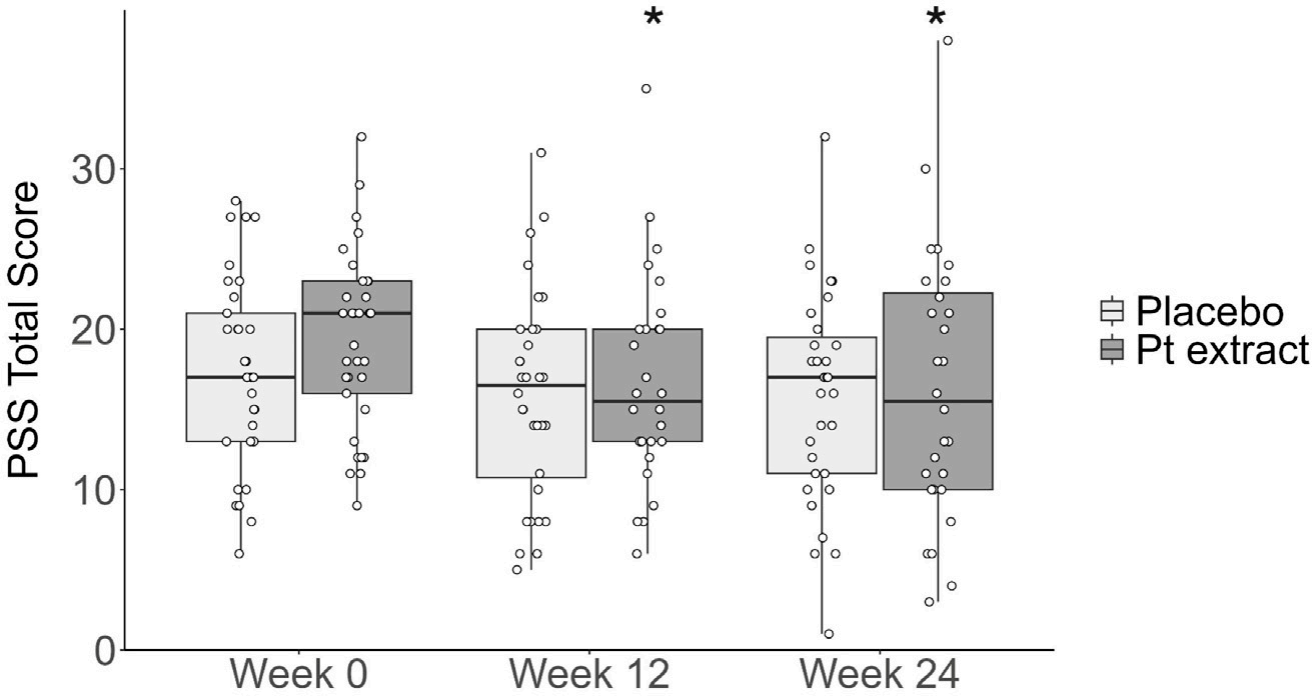
Perceived stress scale (PSS) total score. The Pt extract did not significantly reduce PSS total score at week 12 (F(1, 57) = 0.007, p = 0.932, partial η^2^ = 0.000) or week 24 (F(1, 56) = 0.151, p = 0.699, partial η^2^ = 0.003) as determined by ANCOVA. From the within-group analysis, paired t-tests showed that the Pt extract significantly reduced PSS total score at both week 12 (t[27] = −2.06, p = 0.049, with a medium effect size, Cohen’s d = 0 0.39) and at week 24 (t[27] = −2.14, p = 0.041, with a medium effect size, Cohen’s d = 0 0.41). The effect was not evident in placebo group where placebo group did not significantly reduce PSS total score at either week 12 (t[31] = −1.420, p = 0.166, with a small effect size, Cohen’s d = 0 0.251) or week 24 (t[30] = −1.188, p = 0.244, with a small effect size, Cohen’s d = 0 0.213). *p < 0.05 as compared with Pt extract baseline control at week 0.

As shown in Table 4 and Figure 6, Quade’s ANCOVA model, controlling for baseline PSS Score, showed that there was not a statistically significant difference in change in PSS Score from baseline to Week 24 (F = 0.151, *p = 0.699*) with a Cohen’s d effect of 0.11 indicating a very small effect size. No significant difference between groups was reported after 12 weeks of supplementation. Paired-sample t-tests assessed within-group changes in PSS Total Score from Week 0 to Week 12 and 24 for the Pt extract and placebo groups, respectively. There was a statistically significant reduction in PSS Total Score from Week 0 to Week 12 in the Pt extract group (t (27) = −2.06, p = 0.049, CI = −5.056 to −0.015), with a small to medium effect size (Cohen’s d = 0 0.39). There was also a statistically significant reduction in PSS Total Score from Week 0 to Week 24 in the Pt extract group (t (27) = −2.14, p = 0.041, CI = −5.664 to −0.122), with a small to medium effect size (Cohen’s d = 0 0.41). There was no statistically significant change in PSS Total Score from Week 0 to Week 12 (t (31) = −1.42, p = 0.166) or Week 0–24 (t (30) = −1.19, p = 0.244) in the Placebo group and a small effect size (Week 12 Cohen’s d = 0.25; Week 24 Cohen’s d = 0.21).

**TABLE 4:** Descriptive statistics and treatment differences for PSS.

Variable(s)	Placebo, N = 33				Pt extract, N = 33				Treatment difference at week 24, controlling for baseline score		
	Week 0	Week 12	Week 24	Within group P value*	Week 0	Week 12	Week 24	Within group P value*	Cohen’s d effect size	Test statistic	P Value
PSS	17.3 (5.9)	15.8 (5.5)	15.8 (6.6)	0.24	19.3 (5.5)	16.6 (5.8)	16.3 (8.3)	0.04	0.11	F = 0.151	0.699

*Change from Week 0 to Week 24.

Regarding Bond-Lader Mood Rating (BLMR) score data, no significant between-group change was detected for the subscales of Alertness, Contentment, and Calmness (Supplementary Tables S9.1–S9.8). Data for Leeds Sleep Evaluation Questionnaire (LSEQ) scores by treatment for the LSEQ Subscales of Getting to Sleep (GTS), Quality of Sleep (QOS), Awakening from Sleep (AFS) and Behaviour following Wakefulness (BFW) from Baseline (Week 0) to the End of Intervention (Week 24) are provided in Supplementary Tables S10.1–S10.5. No significant change was detected either within or between groups.

### 3.3 Safety and product compliance

Data on adverse events, vital signs and blood safety parameters were detailed in Supplementary materials (Supplementary Tables S14.1–S14.38), and no between-group difference was reported. During this study, no participant experienced abnormal clinically significant changes to any vital signs relating to consumption of the active product (Pt extract), and only one clinically significant change from baseline (SAE) was recorded. This SAE was an abnormal gamma-glutamyltransferase level change from baseline for a participant in the Pt group. It was after, though unlikely to be a consequence of, consumption of the IP that it was marked for investigation. Laboratory results recorded a large panel of blood safety parameters falling within normal ranges. Post-screening, laboratory results were reviewed by clinical personnel throughout the study period and deemed to be safe at all time points. As shown in Figure 2, eight participants withdrew from this study, with five of these participants in the Pt extract group. Of the five participants in the Pt extract group, only three participants had AEs that were deemed related to IP, which led to the participant discontinuing the study. These AEs were mild to moderate, with only one mild “Nausea” AE that did not resolve by time of discontinuation.

## 4 Discussion

This proof-of-concept study examined whether ingesting a microalgae extract (550 mg of *Phaeodactylum tricornutum* with 0.8% of fucoxanthin, “Pt. extract”) improves cognitive function, stress, mood, sleep quality and neuroinflammation parameters in healthy older adults with AAMI. Despite the fact that the primary statistical analysis showed no between-group effect in all cognitive function parameters, results from within-group Posthoc analysis provide promising data that a 24-week regimen of Pt extract may positively affect cognitive function parameters relating to executive function, attention and episodic memory. Moreover, this microalgae-based ingredient may impact stress and inflammation parameters. A noteworthy finding is the reduction in blood Hs-CRP, which may be a biomarker for cognitive decline (Lewis and Knight, 2021). These data support the conclusions from previous pre-clinical and clinical studies showing treatment benefits on cognition parameters and inflammatory status (Leonard et al., 2023; Maury et al., 2024; Yoo et al., 2024).

Among the health issues arising from an aging population, maintaining cognitive functioning will be a major challenge, both in societal and economic terms. Research has documented the negative consequences of age-related cognitive function decline for healthy adults (Salthouse, 2012). Cognitive and memory abilities are essential for instrumental activities of daily living, and a decline in performance across these activities, such as understanding household product labels and medical instructions, as well as activities such as shopping and transportation, is associated with adverse quality of life outcomes. The present study’s findings highlight several positive statistically significant improvements within the Pt extract group, that were not apparent in the placebo group. In the absence of a crossover design study, such findings are relevant as it highlights changes within a population as a consequence of treatment. The lack of significance between groups could be an artefact of demographic selection, degree of age-associated cognitive impairment or rate of progression of this impairment and a relatively high degree of variance in several of the readouts associated with this study. These include cognitive function components such as executive function, attention, vigilance and episodic memory. The most promising results were for Stroop Reaction Time, which assesses executive function, attention and vigilance. There was a significant within-group improvement in the Pt extract group for the Stroop task for reaction time after 24 weeks, with no significant within-group change in Placebo. These areas of cognition are vital for maintaining effective working memory, cognitive flexibility and inhibition skills. These data are encouraging and closely reflect the findings of previous studies in plant extract supplementation with astaxanthin, citicoline, Bacopa monnieri and Ginkgo biloba (Liu et al., 2019; McPhee et al., 2021; Queen et al., 2024). For the Stroop task, the Pt extract group showed a within-group mean decrease of 257 m in overall reaction time from baseline to 24 weeks. These results were comparable to published literature showing evidence for beneficial effects in older adults. It should be noted that in contrast to the present study, researchers often report improvements in Stroop performance for older adults but do not provide details on Stroop reaction time and accuracy (Stavrinou et al., 2020; Tokuda et al., 2020). When studies report Stroop reaction time, the improvement after supplementation is considerably less than in our current study. Cave et al. reported in a 3-month study in healthy adults (age: 18–50 years) a mean decrease of 17 ms in Stroop reaction time following supplementation with spearmint extract (from Mentha spicata) (Cave et al., 2023). Similarly, Lopresti et al. reported a decrease of 21 ms in Stroop Reaction time following a 6-month supplementation with lutein and zeaxanthin in older adults (71 females and 19 males, age 40–75) with self-reported cognitive decline (Lopresti et al., 2022).

Furthermore, the present study also showed significant improvements in immediate and delayed word recall scores following 3 and 6 months of microalgae Pt extract supplementation, highlighting the enhancement in working and episodic memory. Together, these data strengthen the position that active ingredients used in the present study have meaningful benefits to the target older population, supporting our previous works (Leonard et al., 2023; Maury et al., 2024; Yoo et al., 2024). It was previously shown that 12 weeks of supplementation with a higher dose of microalgae Pt extract (containing 8.8mg/day of fucoxanthin) improved some parameters for the Stroop color-word and word-recall tests (Yoo et al., 2024). In addition, an acute and 30-day supplementation with Pt extract containing 4.4 mg/day of fucoxanthin in combination with guarana improved executive function, attention shifting (cognitive flexibility) and memory among experienced gamers (Leonard et al., 2023).

Although the present study demonstrated some promising findings and beyond the need of a full-scale definitive trial to highlight significant between-group differences, we failed to show benefits from Pt extract supplementation in other cognitive tasks such as Corsi block, Digit vigilance and Picture/Word recognition tasks. Several potential explanations exist for why significant improvement was not found for these cognition outcomes. Firstly, the dosage may be below the threshold required. Herrlinger et al. evaluated the dose-response effect of a spearmint extract supplementation on working and spatial memory using the Corsi block task and found that a higher dose was required to see a statistically significant improvement (Herrlinger et al., 2018). Similarly, we previously showed in a mice model of aging that a microalgae Pt extract only partially improved spatial cognitive function (mean spontaneous alternation behavior) impairment induced by D-Gal chronic intoxication with the lower dose (120 mg/day) while it was fully inhibited using the three higher doses (from 235 to 370 mg/day) (Maury et al., 2024). However, our recent study evaluating the effect of higher dose Pt extract (containing 8.8 mg/day) also did not identify benefits using the Corsi Block task after both 12 and 24 weeks of supplementation, suggesting that this cognitive function test may not have sufficient sensitivity to detect change in a healthy elderly population. Secondly, as evidenced by the ceiling effects observed in several cognitive assessment parameters, particularly on the Digit Vigilance and Picture/Word recognition tasks, our sample may have been too high functioning at baseline. Although no training session before the study started was conducted, which may represent a potential bias, it is clear that participants had no significant difficulties with the cognitive tests.

The target population of the current study was a healthy, free-living population with age-associated memory decline. The memory decline in this study was measured using the MMSE and MAC-Q. The MMSE is validated to measure cognitive impairment and is commonly used in clinical settings to screen for dementia so that it may have lacked the sensitivity required for the present study sample. While the MAC-Q was selected to address this issue during the design phase, as it is validated to assess age-related cognitive decline, it may not have been sufficient to offset the limitations of the MMSE. In future studies, the inclusion criteria should include the MAC-Q and a COMPASS assessment baseline cut-off score to counter-act the risk of ceiling effects. Thirdly, the COMPASS tasks in the battery of assessments were validated on clinical populations, and some tasks may lack the sensitivity required for a healthier population. Many tasks and assessment methods were validated on patients diagnosed or presenting with symptoms of some degree of cognitive impairment. Estrada-Orozco et al. have discussed the validation of various cognitive tests with suggested areas for refinement of assessment methods, particularly for younger, healthier individuals (Estrada-Orozco et al., 2018). The cognitive tasks should be selected based on their difficulty level and assessment type for future studies investigating the effects of supplements or dietary interventions. To summarise and consider the main potential study limitations identified, a fully scaled trial is needed to confirm the present promising data by taking into account the following Methods insights: 1/Sample size calculation based on Stroop task overall reaction time as primary endpoint; 2/Stratified participant cohort on baseline cognitive function abilities and consider the inclusion of subgroup participants with neurological diseases; 3/Evaluate several Pt extract doses and 4/Select more sensitive cognitive function tests to change in healthy participants based on literature analysis.

We also found a very promising result in blood hs-CRP, showing a statistically significant between-group improvement in the Pt extract group compared to the Placebo after 6 months of supplementation. The mean blood hs-CRP level recorded in the current study is similar to clinical normal levels, as reported by Wyczalkowska-Tomasik et al., who assessed levels of CRP over a broad range of ages, noting increased average levels with increasing age (Wyczalkowska-Tomasik et al., 2016). A baseline hs-CRP level of 3.92 mg/L was recorded (corresponding to a mean of range 60–70 years), which decreased to 2.3 mg/L (corresponding to a mean of range 50–60 years) following 24 weeks of Pt extract supplementation while no change in the placebo group. In this current study, the mean age of the population in the present study is around 64 years. Previous studies have demonstrated a significant association between increased hs-CRP concentration, cognitive function and long-term cognitive decline (Simen et al., 2011; Lin et al., 2018; Zheng and Xie, 2018; Kipinoinen et al., 2022). While some underlying mechanisms remain unclear, inflammation and oxidative stress appear important in age-related cognitive decline (Tönnies and Trushina, 2017; Scarmeas et al., 2018). A review by McGrattan et al. summarized the role of chronic activation of microglial macrophages in the brain, inducing sustained production of pro-inflammatory cytokines, such as IL-6, TNF-α and CRP, predictive of worsening cognitive function (McGrattan et al., 2019). The cycle of neuroinflammatory processes, consequent to the increase in levels of these cytokines, can lead to a reduction in brain volume or cortical thinning (Gu et al., 2017). In a pre-clinical study, we showed that Pt extracts significantly decreased brain and blood levels of inflammatory markers such as TNF-α, IL-6 and also lipid peroxidation following D-GAL induction in mice in parallel with cognitive function improvement with a dose-response effect (Maury et al., 2024).

The microalgae Pt extract evaluated in the present study contain several molecules of interest which are well known in regulating inflammatory pathways involved in cognitive function activities. Fucoxanthin exhibits antioxidant and anti-inflammatory activity. Following ingestion, digestive enzymes rapidly metabolise it to fucoxanthinol before absorption in the intestines and then conversion to amarouciaxanthin A in the liver. Capable of bypassing the blood-brain barrier, fucoxanthin acts directly on microglial cells, inducing a decrease in MAPK phosphorylation, thereby inhibiting microglial secretion of IL-6, TNF-α, IL-1β, iNOX and COX-2 (Wyczalkowska-Tomasik et al., 2016; Zheng and Xie, 2018; Fernandes and Mamatha, 2023). It also restores neuronal homeostasis by Nrf2 pathway activation, allowing translocation of Nrf2 to the nucleus from the cytosol, where Nrf2 induces gene expression, which activates mechanisms of neuronal autophagy. The consequent elimination of intracellular waste products of oxidation aids in the reduction of subsequent oxidative stress. EPA and DHA are LC-PUFA from the omega-3 family, which are also essential in cognitive health, as the human brain comprises 60% fat, of which around half is DHA. The microalgae ¨Pt extract contains ±6% EPA, a PPAR alpha receptor agonist. One of EPA’s primary capabilities is its capacity to regulate eicosanoid hormone levels, thereby reducing age or injury-related inflammation and pain. Together, these potential mechanisms of action contribute to EPA’s neuroprotective effects. Beyond the need to conduct dedicated studies to understand better the precise mechanism of actions underlying the benefits of the microalgae extract on inflammatory pathways and cognitive function, the present data may warrant a longitudinal study over several years to evaluate the impact of Pt extract containing fucoxanthin on cognitive function decline as a preventive intervention in the healthy elderly. Lifestyle and dietary interventions show potential in the prevention of cognitive decline during aging, and studies have examined the effects of phytochemical nutritional supplements on cognitive function, including in participants with AAMI (Valls-Pedret et al., 2015; Lin et al., 2018; McGrattan et al., 2019; Kipinoinen et al., 2022). Several studies concluded that chronic low-grade inflammation was associated with decreased cognitive performance in later life. A previous study on the role of inflammation in age-related cognitive dysfunction showed that those with the highest levels of CRP had a greater incidence of episodic memory impairment and visuospatial impairment, though no greater rate of executive function or language impairment (Simen et al., 2011).

Patient-reported outcomes are increasingly recognized by regulatory bodies, clinicians, and industry as essential elements of clinical trial design as they provide the evaluation of intervention efficacy from a participant’s perspective. Stress was evaluated as exploratory outcomes using Cohen’s Perceived Stress Scale. There were statistically significant within-group improvements for stress in both Week 12 and Week 24 in the Pt group. At the same time, there was no change in the Placebo group. Specifically for Stress, there was a statistically significant (p = 0.041) mean decrease of 2.89 (Supplementary Table S8.1) in the PSS Stress score from baseline to Week 24 in the Pt extract group. In contrast, there was no statistically significant within-group change in the Placebo group. Stress is considered a major risk factor in age-related cognitive decline, with a 2012 study focusing on changes in cortisol levels and finding an association with thinning of prefrontal cortex regions (Kremen et al., 2012; Cicero et al., 2017). They also looked at the detrimental impact of chronic stress on cognitive aging. Cicero et al. reported that 2 months of treatment with a combination of Bacopa monnieri, L-theanine, Crocus sativus, copper, folate and B and D group vitamins improved PSS scores in a similar range to those in our study (Cicero et al., 2017). Meanwhile, Schön et al. showed the beneficial effects of a 4-month regimen of grape seed extract (from Vitis vinifera L. seeds), reporting statistically significant improvements in blood pressure and perceived stress level (Schön et al., 2021). Overall, the benefits shown in the present study are consistent with the current literature, and further studies are needed to refine the application of this microalgae Pt extract on stress and its impact on cognitive functions.

Safety and tolerability are essential in supplements, and the study participants tolerated this product well. Previous testing of this product has not revealed any toxicity or carcinogenicity (Leonard et al., 2023; Dickerson et al., 2024; Yoo et al., 2024). The Investigational Product was found to be safe and well tolerated in this study, and there is considerable scope for further development and utilisation of this supplement. Supporting the safety and tolerability, participants were deemed compliant if they had a product intake compliance of ≥80%. Product consumption in the study was good to excellent compared to other trials, with all of them having a product intake compliance of ≥80% as initially defined (Hubbard et al., 2012; Mallayasamy et al., 2018). However, it is important to note that no biomarker data were collected to confirm metabolite presence, which should be included in a future study.

In conclusion, the results are encouraging and suggest that a 6-month course of microalgae Pt extract containing 4.4 mg of fucoxanthin may improve cognitive function parameters such as executive function, attention, vigilance and episodic memory even if only within group effects were reported. Furthermore, there is evidence that microalgae Pt extract reduced levels of hs-CRP, a marker of inflammation widely recognized as a predictive factor of cognitive decline in a healthy ageing population. There was also evidence that microalgae Pt extract may improve perceived stress and mood state. While further research is needed with a larger cohort to confirm these results, this proof-of-concept study has the merit of providing initial proof of the efficacy of low daily supplementation with microalgae extract from *Phaeodactylum tricornutum* containing only 4.4 mg of fucoxanthin during 24 weeks, opening up the prospect of evaluating novel research hypotheses to prevent age-related cognitive decline.

## Data Availability

The raw data supporting the conclusions of this article will be made available by the authors, without undue reservation.
