# Supplementary material for: Promising benefits of six-month Phaeodactylum tricornutum microalgae supplementation on cognitive function and inflammation in healthy older adults with age-associated memory impairment
Source: Front Aging. 2025 Apr 30;6:1540115. doi: 10.3389/fragi.2025.1540115 (PMC12075122; doi:10.3389/fragi.2025.1540115)
Supplement: Supplementary file 1 [file Table1.docx]

# SPATIAL WORKING MEMORY COMPASS CORSI BLOCK SPAN SCORE

## Descriptive Analysis

Table 1.1 Summary Descriptive Statistics for Corsi Block Span Score by product at Baseline (Week 0; Visit 2) and End of Intervention (Week 24; Visit 4) in the ITT Population (N=66)

| **Product Corsi Block – Span Score** | | **N** | | **Mean** | **SEM** | **SD** | **Min** | **Quartiles** | | | |
| --- | --- | --- | --- | --- | --- | --- | --- | --- | --- | --- | --- |
|  |  | **Valid** | **Missing** |  |  |  |  | **Q1** | **Mdn** | **Q3** | **Max** |
| PT extract | Week 0 | 33 | 0 | 3.81 | 0.41 | 2.35 | 0.00 | 2.00 | 4.67 | 5.50 | 7.00 |
|  | Week 12 | 28 | 5 | 4.21 | 0.34 | 1.82 | 0.00 | 3.00 | 4.67 | 5.33 | 7.00 |
|  | Week 24 | 28 | 5 | 4.72 | 0.26 | 1.40 | 1.33 | 4.42 | 4.67 | 5.67 | 6.67 |
|  | Change Week 12 | 28 | 5 | 0.15 | 0.39 | 2.09 | -3.00 | -1.00 | 0.00 | 0.59 | 4.67 |
|  | Change Week 24 | 28 | 5 | 0.66 | 0.37 | 1.96 | -2.66 | -0.67 | 0.17 | 1.34 | 5.00 |
| Placebo | Week 0 | 33 | 0 | 3.25 | 0.33 | 1.88 | 0.00 | 1.33 | 2.67 | 5.00 | 6.33 |
|  | Week 12 | 32 | 1 | 3.65 | 0.31 | 1.73 | 1.33 | 1.67 | 4.33 | 5.25 | 6.00 |
|  | Week 24 | 31 | 2 | 4.42 | 0.29 | 1.63 | 1.33 | 4.00 | 4.67 | 5.67 | 6.33 |
|  | Change Week 12 | 32 | 1 | 0.37 | 0.32 | 1.81 | -4.34 | -0.66 | 0.17 | 1.58 | 4.34 |
|  | Change Week 24 | 31 | 2 | 1.04 | 0.35 | 1.95 | -2.00 | -0.33 | 0.34 | 2.67 | 4.67 |

## Primary Analysis

Table 1.2 Mann-Whitney U Tests to Determine If There Were Between Product Differences In Change In Corsi Block – Span Score From Baseline (Week 0) To Week 12, From Baseline (Week 0) To Week 24, and From Week 12 To Week 24 In The ITT (N=66) and PP (N=58) Populations.

| **Between Groups*** | **Total**  **N** | **Mann-Whitney**  **U** | **Standardized**  **Test Statistic** | **Asymptotic Sig.**  **(2-sided test)** | **Effect**  **Size** |
| --- | --- | --- | --- | --- | --- |
| **Corsi Block Span Score** |  |  |  |  |  |
| Change Week 0 - Week 12 ITT | 60 | 384.5 | -.945 | .345 | 0.122 |
| Change Week 0 - Week 24 ITT | 59 | 392.5 | -.632 | .528 | 0.082 |
| Change Week 12 - Week 24 ITT | 59 | 420.0 | -.213 | .831 | 0.028 |
| Change Week 0 - Week 12 PP | 58 | 365.5 | -.851 | .395 | 0.112 |
| Change Week 0 - Week 24 PP | 58 | 377.0 | -.670 | .503 | 0.088 |
| Change Week 12 - Week 24 PP | 58 | 401.5 | -.288 | .773 | 0.038 |
| *Shapiro-Wilks Test of Normality p<0.05. Therefore Non-Parametric Mann-Whitney U Test was used. | | | | | |

Table 1.3 Related-Samples Wilcoxon Signed Ranks Tests to Determine If There Were Within Product Differences In In Change In Corsi Block – Span Score From Baseline (Week 0) To Week 12, From Baseline (Week 0) To Week 24, and From Week 12 To Week 24 In The ITT (N=66) and PP (N=58) Populations

| **Within Group*** | **Total**  **N** | **Standardized**  **Test Statistic** | **Asymptotic Sig.**  **(2-sided test)** | **Effect**  **Size** |
| --- | --- | --- | --- | --- |
| **Corsi Blocks Span Score** |  |  |  |  |
| **Week 0 - Week 12** |  |  |  |  |
| PT extract ITT | 28 | .049 | .961 | 0.007 |
| Placebo ITT | 30 | -1.307 | .191 | 0.169 |
| PT extract PP | 28 | .049 | .961 | 0.007 |
| Placebo PP | 30 | -1.307 | .191 | 0.169 |
| **Week 0 - Week 24** |  |  |  |  |
| PT extract ITT | 28 | -1.401 | .161 | 0.187 |
| Placebo ITT | 30 | -2.585 | .010 | 0.334 |
| PT extract PP | 28 | -1.401 | .161 | 0.187 |
| Placebo PP | 30 | -2.585 | .010 | 0.334 |
| **Week 12 – Week 24** |  |  |  |  |
| PT extract ITT | 28 | -1.778 | .075 | 0.238 |
| Placebo ITT | 30 | -1.710 | .087 | 0.221 |
| PT extract PP | 28 | -1.401 | .161 | 0.187 |
| Placebo PP | 30 | -2.585 | .010 | 0.334 |
| *Shapiro-Wilks Test of Normality p<0.05. Therefore Non-Parametric Wilcoxon Signed Ranks Tests was used. | | | |  |

## Sensitivity Analysis

Table 1.4 Quade Nonparametric Analysis of Covariance Tests to Determine There Were Between Product Differences In Change In Corsi Block – Span Score From Baseline (Week 0) To Week 12, From Baseline (Week 0) To Week 24, and From Week 12 To Week 24 In The ITT (N=66) and PP (N=58) Populations While Controlling for Baseline in the ITT and PP Population

| **Population** | **F** | **DFH** | **DFE** | **P Value** |
| --- | --- | --- | --- | --- |
| **Corsi Block – Span Score** |  |  |  |  |
| Week 0 – Week 12 ITT | 0.000 | 1 | 58 | .994 |
| Week 0 – Week 24 ITT | 0.133 | 1 | 57 | .717 |
| Week 12 – Week 24 ITT | 0.000 | 1 | 57 | .996 |
| Week 0 – Week 12 PP | 0.004 | 1 | 56 | .950 |
| Week 0 – Week 24 PP | 0.066 | 1 | 56 | .798 |
| Week 12 – Week 24 PP | 0.006 | 1 | 56 | .940 |

# ATTENTION AND VIGILANCE COMPASS CHOICE REACTION TIME

## Descriptive analysis

Table 2.1 Descriptive Statistics for CRT % Correct Score by product at Baseline (Week 0; Visit 2) and End of Intervention (Week 24; Visit 4) in the ITT Population (N=66)

| **Product % Correct** | | **N** | | **Mean** | **SEM** | **SD** | **Min** | **Quartiles** | | | |
| --- | --- | --- | --- | --- | --- | --- | --- | --- | --- | --- | --- |
|  |  | **Valid** | **Missing** |  |  |  |  | **Q1** | **Mdn** | **Q3** | **Max** |
| PT extract | Week 0 | 33 | 0 | 93.03 | 4.19 | 24.08 | 0.00 | 98.00 | 100.00 | 100.00 | 100.00 |
|  | Week 12 | 28 | 5 | 99.36 | 0.23 | 1.22 | 96.00 | 98.50 | 100.00 | 100.00 | 100.00 |
|  | Week 24 | 28 | 5 | 99.43 | 0.20 | 1.07 | 96.00 | 98.50 | 100.00 | 100.00 | 100.00 |
|  | Change Week 12 | 28 | 5 | 7.21 | 4.92 | 26.01 | -4.00 | 0.00 | 0.00 | 2.00 | 100.00 |
|  | Change Week 24 | 28 | 5 | 7.29 | 4.96 | 26.25 | -2.00 | 0.00 | 0.00 | 0.00 | 100.00 |
| Placebo | Week 0 | 33 | 0 | 92.97 | 4.14 | 23.79 | 0.00 | 98.00 | 100.00 | 100.00 | 100.00 |
|  | Week 12 | 32 | 1 | 96.31 | 3.11 | 17.61 | 0.00 | 98.00 | 100.00 | 100.00 | 100.00 |
|  | Week 24 | 31 | 2 | 96.90 | 2.70 | 15.05 | 16.00 | 100.00 | 100.00 | 100.00 | 100.00 |
|  | Change Week 12 | 32 | 1 | 3.56 | 5.43 | 30.74 | -100.00 | 0.00 | 0.00 | 2.00 | 100.00 |
|  | Change Week 24 | 31 | 2 | 4.39 | 5.26 | 29.30 | -84.00 | 0.00 | 0.00 | 2.00 | 100.00 |

Table 2.2 Summary Descriptive Statistics for CRT Correct RT (ms) by product at Baseline (Week 0; Visit 2) and End of Intervention (Week 24; Visit 4) in the ITT Population (N=66)

| **Product Correct RT** | | **N** | | **Mean** | **SEM** | **SD** | **Min** | **Quartiles** | | | |
| --- | --- | --- | --- | --- | --- | --- | --- | --- | --- | --- | --- |
|  |  | **Valid** | **Missing** |  |  |  |  | **Q1** | **Mdn** | **Q3** | **Max** |
| PT extract | Week 0 | 31 | 2 | 998.52 | 86.41 | 481.11 | 480.92 | 655.60 | 791.26 | 1334.08 | 2134.84 |
|  | Week 12 | 28 | 5 | 811.66 | 58.58 | 310.00 | 456.86 | 578.98 | 706.58 | 997.29 | 1680.98 |
|  | Week 24 | 28 | 5 | 862.87 | 71.68 | 379.27 | 496.30 | 578.60 | 798.71 | 982.00 | 2274.92 |
|  | Change Week 12 | 26 | 7 | -124.74 | 48.15 | 245.52 | -746.25 | -159.10 | -97.02 | 23.19 | 245.70 |
|  | Change Week 24 | 26 | 7 | -80.39 | 77.95 | 397.48 | -1236.23 | -166.73 | -29.89 | 75.46 | 926.00 |
| Placebo | Week 0 | 32 | 1 | 1211.10 | 241.38 | 1365.43 | 406.00 | 702.70 | 838.39 | 1150.51 | 8021.16 |
|  | Week 12 | 31 | 2 | 998.87 | 157.11 | 874.73 | 527.88 | 612.54 | 767.10 | 893.84 | 5342.46 |
|  | Week 24 | 31 | 2 | 1147.55 | 299.55 | 1667.85 | 481.00 | 624.42 | 704.54 | 960.31 | 9808.86 |
|  | Change Week 12 | 30 | 3 | -228.87 | 302.80 | 1658.50 | -7378.08 | -268.07 | -64.66 | 143.07 | 3965.48 |
|  | Change Week 24 | 30 | 3 | -66.74 | 408.26 | 2236.14 | -7409.02 | -259.21 | -53.26 | 84.62 | 8769.76 |

**Primary analysis**

Table 2.3 Mann-Whitney U test to Determine If There Were Between Product Differences In Change In (1) CRT % Correct and (2) CRT Correct RT From Baseline (Week 0) To Week 12, From Baseline (Week 0) To Week 24, and From Week 12 To Week 24 In The ITT (N=66) and PP (N=58) Populations.

| **Between Groups*** | **Total**  **N** | **Mann-Whitney**  **U** | **Standardized**  **Test Statistic** | **Asymptotic Sig.**  **(2-sided test)** | **Effect**  **Size** |
| --- | --- | --- | --- | --- | --- |
| **CRT % Correct** |  |  |  |  |  |
| Change Week 0 - Week 12 ITT | 60 | 414.500 | -.551 | .581 | 0.071 |
| Change Week 0 - Week 24 ITT | 59 | 343.500 | -1.533 | .125 | 0.200 |
| Change Week 12 - Week 24 ITT | 59 | 405.500 | -.494 | .621 | 0.064 |
| Change Week 0 - Week 12 PP | 58 | 375.000 | -.779 | .436 | 0.102 |
| Change Week 0 - Week 24 PP | 58 | 315.500 | -1.827 | .068 | 0.240 |
| Change Week 12 - Week 24 PP | 58 | 377.500 | -.761 | .447 | 0.100 |
| **CRT Correct RT** |  |  |  |  |  |
| Change Week 0 - Week 12 ITT | 56 | 354.000 | -.591 | .554 | 0.079 |
| Change Week 0 - Week 24 ITT | 56 | 411.000 | .345 | .730 | 0.046 |
| Change Week 12 - Week 24 ITT | 58 | 477.000 | .887 | .375 | 0.116 |
| Change Week 0 - Week 12 PP | 54 | 354.000 | -.173 | .863 | 0.024 |
| Change Week 0 - Week 24 PP | 55 | 411.000 | .573 | .567 | 0.077 |
| Change Week 12 - Week 24 PP | 57 | 477.000 | 1.133 | .257 | 0.150 |
| *Shapiro-Wilks Test of Normality p<0.05. Therefore Non-Parametric Mann-Whitney U Test was used. | | | | | |

Table 2.4 Related-Samples Wilcoxon Signed Ranks Tests to Determine If There Were Within Product Differences In Change In CRT % Correct From Baseline/Week 0 To Week 24 in the ITT (N=66) and PP (N=58) Populations.

| **Within Group*** | **Total**  **N** | **Standardized**  **Test Statistic** | **Asymptotic Sig.**  **(2-sided test)** | **Effect**  **Size** |
| --- | --- | --- | --- | --- |
| **CRT % Correct** |  |  |  |  |
| **Week 0 - Week 12** |  |  |  |  |
| PT extract ITT | 28 | -.917 | .359 | 0.123 |
| Placebo ITT | 32 | -1.501 | .133 | 0.188 |
| PT extract PP | 28 | -.917 | .359 | 0.123 |
| Placebo PP | 30 | -1.693 | .090 | 0.219 |
| **Week 0 - Week 24** |  |  |  |  |
| PT extract ITT | 28 | -.975 | .329 | 0.130 |
| Placebo ITT | 31 | -2.311 | .021 | 0.293 |
| PT extract PP | 28 | -.975 | .329 | 0.130 |
| Placebo PP | 30 | -2.946 | .003 | 0.380 |
| **Week 12 – Week 24** |  |  |  |  |
| PT extract ITT | 28 | -.247 | .805 | 0.033 |
| Placebo ITT | 31 | -.595 | .552 | 0.076 |
| PT extract PP | 28 | -.247 | .805 | 0.033 |
| Placebo PP | 30 | -1.165 | .244 | 0.150 |
| *Shapiro-Wilks Test of Normality p<0.05. Therefore Non-Parametric Wilcoxon Signed Ranks Tests was used. | | | |  |

Table 2.5 Related-Samples Wilcoxon Signed Ranks Tests to Determine If There Were Within Product Differences In Change In CRT Correct RT From Baseline/Week 0 To Week 24 in the ITT (N=66) and PP (N=58) Populations.

| **Within Group*** | **Total**  **N** | **Standardized**  **Test Statistic** | **Asymptotic Sig.**  **(2-sided test)** | **Effect**  **Size** |
| --- | --- | --- | --- | --- |
| **CRT Correct RT** |  |  |  |  |
| **Week 0 - Week 12** |  |  |  |  |
| PT extract ITT | 26 | 2.146 | .032 | 0.298 |
| Placebo ITT | 30 | .998 | .318 | 0.129 |
| PT extract PP | 26 | 2.146 | .032 | 0.298 |
| Placebo PP | 28 | 1.640 | .101 | 0.219 |
| **Week 0 - Week 24** |  |  |  |  |
| PT extract ITT | 26 | .952 | .341 | 0.132 |
| Placebo ITT | 30 | 1.059 | .289 | 0.137 |
| PT extract PP | 26 | .952 | .341 | 0.132 |
| Placebo PP | 29 | 1.395 | .163 | 0.183 |
| **Week 12 – Week 24** |  |  |  |  |
| PT extract ITT | 28 | -1.093 | .274 | 0.146 |
| Placebo ITT | 30 | .113 | .910 | 0.015 |
| PT extract PP | 28 | -1.093 | .274 | 0.146 |
| Placebo PP | 29 | .400 | .689 | 0.053 |
| *Shapiro-Wilks Test of Normality p<0.05. Therefore Non-Parametric Wilcoxon Signed Ranks Tests was used. | | | |  |

## Sensitivity Analysis

Table 2.6 Quade Nonparametric Analysis of Covariance Tests to Determine If There Were Within Product Differences in Change in (1) CRT % Correct and (2) CRT Correct RT, from Baseline/Week 0 to Week 24 While Controlling for Baseline in the ITT and PP Population

| **Population** | **F** | **DFH** | **DFE** | **P Value** |
| --- | --- | --- | --- | --- |
| **% Correct** |  |  |  |  |
| Week 0 – Week 12 ITT | 0.015 | 1 | 58 | .901 |
| Week 0 – Week 24 ITT | 1.686 | 1 | 57 | .199 |
| Week 12 – Week 24 ITT | 0.240 | 1 | 57 | .626 |
| Week 0 – Week 12 PP | 0.000 | 1 | 56 | .992 |
| Week 0 – Week 24 PP | 2.815 | 1 | 56 | .099 |
| Week 12 – Week 24 PP | 0.618 | 1 | 56 | .435 |
| **CRT Correct RT** |  |  |  |  |
| Week 0 – Week 12 ITT | 1.665 | 1 | 54 | .202 |
| Week 0 – Week 24 ITT | 0.009 | 1 | 54 | .925 |
| Week 12 – Week 24 ITT | 0.149 | 1 | 53 | .701 |
| Week 0 – Week 12 PP | 0.693 | 1 | 52 | .409 |
| Week 0 – Week 24 PP | 0.025 | 1 | 53 | .874 |
| Week 12 – Week 24 PP | 0.387 | 1 | 52 | .537 |

# ATTENTION AND VIGILANCE COMPASS DIGIT VIGILANCE

## Descriptive analysis

Table 3.1 Summary Descriptive Statistics for Digit Vigilance % Correct by product at Baseline (Week 0; Visit 2) and End of Intervention (Week 24; Visit 4) in the ITT Population (N=66)

| **Product Digit Vigilance % Correct** | | **N** | | **Mean** | **SEM** | **SD** | **Min** | **Quartiles** | | | |
| --- | --- | --- | --- | --- | --- | --- | --- | --- | --- | --- | --- |
|  |  | **Valid** | **Missing** |  |  |  |  | **Q1** | **Mdn** | **Q3** | **Max** |
| PT extract | Week 0 | 33 | 0 | 78.43 | 3.09 | 17.77 | 33.33 | 68.89 | 82.22 | 93.33 | 97.78 |
|  | Week 12 | 28 | 5 | 88.33 | 2.16 | 11.42 | 48.89 | 85.00 | 91.11 | 95.56 | 100.00 |
|  | Week 24 | 28 | 5 | 85.47 | 3.96 | 20.97 | 0.00 | 84.44 | 91.11 | 97.23 | 100.00 |
|  | Change Week 12 | 28 | 5 | 7.41 | 2.26 | 11.98 | -11.11 | 0.03 | 4.45 | 13.34 | 40.00 |
|  | Change Week 24 | 28 | 5 | 4.55 | 3.02 | 15.97 | -33.34 | -3.29 | 4.45 | 11.11 | 37.78 |
| Placebo | Week 0 | 33 | 0 | 79.63 | 4.05 | 23.27 | 0.00 | 77.78 | 84.44 | 93.33 | 100.00 |
|  | Week 12 | 32 | 1 | 89.47 | 1.47 | 8.33 | 68.89 | 84.44 | 92.22 | 97.77 | 100.00 |
|  | Week 24 | 31 | 2 | 84.51 | 4.25 | 23.65 | 0.00 | 84.44 | 91.11 | 97.73 | 100.00 |
|  | Change Week 12 | 32 | 1 | 9.78 | 3.87 | 21.91 | -8.89 | -2.22 | 3.36 | 13.34 | 88.89 |
|  | Change Week 24 | 31 | 2 | 4.76 | 6.05 | 33.68 | -80.00 | -4.34 | 2.22 | 8.89 | 93.33 |

Table 3.2 Summary Descriptive Statistics for Digit Vigilance Correct RT (ms) by product at Baseline (Week 0; Visit 2) and End of Intervention (Week 24; Visit 4) in the ITT Population (N=66)

| **Product Digit Vigilance Correct RT** | | **N** | | **Mean** | **SEM** | **SD** | **Min** | **Quartiles** | | | |
| --- | --- | --- | --- | --- | --- | --- | --- | --- | --- | --- | --- |
|  |  | **Valid** | **Missing** |  |  |  |  | **Q1** | **Mdn** | **Q3** | **Max** |
| PT extract | Week 0 | 33 | 0 | 493.73 | 7.60 | 43.67 | 390.09 | 459.02 | 502.43 | 525.43 | 585.73 |
|  | Week 12 | 28 | 5 | 488.24 | 7.12 | 37.69 | 419.22 | 458.65 | 484.38 | 518.69 | 563.69 |
|  | Week 24 | 28 | 5 | 473.82 | 18.67 | 98.77 | 0.00 | 464.43 | 486.33 | 511.43 | 571.84 |
|  | Change Week 12 | 28 | 5 | 1.67 | 4.62 | 24.47 | -59.82 | -16.06 | 6.22 | 17.66 | 42.81 |
|  | Change Week 24 | 28 | 5 | -12.75 | 21.76 | 115.12 | -585.73 | -14.70 | 5.19 | 28.64 | 52.40 |
| Placebo | Week 0 | 33 | 0 | 460.37 | 21.17 | 121.59 | 0.00 | 461.36 | 484.23 | 511.24 | 531.95 |
|  | Week 12 | 32 | 1 | 485.21 | 4.45 | 25.18 | 416.50 | 472.80 | 485.59 | 499.99 | 532.66 |
|  | Week 24 | 31 | 2 | 476.83 | 17.13 | 95.36 | 0.00 | 469.79 | 495.92 | 514.22 | 556.46 |
|  | Change Week 12 | 32 | 1 | 26.33 | 20.48 | 115.84 | -83.08 | -17.23 | 0.63 | 21.61 | 463.72 |
|  | Change Week 24 | 31 | 2 | 17.90 | 29.31 | 163.21 | -529.06 | -9.17 | 3.66 | 28.95 | 497.98 |

## Primary Analysis

Table 3.3 Mann-Whitney U test to Determine If There Were Between Product Differences In Change In (1) Digital Vigilance % Correct and (2) Digital Vigilance Correct RT From Baseline (Week 0) To Week 12, From Baseline (Week 0) To Week 24, and From Week 12 To Week 24 In The ITT (N=66) and PP (N=58) Populations.

| **Between Groups*** | **Total**  **N** | **Mann-Whitney**  **U** | **Standardized**  **Test Statistic** | **Asymptotic Sig.**  **(2-sided test)** | **Effect**  **Size** |
| --- | --- | --- | --- | --- | --- |
| **Digital Vigilance % Correct** |  |  |  |  |  |
| Change Week 0 - Week 12 ITT | 60 | 475.000 | .400 | .689 | 0.052 |
| Change Week 0 - Week 24 ITT | 59 | 451.500 | .266 | .790 | 0.035 |
| Change Week 12 - Week 24 ITT | 59 | 426.000 | -.122 | .903 | 0.016 |
| Change Week 0 - Week 12 PP | 58 | 438.500 | .288 | .773 | 0.038 |
| Change Week 0 - Week 24 PP | 58 | 427.500 | .117 | .907 | 0.015 |
| Change Week 12 - Week 24 PP | 58 | 419.000 | -.016 | .988 | 0.002 |
| **Digital Vigilance Correct RT** |  |  |  |  |  |
| Change Week 0 - Week 12 ITT | 60 | 443.000 | -.074 | .941 | 0.010 |
| Change Week 0 - Week 24 ITT | 59 | 426.000 | -.121 | .903 | 0.016 |
| Change Week 12 - Week 24 ITT | 59 | 393.000 | -.622 | .534 | 0.081 |
| Change Week 0 - Week 12 PP | 58 | 431.000 | .171 | .864 | 0.022 |
| Change Week 0 - Week 24 PP | 58 | 404.000 | -.249 | .803 | 0.033 |
| Change Week 12 - Week 24 PP | 58 | 367.000 | -.825 | .410 | 0.108 |
| *Shapiro-Wilks Test of Normality p<0.05. Therefore Non-Parametric Mann-Whitney U Test was used. | | | | | |

Table 3.4 Related-Samples Wilcoxon Signed Ranks Tests to Determine If There Were Within Product Differences In Change In Digital Vigilance % Correct From Baseline/Week 0 To Week 24 in the ITT (N=66) and PP (N=58) Populations.

| **Within Group*** | **Total**  **N** | **Standardized**  **Test Statistic** | **Asymptotic Sig.**  **(2-sided test)** | **Effect**  **Size** |
| --- | --- | --- | --- | --- |
| **Digital Vigilance % Correct** |  |  |  |  |
| **Week 0 - Week 12** |  |  |  |  |
| PT extract ITT | 28 | -2.897 | .004 | 0.387 |
| Placebo ITT | 32 | -2.492 | .013 | 0.312 |
| PT extract PP | 28 | -2.897 | .004 | 0.387 |
| Placebo PP | 30 | -2.533 | .011 | 0.327 |
| **Week 0 - Week 24** |  |  |  |  |
| PT extract ITT | 28 | -1.898 | .058 | 0.254 |
| Placebo ITT | 31 | -1.629 | .103 | 0.207 |
| PT extract PP | 28 | -1.898 | .058 | 0.254 |
| Placebo PP | 30 | -1.743 | .081 | 0.225 |
| **Week 12 – Week 24** |  |  |  |  |
| PT extract ITT | 28 | .613 | .540 | 0.082 |
| Placebo ITT | 31 | .432 | .666 | 0.055 |
| PT extract PP | 28 | .613 | .540 | 0.082 |
| Placebo PP | 30 | .525 | .600 | 0.068 |
| *Shapiro-Wilks Test of Normality p<0.05. Therefore Non-Parametric Wilcoxon Signed Ranks Tests was used. | | | |  |

Table 3.5 Related-Samples Wilcoxon Signed Ranks Tests to Determine If There Were Within Product Differences In Change In Digital Vigilance Correct RT From Baseline/Week 0 To Week 24 in the ITT (N=66) and PP (N=58) Populations.

| **Within Group*** | **Total**  **N** | **Standardized**  **Test Statistic** | **Asymptotic Sig.**  **(2-sided test)** | **Effect**  **Size** |
| --- | --- | --- | --- | --- |
| **Digital Vigilance Correct RT** |  |  |  |  |
| **Week 0 - Week 12** |  |  |  |  |
| PT extract ITT | 28 | -.592 | .554 | 0.079 |
| Placebo ITT | 32 | -.561 | .575 | 0.070 |
| PT extract PP | 28 | -.592 | .554 | 0.079 |
| Placebo PP | 30 | -.257 | .797 | 0.033 |
| **Week 0 - Week 24** |  |  |  |  |
| PT extract ITT | 28 | -1.139 | .255 | 0.152 |
| Placebo ITT | 31 | -1.254 | .210 | 0.159 |
| PT extract PP | 28 | -1.139 | .255 | 0.152 |
| Placebo PP | 30 | -1.409 | .159 | 0.182 |
| **Week 12 – Week 24** |  |  |  |  |
| PT extract ITT | 28 | -1.116 | .265 | 0.149 |
| Placebo ITT | 31 | -1.431 | .153 | 0.182 |
| PT extract PP | 28 | -1.116 | .265 | 0.149 |
| Placebo PP | 30 | -1.738 | .082 | 0.224 |
| *Shapiro-Wilks Test of Normality p<0.05. Therefore Non-Parametric Wilcoxon Signed Ranks Tests was used. | | | |  |

## Sensitivity Analysis

Table 3.6 Quade Nonparametric Analysis of Covariance Tests to Determine If There Were Within Product Differences in Change in (1) Digital Vigilance % Correct and (2) Digital Vigilance Correct RT, from Baseline/Week 0 to Week 24 While Controlling for Baseline in the ITT and PP Population

| **Population** | **F** | **DFH** | **DFE** | **P Value** |
| --- | --- | --- | --- | --- |
| **Digital Vigilance % Correct** |  |  |  |  |
| Week 0 – Week 12 ITT | 0.215 | 1 | 58 | .645 |
| Week 0 – Week 24 ITT | 0.051 | 1 | 57 | .822 |
| Week 12 – Week 24 ITT | 0.013 | 1 | 57 | .910 |
| Week 0 – Week 12 PP | 0.111 | 1 | 56 | .740 |
| Week 0 – Week 24 PP | 0.011 | 1 | 56 | .918 |
| Week 12 – Week 24 PP | 0.000 | 1 | 56 | .990 |
| **Digital Vigilance Correct RT** |  |  |  |  |
| Week 0 – Week 12 ITT | 0.004 | 1 | 58 | .950 |
| Week 0 – Week 24 ITT | 0.005 | 1 | 57 | .942 |
| Week 12 – Week 24 ITT | 0.373 | 1 | 57 | .544 |
| Week 0 – Week 12 PP | 0.138 | 1 | 56 | .712 |
| Week 0 – Week 24 PP | 0.026 | 1 | 56 | .872 |
| Week 12 – Week 24 PP | 0.651 | 1 | 56 | .423 |

# EXECUTIVE FUNCTION, ATTENTION AND VIGILANCE COMPASS STROOP SCORE

## Descriptive Analysis

Table 4.1 Summary Descriptive Statistics for Stroop % Correct by product at Baseline (Week 0; Visit 2) and End of Intervention (Week 24; Visit 4) in the ITT Population (N=66).

| **Product Stroop % Correct** | | **N** | | **Mean** | **SEM** | **SD** | **Min** | **Quartiles** | | | |
| --- | --- | --- | --- | --- | --- | --- | --- | --- | --- | --- | --- |
|  |  | **Valid** | **Missing** |  |  |  |  | **Q1** | **Mdn** | **Q3** | **Max** |
| PT extract | Week 0 | 33 | 0 | 85.91 | 3.82 | 21.93 | 18.33 | 76.66 | 96.67 | 100.00 | 100.00 |
|  | Week 12 | 28 | 5 | 88.51 | 4.37 | 23.11 | 11.67 | 95.00 | 98.33 | 100.00 | 100.00 |
|  | Week 24 | 28 | 5 | 86.85 | 3.95 | 20.88 | 50.00 | 65.00 | 98.33 | 100.00 | 100.00 |
|  | Change Week 12 | 28 | 5 | 0.83 | 4.10 | 21.70 | -86.66 | -1.67 | 0.00 | 1.67 | 50.00 |
|  | Change Week 24 | 28 | 5 | -0.83 | 3.92 | 20.75 | -48.33 | -1.67 | 0.00 | 3.33 | 50.00 |
| Placebo | Week 0 | 33 | 0 | 87.37 | 3.23 | 18.54 | 48.33 | 83.34 | 96.67 | 99.17 | 100.00 |
|  | Week 12 | 32 | 1 | 90.99 | 2.89 | 16.35 | 50.00 | 92.09 | 98.33 | 100.00 | 100.00 |
|  | Week 24 | 31 | 2 | 94.95 | 2.01 | 11.22 | 50.00 | 96.67 | 98.33 | 100.00 | 100.00 |
|  | Change Week 12 | 32 | 1 | 2.39 | 3.71 | 20.97 | -48.33 | -2.92 | 1.66 | 8.33 | 48.33 |
|  | Change Week 24 | 31 | 2 | 6.72 | 3.81 | 21.20 | -46.67 | -1.67 | 1.67 | 15.00 | 48.33 |

Table 4.2 Summary Descriptive Statistics for Stroop Overall RT by product at Baseline (Week 0; Visit 2) and End of Intervention (Week 24; Visit 4) in the ITT Population (N=66).

| **Product Stroop Overall RT** | | **N** | | **Mean** | **SEM** | **SD** | **Min** | **Quartiles** | | | |
| --- | --- | --- | --- | --- | --- | --- | --- | --- | --- | --- | --- |
|  |  | **Valid** | **Missing** |  |  |  |  | **Q1** | **Mdn** | **Q3** | **Max** |
| PT extract | Week 0 | 33 | 0 | 1368.64 | 117.00 | 672.10 | 816.22 | 1056.58 | 1195.75 | 1573.86 | 4772.50 |
|  | Week 12 | 28 | 5 | 1166.21 | 48.56 | 256.94 | 877.35 | 938.83 | 1108.09 | 1384.71 | 1745.85 |
|  | Week 24 | 28 | 5 | 1111.98 | 45.31 | 239.78 | 796.00 | 947.87 | 1074.40 | 1186.62 | 1982.90 |
|  | Change Week 12 | 28 | 5 | -59.48 | 30.98 | 163.91 | -387.02 | -175.96 | -101.62 | 76.25 | 332.83 |
|  | Change Week 24 | 28 | 5 | -113.71 | 36.31 | 192.16 | -590.50 | -226.90 | -107.71 | 33.90 | 316.25 |
| Placebo | Week 0 | 33 | 0 | 1460.65 | 64.00 | 367.62 | 902.50 | 1201.42 | 1454.38 | 1643.76 | 2557.68 |
|  | Week 12 | 32 | 1 | 1325.40 | 73.82 | 417.60 | 908.93 | 1054.92 | 1238.57 | 1388.23 | 2936.92 |
|  | Week 24 | 31 | 2 | 1352.06 | 84.04 | 467.92 | 915.65 | 1045.47 | 1180.03 | 1468.17 | 2756.40 |
|  | Change Week 12 | 32 | 1 | -150.78 | 71.02 | 401.76 | -1203.53 | -424.88 | -151.50 | -44.30 | 998.85 |
|  | Change Week 24 | 31 | 2 | -112.92 | 108.73 | 605.41 | -1412.73 | -449.87 | -140.62 | 42.49 | 1544.67 |

## Primary Analysis

Table 4.3 Mann-Whitney U test to Determine If There Were Between Product Differences in Change in Stroop % Correct from Baseline (Week 0) To Week 12, From Baseline (Week 0) To Week 24, and From Week 12 To Week 24 In the ITT (N=66) and PP (N=58) Populations.

| **Between Groups*** | **Total**  **N** | **Mann-Whitney**  **U** | **Standardized**  **Test Statistic** | **Asymptotic Sig.**  **(2-sided test)** | **Effect**  **Size** |
| --- | --- | --- | --- | --- | --- |
| **Stroop % Correct** |  |  |  |  |  |
| Change Week 0 - Week 12 ITT | 60 | 376.500 | -1.067 | .286 | 0.138 |
| Change Week 0 - Week 24 ITT | 59 | 329.000 | -1.605 | .109 | 0.209 |
| Change Week 12 - Week 24 ITT | 59 | 365.000 | -1.067 | .286 | 0.139 |
| Change Week 0 - Week 12 PP | 58 | 342.500 | -1.216 | .224 | 0.160 |
| Change Week 0 - Week 24 PP | 58 | 322.000 | -1.535 | .125 | 0.202 |
| Change Week 12 - Week 24 PP | 58 | 357.500 | -.991 | .322 | 0.130 |
| *Shapiro-Wilks Test of Normality p<0.05. Therefore Non-Parametric Mann-Whitney U Test was used. | | | | | |

*Table 4.4 Independent-Samples T-test to Determine If There Were Between Product Differences In Change In Stroop Overall RT From Baseline/Week 0 To Week 12 in the ITT (N=66) and PP (N=58) Populations.*

|  |  |  | **95% CI of Difference** | | **2 sided** | **Effect Size** |
| --- | --- | --- | --- | --- | --- | --- |
| **Between Groups **** | **t** | **df** | **Lower** | **Upper** | **P value** | **Cohen's d** |
| **Stroop Overall Reaction Time** |  |  |  |  |  |  |
| Change Week 0 - Week 12 ITT | -1.178 | 42.164 | -247.657 | 65.039 | .245 | .291 |
| Change Week 0 - Week 24 ITT | .007 | 36.560 | -231.584 | 233.160 | .995 | .002 |
| Change Week 12 - Week 24 ITT | .828 | 39.761 | -118.487 | 283.028 | .412 | .208 |
| Change Week 0 - Week 12 PP | -1.069 | 38.552 | -250.689 | 77.389 | .292 | .274 |
| Change Week 0 - Week 24 PP | -.037 | 34.972 | -243.991 | 235.206 | .971 | .010 |
| Change Week 12 - Week 24 PP | .805 | 37.900 | -124.653 | 289.169 | .426 | .206 |
| * Shapiro-Wilks Test of Normality p>0.05 and Levene’s Test of Equal Variance p>0.05. Therefore, Parametric Unpaired Test was used with equal variance assumed.  ** Shapiro-Wilks Test of Normality p>0.05 and Levene’s Test of Equal Variance p<0.05. Therefore, Parametric Unpaired Test was used with equal variance not assumed. | | | | | | |

Table 4.5 Related-Samples Wilcoxon Signed Ranks Tests to Determine If There Were Within Product Differences In Change In Stroop % Correct From Baseline/Week 0 To Week 24 in the ITT (N=66) and PP (N=58) Populations.

| **Within Group*** | **Total**  **N** | **Standardized**  **Test Statistic** | **Asymptotic Sig.**  **(2-sided test)** | **Effect**  **Size** |
| --- | --- | --- | --- | --- |
| **Stroop % Correct** |  |  |  |  |
| **Week 0 - Week 12** |  |  |  |  |
| PT extract ITT | 28 | -.223 | .824 | 0.030 |
| Placebo ITT | 32 | -1.203 | .229 | 0.150 |
| PT extract PP | 28 | -.223 | .824 | 0.030 |
| Placebo PP | 30 | -1.306 | .191 | 0.169 |
| **Week 0 - Week 24** |  |  |  |  |
| PT extract ITT | 28 | -.302 | .762 | 0.040 |
| Placebo ITT | 31 | -2.131 | .033 | 0.271 |
| PT extract PP | 28 | -.302 | .762 | 0.040 |
| Placebo PP | 30 | -2.011 | .044 | 0.260 |
| **Week 12 – Week 24** |  |  |  |  |
| PT extract ITT | 28 | .107 | .915 | 0.014 |
| Placebo ITT | 31 | -1.726 | .084 | 0.219 |
| PT extract PP | 28 | .107 | .915 | 0.014 |
| Placebo PP | 30 | -1.616 | .106 | 0.209 |
| *Shapiro-Wilks Test of Normality p<0.05. Therefore Non-Parametric Wilcoxon Signed Ranks Tests was used. | | | |  |

Table 4.6 Related-Samples Wilcoxon Signed Ranks Tests to Determine If There Were Within Product Differences In Change In Stroop Overall RT From Baseline/Week 0 To Week 24 in the ITT (N=66) and PP (N=58) Populations.

| **Within Group*** | **Total**  **N** | **Standardized**  **Test Statistic** | **Asymptotic Sig.**  **(2-sided test)** | **Effect**  **Size** |
| --- | --- | --- | --- | --- |
| **Stroop Overall RT** |  |  |  |  |
| **Week 0 - Week 12** |  |  |  |  |
| PT extract ITT | 28 | 1.981 | .048 | 0.265 |
| Placebo ITT | 32 | 2.655 | .008 | 0.332 |
| PT extract PP | 28 | 1.981 | .048 | 0.265 |
| Placebo PP | 30 | 2.478 | .013 | 0.320 |
| **Week 0 - Week 24** |  |  |  |  |
| PT extract ITT | 28 | 2.801 | .005 | 0.374 |
| Placebo ITT | 31 | 1.901 | .057 | 0.241 |
| PT extract PP | 28 | 2.801 | .005 | 0.374 |
| Placebo PP | 30 | 1.882 | .060 | 0.243 |
| **Week 12 – Week 24** |  |  |  |  |
| PT extract ITT | 28 | .683 | .495 | 0.091 |
| Placebo ITT | 31 | -.176 | .860 | 0.022 |
| PT extract PP | 28 | .683 | .495 | 0.091 |
| Placebo PP | 30 | -.154 | .877 | 0.020 |
| *Shapiro-Wilks Test of Normality p<0.05. Therefore Non-Parametric Wilcoxon Signed Ranks Tests was used. | | | |  |

## Sensitivity Analysis

Table 4.7 Analysis of Covariance Tests to Determine If There Were Within Product Differences in Change in Overall RT from Baseline/Week 0 to Week 12; Baseline to Week 24 and Week 12 to Week 24 While Controlling for Baseline in the ITT (N=66) and PP(N=58) Populations

| **Group** | **Df. Within Groups** | **Df. Between Groups** | **F** | **Sig.** | **Partial Eta Squared** |
| --- | --- | --- | --- | --- | --- |
| Week 0 – Week 12 ITT | 1 | 57 | .002 | .968 | .000 |
| Week 0 – Week 24 ITT | 1 | 56 | 3.615 | .062 | .061 |
| Week 12 – Week 24 ITT |  |  |  |  |  |
| Week 0 – Week 12 PP | 1 | 55 | .001 | .975 | .000 |
| Week 0 – Week 24 PP | 1 | 55 | 3.502 | 0.067 | .060 |
| Week 12 – Week 24 PP |  |  |  |  |  |

# EPISODIC MEMORY COMPASS PICTURE RECOGNITION SCORE

## Descriptive Analysis

Table 5.1 Summary Descriptive Statistics for Picture Recognition - % Correct by product at Baseline (Week 0; Visit 2) and End of Intervention (Week 24; Visit 4) in the ITT Population (N=66)

| **Product Picture Recognition - % Correct** | | **N** | | **Mean** | **SEM** | **SD** | **Min** | **Quartiles** | | | |
| --- | --- | --- | --- | --- | --- | --- | --- | --- | --- | --- | --- |
|  |  | **Valid** | **Missing** |  |  |  |  | **Q1** | **Mdn** | **Q3** | **Max** |
| PT extract | Week 0 | 33 | 0 | 91.92 | 4.11 | 23.63 | 0.00 | 96.67 | 100.00 | 100.00 | 100.00 |
|  | Week 12 | 28 | 5 | 99.05 | 0.29 | 1.53 | 96.67 | 96.67 | 100.00 | 100.00 | 100.00 |
|  | Week 24 | 28 | 5 | 99.17 | 0.28 | 1.47 | 96.67 | 97.50 | 100.00 | 100.00 | 100.00 |
|  | Change Week 12 | 28 | 5 | 8.33 | 4.88 | 25.83 | -3.33 | 0.00 | 0.00 | 3.33 | 100.00 |
|  | Change Week 24 | 28 | 5 | 8.45 | 4.86 | 25.72 | -3.33 | 0.00 | 0.00 | 3.33 | 100.00 |
| Placebo | Week 0 | 33 | 0 | 89.80 | 4.36 | 25.07 | 0.00 | 93.33 | 100.00 | 100.00 | 100.00 |
|  | Week 12 | 32 | 1 | 95.94 | 3.00 | 16.99 | 3.33 | 96.67 | 100.00 | 100.00 | 100.00 |
|  | Week 24 | 31 | 2 | 99.25 | 0.37 | 2.06 | 90.00 | 100.00 | 100.00 | 100.00 | 100.00 |
|  | Change Week 12 | 32 | 1 | 6.46 | 5.55 | 31.39 | -96.67 | 0.00 | 0.00 | 5.84 | 100.00 |
|  | Change Week 24 | 31 | 2 | 10.11 | 4.64 | 25.81 | -10.00 | 0.00 | 0.00 | 6.67 | 100.00 |

Table 5.2 Summary Descriptive Statistics for Picture Recognition - Overall RT by product at Baseline (Week 0; Visit 2) and End of Intervention (Week 24; Visit 4) in the ITT Population (N=66)

| **Product Picture Recognition - Overall RT** | | **N** | | **Mean** | **SEM** | **SD** | **Min** | **Quartiles** | | | |
| --- | --- | --- | --- | --- | --- | --- | --- | --- | --- | --- | --- |
|  |  | **Valid** | **Missing** |  |  |  |  | **Q1** | **Mdn** | **Q3** | **Max** |
| PT extract | Week 0 | 33 | 0 | 1470.82 | 115.37 | 662.74 | 777.10 | 1092.07 | 1347.13 | 1648.97 | 4498.47 |
|  | Week 12 | 28 | 5 | 1257.09 | 73.23 | 387.51 | 784.93 | 945.46 | 1202.72 | 1404.51 | 2360.13 |
|  | Week 24 | 28 | 5 | 1081.07 | 78.58 | 415.82 | 747.67 | 863.65 | 984.60 | 1172.90 | 2964.80 |
|  | Change Week 12 | 28 | 5 | -113.75 | 62.39 | 330.15 | -818.44 | -313.69 | -92.20 | 134.55 | 562.30 |
|  | Change Week 24 | 28 | 5 | -289.76 | 54.40 | 287.84 | -872.76 | -428.97 | -310.64 | -96.76 | 363.13 |
| Placebo | Week 0 | 33 | 0 | 1444.90 | 72.00 | 413.61 | 900.03 | 1141.90 | 1367.17 | 1717.32 | 2390.93 |
|  | Week 12 | 32 | 1 | 1246.29 | 51.45 | 291.02 | 811.60 | 1036.18 | 1184.74 | 1499.46 | 1799.77 |
|  | Week 24 | 31 | 2 | 1075.04 | 43.79 | 243.82 | 747.63 | 904.83 | 1012.73 | 1246.40 | 1821.73 |
|  | Change Week 12 | 32 | 1 | -213.18 | 68.40 | 386.90 | -1039.94 | -551.53 | -140.53 | 89.93 | 419.57 |
|  | Change Week 24 | 31 | 2 | -393.55 | 81.52 | 453.86 | -1574.66 | -725.97 | -360.24 | -137.97 | 590.70 |

## Primary Analysis

Table 5.3 Mann-Whitney U test to Determine If There Were Between Product Differences In Change In Picture Recognition % Correct, From Baseline (Week 0) To Week 12, From Baseline (Week 0) To Week 24, and From Week 12 To Week 24 In The ITT (N=66) and PP (N=58) Populations

| **Between Groups*** | **Total**  **N** | **Mann-Whitney**  **U** | **Standardized**  **Test Statistic** | **Asymptotic Sig.**  **(2-sided test)** | **Effect**  **Size** |
| --- | --- | --- | --- | --- | --- |
| **% Correct** |  |  |  |  |  |
| Change Week 0 - Week 12 ITT | 60 | 422.000 | -.401 | .688 | 0.052 |
| Change Week 0 - Week 24 ITT | 59 | 378.000 | -.921 | .357 | 0.120 |
| Change Week 12 - Week 24 ITT | 59 | 399.000 | -.625 | .532 | 0.081 |
| Change Week 0 - Week 12 PP | 58 | 389.000 | -.500 | .617 | 0.066 |
| Change Week 0 - Week 24 PP | 58 | 361.000 | -.990 | .322 | 0.130 |
| Change Week 12 - Week 24 PP | 58 | 384.500 | -.647 | .518 | 0.085 |
| *Shapiro-Wilks Test of Normality p<0.05. Therefore Non-Parametric Mann-Whitney U Test was used. | | | | | |

Table 5.4 Independent-Samples T-test to Determine If There Were Between Product Differences In Change In Picture Recognition Overall RT From Baseline (Week 0) To Week 12, and From Baseline (Week 0) To Week 24 In The ITT (N=66) and PP (N=58) Populations.

|  |  |  | **95% CI of Difference** | | **2 sided** | **Effect Size** |
| --- | --- | --- | --- | --- | --- | --- |
| **Between Groups *** | **t** | **df** | **Lower** | **Upper** | **P value** | **Cohen's d** |
| **Overall Reaction Time** |  |  |  |  |  |  |
| Change Week 0 - Week 12 ITT | -1.063 | 58 | -286.728 | 87.877 | .292 | .275 |
| Change Week 0 - Week 24 ITT | -1.036 | 57 | -304.400 | 96.832 | .305 | .270 |
| Change Week 12 - Week 24 ITT | .124 | 57 | -172.609 | 195.322 | .902 | .032 |
| Change Week 0 - Week 12 PP | -1.260 | 56 | -309.987 | 70.621 | .213 | .331 |
| Change Week 0 - Week 24 PP | -1.145 | 56 | -318.203 | 86.700 | .257 | .301 |
| Change Week 12 - Week 24 PP | .042 | 56 | -182.525 | 190.388 | .966 | .011 |
| * Shapiro-Wilks Test of Normality p>0.05 and Levene’s Test of Equal Variance p>0.05. Therefore, Parametric Unpaired Test was used with equal variance assumed.  ** Shapiro-Wilks Test of Normality p>0.05 and Levene’s Test of Equal Variance p<0.05. Therefore, Parametric Unpaired Test was used with equal variance not assumed. | | | | | | |

Table 5.5 Related-Samples Wilcoxon Signed Ranks Tests to Determine If There Were Within Product Differences In Change In Picture Recognition % Correct From Baseline/Week 0 To Week 24 in the ITT (N=66) and PP (N=58) Populations.

| **Within Group*** | **Total**  **N** | **Standardized**  **Test Statistic** | **Asymptotic Sig.**  **(2-sided test)** | **Effect**  **Size** |
| --- | --- | --- | --- | --- |
| **% Correct** |  |  |  |  |
| **Week 0 - Week 12** |  |  |  |  |
| PT extract ITT | 28 | -2.090 | .037 | 0.279 |
| Placebo ITT | 32 | -2.038 | .042 | 0.255 |
| PT extract PP | 28 | -2.038 | .042 | 0.272 |
| Placebo PP | 30 | -2.090 | .037 | 0.270 |
| **Week 0 - Week 24** |  |  |  |  |
| PT extract ITT | 28 | -2.213 | .027 | 0.296 |
| Placebo ITT | 31 | -2.738 | .006 | 0.348 |
| PT extract PP | 28 | -2.213 | .027 | 0.296 |
| Placebo PP | 30 | -2.738 | .006 | 0.353 |
| **Week 12 – Week 24** |  |  |  |  |
| PT extract ITT | 28 | -.302 | .763 | 0.040 |
| Placebo ITT | 31 | -1.387 | .166 | 0.176 |
| PT extract PP | 28 | -.302 | .763 | 0.040 |
| Placebo PP | 30 | -1.387 | .166 | 0.179 |
| *Shapiro-Wilks Test of Normality p<0.05. Therefore Non-Parametric Wilcoxon Signed Ranks Tests was used. | | | |  |

Table 5.6 Related-Samples Paired t- Tests to Determine If There Were Within Product Differences In Change In Picture Recognition Overall RT From Baseline/Week 0 To Week 24 in the ITT (N=66) and PP (N=58) Populations.

|  |  | **95% Confidence Interval**  **of the Difference** | |  |  |  | **Effect Size** |
| --- | --- | --- | --- | --- | --- | --- | --- |
| **Product** |  | **Lower** | **Upper** | **t** | **df** | **Two-Sided**  **p-Value** | **Cohen’s d** |
| **Overall Reaction Time** | |  |  |  |  |  |  |
| **Week 0 - Week 12** | |  |  |  |  |  |  |
| PT extract ITT | | -241.769 | 14.266 | -1.823 | 27 | 0.079 | .345 |
| Placebo ITT | | -352.671 | -73.683 | -3.117 | 31 | 0.004 | .551 |
| PT extract PP | | -241.769 | 14.266 | -1.823 | 27 | 0.079 | .345 |
| Placebo PP | | -378.492 | -88.377 | -3.291 | 29 | 0.003 | .601 |
| **Week 0 - Week 24** | |  |  |  |  |  |  |
| PT extract ITT | | -401.376 | -178.152 | -5.327 | 27 | <.001 | 1.007 |
| Placebo ITT | | -560.026 | -227.07 | -4.828 | 30 | <.001 | .867 |
| PT extract PP | | -401.376 | -178.152 | -5.327 | 27 | <.001 | 1.007 |
| Placebo PP | | -576.020 | -235.010 | -4.864 | 29 | <.001 | .888 |
| **Week 12 - Week 24** | |  |  |  |  |  |  |
| PT extract ITT | | -292.092 | -59.933 | -3.111 | 27 | 0.004 | .588 |
| Placebo ITT | | -309.189 | -20.122 | -2.327 | 30 | 0.027 | .418 |
| PT extract PP | | -292.092 | -59.933 | -3.111 | 27 | 0.004 | .588 |
| Placebo PP | | -320.906 | -23.256 | -2.365 | 29 | 0.025 | .432 |

Table 5.7 Quade Nonparametric Analysis of Covariance Tests to Determine If There Were Within Product Differences in Change in % Correct response, from Baseline/Week 0 to Week 24 While Controlling for Baseline in the ITT and PP Population

| **Population** | **F** | **DFH** | **DFE** | **P Value** |
| --- | --- | --- | --- | --- |
| **% Correct** |  |  |  |  |
| Week 0 – Week 12 ITT | 0.000 | 1 | 58 | .985 |
| Week 0 – Week 24 ITT | 1.298 | 1 | 57 | .259 |
| Week 12 – Week 24 ITT | 0.462 | 1 | 57 | .499 |
| Week 0 – Week 12 PP | 0.046 | 1 | 56 | .832 |
| Week 0 – Week 24 PP | 1.140 | 1 | 56 | .290 |
| Week 12 – Week 24 PP | 0.510 | 1 | 56 | .478 |

Table 5.8 Secondary Endpoint – Analysis of Covariance Tests to Determine If There Were Within Product Differences in Change in Overall RT from Baseline/Week 0 to Week 12; Baseline to Week 24 and Week 12 to Week 24 While Controlling for Baseline in the ITT (N=66) and PP(N=58) Populations

| **Group** | **Df. Within Groups** | **Df. Between Groups** | **F** | **Sig.** | **Partial Eta Squared** |
| --- | --- | --- | --- | --- | --- |
| **Overall RT** |  |  |  |  |  |
| Week 0 – Week 12 ITT | 1 | 57 | 0.463 | 0.499 | 0.008 |
| Week 0 – Week 24 ITT | 1 | 56 | 0.329 | 0.568 | 0.006 |
| Week 12 – Week 24 ITT | 1 | 56 | 0.037 | 0.849 | 0.001 |
| Week 0 – Week 12 PP | 1 | 55 | 0.697 | 0.407 | 0.013 |
| Week 0 – Week 24 PP | 1 | 55 | 0.459 | 0.501 | 0.008 |
| Week 12 – Week 24 PP | 1 | 55 | 0.012 | 0.913 | 0.000 |

# EPISODIC MEMORY COMPASS WORD RECOGNITION SCORE

**Descriptive analysis**

Table 6.1 Summary Descriptive Statistics for Word Recognition % Correct by product at Baseline (Week 0; Visit 2) and End of Intervention (Week 24; Visit 4) in the ITT Population (N=66)

| **Product Word Recognition % Correct** | | **N** | | **Mean** | **SEM** | **SD** | **Min** | **Quartiles** | | | |
| --- | --- | --- | --- | --- | --- | --- | --- | --- | --- | --- | --- |
|  |  | **Valid** | **Missing** |  |  |  |  | **Q1** | **Mdn** | **Q3** | **Max** |
| PT extract | Week 0 | 33 | 0 | 80.91 | 2.91 | 16.69 | 26.67 | 76.67 | 86.67 | 90.00 | 100.00 |
|  | Week 12 | 28 | 5 | 84.52 | 1.95 | 10.31 | 63.33 | 76.67 | 86.67 | 92.50 | 100.00 |
|  | Week 24 | 28 | 5 | 86.07 | 1.80 | 9.52 | 66.67 | 76.67 | 86.67 | 93.33 | 100.00 |
|  | Change Week 12 | 28 | 5 | 3.81 | 3.64 | 19.26 | -26.67 | -6.67 | 0.00 | 10.00 | 56.66 |
|  | Change Week 24 | 28 | 5 | 5.36 | 3.46 | 18.31 | -16.67 | -6.67 | 3.33 | 10.00 | 60.00 |
| Placebo | Week 0 | 33 | 0 | 81.41 | 3.26 | 18.73 | 3.33 | 80.00 | 86.67 | 90.00 | 100.00 |
|  | Week 12 | 32 | 1 | 84.58 | 2.84 | 16.06 | 10.00 | 80.00 | 86.67 | 93.33 | 100.00 |
|  | Week 24 | 31 | 2 | 87.31 | 1.30 | 7.22 | 73.33 | 80.00 | 90.00 | 93.33 | 100.00 |
|  | Change Week 12 | 32 | 1 | 2.60 | 4.59 | 25.96 | -80.00 | -6.67 | 1.67 | 10.00 | 90.00 |
|  | Change Week 24 | 31 | 2 | 5.38 | 3.94 | 21.96 | -16.67 | -6.66 | 0.00 | 10.00 | 96.67 |

**Correct RT**

Table 6.2 Summary Descriptive Statistics for Word Recognition Correct Reaction Time by product at Baseline (Week 0; Visit 2) and End of Intervention (Week 24; Visit 4) in the ITT Population (N=66)

| **Product Word Recognition Reaction Time** | | **N** | | **Mean** | **SEM** | **SD** | **Min** | **Quartiles** | | | |
| --- | --- | --- | --- | --- | --- | --- | --- | --- | --- | --- | --- |
|  |  | **Valid** | **Missing** |  |  |  |  | **Q1** | **Mdn** | **Q3** | **Max** |
| PT extract | Week 0 | 33 | 0 | 1439.08 | 79.64 | 457.49 | 795.58 | 1100.92 | 1267.04 | 1760.43 | 2412.41 |
|  | Week 12 | 28 | 5 | 1275.07 | 69.79 | 369.31 | 754.67 | 1019.43 | 1186.52 | 1466.18 | 2322.50 |
|  | Week 24 | 28 | 5 | 1335.67 | 73.72 | 390.08 | 788.93 | 1052.58 | 1209.17 | 1654.55 | 2319.14 |
|  | Change Week 12 | 28 | 5 | -131.43 | 69.11 | 365.69 | -855.61 | -349.57 | -78.62 | 45.85 | 652.36 |
|  | Change Week 24 | 28 | 5 | -70.83 | 71.68 | 379.28 | -1037.51 | -325.04 | -67.81 | 116.33 | 974.29 |
| Placebo | Week 0 | 33 | 0 | 1374.44 | 78.38 | 450.25 | 802.10 | 1063.68 | 1315.69 | 1566.95 | 3091.79 |
|  | Week 12 | 32 | 1 | 1191.30 | 47.69 | 269.76 | 735.92 | 1003.51 | 1128.61 | 1367.42 | 1753.29 |
|  | Week 24 | 31 | 2 | 1195.69 | 44.41 | 247.29 | 794.86 | 1037.32 | 1140.93 | 1307.64 | 1922.67 |
|  | Change Week 12 | 32 | 1 | -178.34 | 65.58 | 371.00 | -1467.90 | -276.89 | -137.61 | 54.97 | 340.49 |
|  | Change Week 24 | 31 | 2 | -174.25 | 74.56 | 415.16 | -1922.02 | -254.78 | -143.69 | 29.55 | 339.24 |

## Primary Analysis

Table 6.3 Mann-Whitney U test to Determine If There Were Between Product Differences In Change In (1) Word Recognition % Correct and (2) Word Recognition Correct RT From Baseline (Week 0) To Week 12, From Baseline (Week 0) To Week 24, and From Week 12 To Week 24 In The ITT (N=66) and PP (N=58) Populations.

| **Between Groups*** | **Total**  **N** | **Mann-Whitney**  **U** | **Standardized**  **Test Statistic** | **Asymptotic Sig.**  **(2-sided test)** | **Effect**  **Size** |
| --- | --- | --- | --- | --- | --- |
| **Word Recognition % Correct** |  |  |  |  |  |
| Change Week 0 - Week 12 ITT | 60 | 437.000 | -.164 | .870 | 0.021 |
| Change Week 0 - Week 24 ITT | 59 | 450.000 | .243 | .808 | 0.032 |
| Change Week 12 - Week 24 ITT | 59 | 467.500 | .512 | .609 | 0.067 |
| Change Week 0 - Week 12 PP | 58 | 405.500 | -.226 | .821 | 0.030 |
| Change Week 0 - Week 24 PP | 58 | 428.500 | .133 | .895 | 0.017 |
| Change Week 12 - Week 24 PP | 58 | 452.000 | .501 | .616 | 0.066 |
| **Word Recognition Correct RT** |  |  |  |  |  |
| Change Week 0 - Week 12 ITT | 60 | 482.000 | .504 | .614 | 0.065 |
| Change Week 0 - Week 24 ITT | 59 | 471.000 | .562 | .574 | 0.073 |
| Change Week 12 - Week 24 ITT | 59 | 450.000 | .243 | .808 | 0.032 |
| Change Week 0 - Week 12 PP | 58 | 470.000 | .778 | .437 | 0.102 |
| Change Week 0 - Week 24 PP | 58 | 459.000 | .607 | .544 | 0.080 |
| Change Week 12 - Week 24 PP | 58 | 428.000 | .124 | .901 | 0.016 |
| *Shapiro-Wilks Test of Normality p<0.05. Therefore Non-Parametric Mann-Whitney U Test was used. | | | | | |

Table 6.4 Related-Samples Wilcoxon Signed Ranks Tests to Determine If There Were Within Product Differences In Change In Word Recognition % Correct From Baseline/Week 0 To Week 24 in the ITT (N=66) and PP (N=58) Populations.

| **Within Group*** | **Total**  **N** | **Standardized**  **Test Statistic** | **Asymptotic Sig.**  **(2-sided test)** | **Effect**  **Size** |
| --- | --- | --- | --- | --- |
| **Word Recognition % Correct** |  |  |  |  |
| **Week 0 - Week 12** |  |  |  |  |
| PT extract ITT | 28 | -.513 | .608 | 0.069 |
| Placebo ITT | 32 | -.700 | .484 | 0.088 |
| PT extract PP | 28 | -.513 | .608 | 0.069 |
| Placebo PP | 30 | -.744 | .457 | 0.096 |
| **Week 0 - Week 24** |  |  |  |  |
| PT extract ITT | 28 | -1.053 | .292 | 0.141 |
| Placebo ITT | 31 | -.748 | .454 | 0.095 |
| PT extract PP | 28 | -1.053 | .292 | 0.141 |
| Placebo PP | 30 | -.913 | .361 | 0.118 |
| **Week 12 – Week 24** |  |  |  |  |
| PT extract ITT | 28 | -.993 | .321 | 0.133 |
| Placebo ITT | 31 | -.459 | .646 | 0.058 |
| PT extract PP | 28 | -.993 | .321 | 0.133 |
| Placebo PP | 30 | -.459 | .646 | 0.059 |
| *Shapiro-Wilks Test of Normality p<0.05. Therefore Non-Parametric Wilcoxon Signed Ranks Tests was used. | | | |  |

Table 6.5 Related-Samples Wilcoxon Signed Ranks Tests to Determine If There Were Within Product Differences In Change In Word Recognition Correct RT From Baseline/Week 0 To Week 24 in the ITT (N=66) and PP (N=58) Populations.

| **Within Group*** | **Total**  **N** | **Standardized**  **Test Statistic** | **Asymptotic Sig.**  **(2-sided test)** | **Effect**  **Size** |
| --- | --- | --- | --- | --- |
| **Word Recognition Correct RT** |  |  |  |  |
| **Week 0 - Week 12** |  |  |  |  |
| PT extract ITT | 28 | 1.958 | .050 | 0.262 |
| Placebo ITT | 32 | 2.487 | .013 | 0.311 |
| PT extract PP | 28 | 1.958 | .050 | 0.262 |
| Placebo PP | 30 | 2.643 | .008 | 0.341 |
| **Week 0 - Week 24** |  |  |  |  |
| PT extract ITT | 38 | 1.230 | .219 | 0.141 |
| Placebo ITT | 31 | 2.391 | .017 | 0.304 |
| PT extract PP | 28 | 1.230 | .219 | 0.164 |
| Placebo PP | 30 | 2.355 | .019 | 0.304 |
| **Week 12 – Week 24** |  |  |  |  |
| PT extract ITT | 28 | -.274 | .784 | 0.037 |
| Placebo ITT | 31 | -.592 | .554 | 0.075 |
| PT extract PP | 28 | -.592 | .554 | 0.079 |
| Placebo PP | 30 | -.483 | .629 | 0.062 |
| *Shapiro-Wilks Test of Normality p<0.05. Therefore Non-Parametric Wilcoxon Signed Ranks Tests was used. | | | |  |

## Sensitivity Analysis

Table 6.6 Quade Nonparametric Analysis of Covariance Tests to Determine If There Were Within Product Differences in Change in (1) Work Recognition % Correct and (2) Work Recognition Correct RT, from Baseline/Week 0 to Week 24 While Controlling for Baseline in the ITT and PP Population

| **Population** | **F** | **DFH** | **DFE** | **P Value** |
| --- | --- | --- | --- | --- |
| **Work Recognition % Correct** |  |  |  |  |
| Week 0 – Week 12 ITT | 0.276 | 1 | 58 | .601 |
| Week 0 – Week 24 ITT | 0.000 | 1 | 57 | .990 |
| Week 12 – Week 24 ITT | 0.286 | 1 | 57 | .595 |
| Week 0 – Week 12 PP | 0.332 | 1 | 56 | .567 |
| Week 0 – Week 24 PP | 0.010 | 1 | 56 | .921 |
| Week 12 – Week 24 PP | 0.271 | 1 | 56 | .604 |
| **Work Recognition Correct RT** | 0.515 | 1 | 58 | .476 |
| Week 0 – Week 12 ITT | 0.829 | 1 | 57 | .366 |
| Week 0 – Week 24 ITT | 0.062 | 1 | 57 | .804 |
| Week 12 – Week 24 ITT | 1.311 | 1 | 56 | .257 |
| Week 0 – Week 12 PP | 1.020 | 1 | 56 | .317 |
| Week 0 – Week 24 PP | 0.017 | 1 | 56 | .896 |
| Week 12 – Week 24 PP | 0.515 | 1 | 58 | .476 |

# EPISODIC MEMORY COMPASS WORD RECALL SCORE

## Descriptive Analysis

Table 7.1 Summary Descriptive Statistics for Word Recall – Immediate Score by product at Baseline (Week 0; Visit 2) and End of Intervention (Week 24; Visit 4) in the ITT Population (N=66)

| **Product Word Recall – Immediate Score** | | **N** | | **Mean** | **SEM** | **SD** | **Min** | **Quartiles** | | | |
| --- | --- | --- | --- | --- | --- | --- | --- | --- | --- | --- | --- |
|  |  | **Valid** | **Missing** |  |  |  |  | **Q1** | **Mdn** | **Q3** | **Max** |
| PT extract | Week 0 | 33 | 0 | 3.39 | 0.45 | 2.56 | 0.00 | 1.00 | 3.00 | 5.50 | 8.00 |
|  | Week 12 | 28 | 5 | 4.61 | 0.41 | 2.18 | 2.00 | 3.00 | 4.00 | 6.00 | 10.00 |
|  | Week 24 | 28 | 5 | 5.21 | 0.39 | 2.06 | 2.00 | 4.00 | 5.00 | 6.00 | 10.00 |
|  | Change Week 12 | 28 | 5 | 1.00 | 0.45 | 2.37 | -5.00 | -1.00 | 1.00 | 3.00 | 5.00 |
|  | Change Week 24 | 28 | 5 | 1.61 | 0.36 | 1.89 | -2.00 | 0.00 | 1.00 | 3.00 | 6.00 |
| Placebo | Week 0 | 33 | 0 | 3.73 | 0.34 | 1.96 | 0.00 | 2.00 | 3.00 | 5.00 | 8.00 |
|  | Week 12 | 32 | 1 | 4.53 | 0.40 | 2.26 | 0.00 | 3.00 | 5.00 | 6.00 | 8.00 |
|  | Week 24 | 31 | 2 | 5.06 | 0.34 | 1.91 | 1.00 | 4.00 | 5.00 | 6.00 | 10.00 |
|  | Change Week 12 | 32 | 1 | 0.72 | 0.49 | 2.77 | -6.00 | -1.75 | 2.00 | 2.00 | 6.00 |
|  | Change Week 24 | 31 | 2 | 1.19 | 0.49 | 2.73 | -4.00 | -1.00 | 1.00 | 4.00 | 6.00 |

Table 7.2 Summary Descriptive Statistics for Word Recall – Delayed Score by product at Baseline (Week 0; Visit 2) and End of Intervention (Week 24; Visit 4) in the ITT Population (N=66)

| **Product Word Recall – Delayed Score** | | **N** | | **Mean** | **SEM** | **SD** | **Min** | **Quartiles** | | | |
| --- | --- | --- | --- | --- | --- | --- | --- | --- | --- | --- | --- |
|  |  | **Valid** | **Missing** |  |  |  |  | **Q1** | **Mdn** | **Q3** | **Max** |
| PT extract | Week 0 | 33 | 0 | 3.36 | 0.36 | 2.07 | 0.00 | 2.00 | 3.00 | 4.50 | 7.00 |
|  | Week 12 | 28 | 5 | 4.46 | 0.47 | 2.50 | 0.00 | 2.25 | 5.00 | 6.00 | 9.00 |
|  | Week 24 | 28 | 5 | 4.61 | 0.54 | 2.86 | 0.00 | 2.00 | 4.50 | 6.75 | 10.00 |
|  | Change Week 12 | 28 | 5 | 1.07 | 0.39 | 2.09 | -4.00 | 0.00 | 1.00 | 2.75 | 6.00 |
|  | Change Week 24 | 28 | 5 | 1.21 | 0.44 | 2.32 | -3.00 | 0.00 | 1.00 | 3.00 | 6.00 |
| Placebo | Week 0 | 33 | 0 | 3.30 | 0.44 | 2.51 | 0.00 | 0.50 | 3.00 | 5.50 | 8.00 |
|  | Week 12 | 32 | 1 | 4.66 | 0.48 | 2.72 | 0.00 | 3.00 | 4.00 | 6.75 | 13.00 |
|  | Week 24 | 31 | 2 | 4.13 | 0.38 | 2.11 | 0.00 | 3.00 | 4.00 | 5.00 | 9.00 |
|  | Change Week 12 | 32 | 1 | 1.28 | 0.51 | 2.91 | -4.00 | 0.00 | 1.00 | 2.00 | 11.00 |
|  | Change Week 24 | 31 | 2 | 0.65 | 0.44 | 2.43 | -3.00 | -1.00 | 1.00 | 2.00 | 7.00 |

## Primary Analysis

Table 7.3 Independent-Samples T-test to Determine If There Were Between Product Differences In Change In (1) Immediate Score and (2) Delayed Score From Baseline (Week 0) To Week 12, From Baseline (Week 0) To Week 24, and From Week 12 To Week 24 In The ITT (N=66) and PP (N=58) Populations.

|  |  |  | **95% CI of Difference** | | **2 sided** | **Effect Size** |
| --- | --- | --- | --- | --- | --- | --- |
| **Between Groups** | **t** | **df** | **Lower** | **Upper** | **P value** | **Cohen's d** |
| **Immediate Score** |  |  |  |  |  |  |
| Change Week 0 - Week 12 ITT* | -.419 | 58 | -1.625 | 1.063 | .677 | .108 |
| Change Week 0 - Week 24 ITT* | -.670 | 57 | -1.650 | .822 | .506 | .175 |
| Change Week 12 - Week 24 ITT* | -.682 | 53.603 | -1.269 | .829 | .676 | .110 |
| Change Week 0 - Week 12 PP* | -.443 | 56 | -1.657 | 1.057 | .660 | .116 |
| Change Week 0 - Week 24 PP** | -.657 | 51.422 | -1.651 | .837 | .514 | .170 |
| Change Week 12 - Week 24 PP* | -.207 | 56 | -1.146 | .932 | .837 | .054 |
| **Delayed Score** |  |  |  |  |  |  |
| Change Week 0 - Week 12 ITT | .317 | 58 | -1.117 | 1.536 | .753 | .082 |
| Change Week 0 - Week 24 ITT | -.919 | 57 | -1.810 | .671 | .362 | .240 |
| Change Week 12 - Week 24 ITT | -1.502 | 57 | -1.914 | .274 | .139 | .392 |
| Change Week 0 - Week 12 PP* | .383 | 56 | -1.107 | 1.630 | .703 | .101 |
| Change Week 0 - Week 24 PP* | -.726 | 56 | -1.682 | .787 | .471 | .191 |
| Change Week 12 - Week 24 PP* | -1.308 | 56 | -1.797 | .377 | .196 | .344 |
| * Shapiro-Wilks Test of Normality p>0.05 and Levene’s Test of Equal Variance p>0.05. Therefore, Parametric Unpaired Test was used with equal variance assumed.  ** Shapiro-Wilks Test of Normality p>0.05 and Levene’s Test of Equal Variance p<0.05. Therefore, Parametric Unpaired Test was used with equal variance not assumed. | | | | | | |

Table 7.4 Related-Samples Paired t- Tests to Determine If There Were Within Product Differences In Change In Immediate Recall Score From Baseline/Week 0 To Week 24 in the ITT (N=66) and PP (N=58) Populations

|  |  | **95% Confidence Interval**  **of the Difference** | |  |  |  | **Effect Size** |
| --- | --- | --- | --- | --- | --- | --- | --- |
| **Product** |  | **Lower** | **Upper** | **t** | **df** | **Two-Sided**  **p-Value** | **Cohen’s d** |
| **Immediate Recall Score** | |  |  |  |  |  |  |
| **Week 0 - Week 12** | |  |  |  |  |  |  |
| PT extract ITT | | .080 | 1.920 | 2.230 | 27 | .034 | .421 |
| Placebo ITT | | -.281 | 1.719 | 1.466 | 31 | .153 | .259 |
| PT extract PP | | .080 | 1.920 | 2.230 | 27 | .034 | .421 |
| Placebo PP | | -.329 | 1.729 | 1.391 | 29 | .175 | .254 |
| **Week 0 - Week 24** | |  |  |  |  |  |  |
| PT extract ITT | | .873 | 2.341 | 4.494 | 27 | <.001 | .849 |
| Placebo ITT | | .194 | 2.193 | 2.438 | 30 | .021 | .438 |
| PT extract PP | | .873 | 2.341 | 4.494 | 27 | <.001 | .849 |
| Placebo PP | | .165 | 2.235 | 2.371 | 29 | .025 | .433 |
| **Week 12 - Week 24** | |  |  |  |  |  |  |
| PT extract ITT | | -.199 | 1.413 | 1.545 | 27 | .134 | .292 |
| Placebo ITT | | -.326 | 1.100 | 1.109 | 30 | .276 | .199 |
| PT extract PP | | 1.413 | 1.545 | 27 | .134 | .292 | 1.413 |
| Placebo PP | | -.199 | 1.199 | 1.464 | 29 | .154 | .267 |

[Location of file: W:\Atlantia Studies\4. Analysis and Close-Out Trials\AFCRO-142 MicroPhyt\1. ISF_TMF\ISF & TMF Documents\15. Statistical Analysis\e) Derived Analysis Datasets\AF142_WordRecall_Immeidate_Analysis_Output and AF142_WordRecall_Immeidate_Analysis_Output]

Table 7.5 Related-Samples Paired t- Tests to Determine If There Were Within Product Differences In Change In Delayed Recall Score From Baseline/Week 0 To Week 24 in the ITT (N=66) and PP (N=58) Populations

|  |  | **95% Confidence Interval**  **of the Difference** | |  |  |  | **Effect Size** |
| --- | --- | --- | --- | --- | --- | --- | --- |
| **Product** |  | **Lower** | **Upper** | **t** | **df** | **Two-Sided**  **p-Value** | **Cohen’s d** |
| **Delayed Score** | |  |  |  |  |  |  |
| **Week 0 - Week 12** | |  |  |  |  |  |  |
| PT extract ITT | | .261 | 1.882 | 2.714 | 27 | .011 | .513 |
| Placebo ITT | | .232 | 2.330 | 2.491 | 31 | .018 | .440 |
| PT extract PP | | .261 | 1.882 | 2.714 | 27 | .011 | .513 |
| Placebo PP | | .214 | 2.453 | 2.436 | 29 | .021 | .445 |
| **Week 0 - Week 24** | |  |  |  |  |  |  |
| PT extract ITT | | .317 | 2.112 | 2.775 | 27 | .010 | .525 |
| Placebo ITT | | -.246 | 1.536 | 1.478 | 30 | .150 | .266 |
| PT extract PP | | .317 | 2.112 | 2.775 | 27 | .010 | .525 |
| Placebo PP | | -.120 | 1.653 | 1.769 | 29 | .087 | .323 |
| **Week 12 - Week 24** | |  |  |  |  |  |  |
| PT extract ITT | | -.601 | .887 | .394 | 27 | .697 | .074 |
| Placebo ITT | | -1.500 | .145 | -1.682 | 30 | .103 | .302 |
| PT extract PP | | -.601 | .887 | .394 | 27 | .697 | .074 |
| Placebo PP | | -1.385 | .252 | -1.416 | 29 | .167 | .258 |

## Sensitivity Analysis

Table 7.6 Analysis of Covariance Tests to Determine If There Were Within Product Differences in Change in (1) Immediate and (2) Delayed Scores from Baseline/Week 0 to Week 12; Baseline to Week 24 and Week 12 to Week 24 While Controlling for Baseline in the ITT (N=66) and PP(N=58) Populations

| **Group** | **Df. Within Groups** | **Df. Between Groups** | **F** | **Sig.** | **Partial Eta Squared** |
| --- | --- | --- | --- | --- | --- |
| **Immediate Score** |  |  |  |  |  |
| Week 0 – Week 12 ITT | 1 | 57 | .069 | .794 | .001 |
| Week 0 – Week 24 ITT | 1 | 56 | .248 | .620 | .004 |
| Week 12 – Week 24 ITT | 1 | 56 | .189 | .665 | .003 |
| Week 0 – Week 12 PP | 1 | 55 | .033 | .856 | .001 |
| Week 0 – Week 24 PP | 1 | 55 | .191 | .664 | .003 |
| Week 12 – Week 24 PP | 1 | 55 | .048 | .827 | .001 |
| **Delayed Score** |  |  |  |  |  |
| Week 0 – Week 12 ITT | 1 | 57 | .112 | .739 | .002 |
| Week 0 – Week 24 ITT | 1 | 56 | .886 | .351 | .016 |
| Week 12 – Week 24 ITT | 1 | 56 | 2.252 | .139 | .039 |
| Week 0 – Week 12 PP | 1 | 55 | .266 | .608 | .005 |
| Week 0 – Week 24 PP | 1 | 55 | .519 | .474 | .009 |
| Week 12 – Week 24 PP | 1 | 55 | 1.710 | .196 | .030 |

# EXPLORATORY ENDPOINT – COHEN’S PERCEIVED STRESS SCALE

**Descriptive Analysis**

Table 8.1 Summary Descriptive Statistics for PSS Total Score by product at Baseline (Week 0; Visit 2) and End of Intervention (Week 24; Visit 4) in the ITT Population (N=66)

| **Product PSS Total Score** | | **N** | | **Mean** | **SEM** | **Min** | **Quartiles** | | | **Max** |
| --- | --- | --- | --- | --- | --- | --- | --- | --- | --- | --- |
|  |  | **Valid** | **Missing** |  |  |  | **Q1** | **Mdn** | **Q3** |  |
| Pt extract | Week 0 | 33 | 0 | 19.30 | .96 | 9.00 | 15.50 | 21.00 | 23.00 | 32.00 |
|  | Week 12 | 28 | 5 | 16.64 | 1.22 | 6.00 | 13.00 | 15.50 | 20.00 | 35.00 |
|  | Week 24 | 28 | 5 | 16.29 | 1.58 | 3.00 | 10.00 | 15.50 | 22.75 | 38.00 |
|  | Change Week 12 | 28 | 5 | -2.54 | 1.23 | -10.00 | -7.75 | -3.50 | 1.25 | 22.00 |
|  | Change Week 24 | 28 | 5 | -2.89 | 1.35 | -17.00 | -7.00 | -2.50 | 1.00 | 13.00 |
| Placebo | Week 0 | 33 | 0 | 17.27 | 1.03 | 6.00 | 13.00 | 17.00 | 21.50 | 28.00 |
|  | Week 12 | 32 | 1 | 15.84 | 1.15 | 5.00 | 10.25 | 16.50 | 20.00 | 31.00 |
|  | Week 24 | 31 | 2 | 15.77 | 1.19 | 1.00 | 11.00 | 17.00 | 20.00 | 32.00 |
|  | Change Week 12 | 32 | 1 | -1.56 | 1.10 | -14.00 | -6.00 | -3.00 | 2.75 | 11.00 |
|  | Change Week 24 | 31 | 2 | -1.45 | 1.22 | -17.00 | -6.00 | -2.00 | 4.00 | 15.00 |

**Primary Analysis**

Table 8.2 Independent-Samples T-test to Determine If There Were Between Product Differences In Change In PSS Total Score From Baseline (Week 0) To Week 12, and From Baseline (Week 0) To Week 24 In The ITT (N=66) and PP (N=58) Populations.

|  |  |  | **95% CI of Difference** | | **2 sided** | **Effect Size** |
| --- | --- | --- | --- | --- | --- | --- |
| **Between Groups *** | **t** | **df** | **Lower** | **Upper** | **P value** | **Cohen's d** |
| **PSS Total Score** |  |  |  |  |  |  |
| Change Week 0 - Week 12 ITT | .592 | 58 | -2.319 | 4.265 | .278 | .153 |
| Change Week 0 - Week 24 ITT | .794 | 57 | -2.196 | 5.078 | .431 | .207 |
| Change Week 12 - Week 24 ITT | .155 | 57 | -2.717 | 3.173 | .877 | .040 |
| Change Week 0 - Week 12 PP | .548 | 56 | -2.394 | 4.199 | .586 | .144 |
| Change Week 0 - Week 24 PP | .510 | 56 | -2.615 | 4.401 | .612 | .134 |
| Change Week 12 - Week 24 PP | -.006 | 56 | -2.962 | 2.943 | .995 | .002 |
| * Shapiro-Wilks Test of Normality p>0.05 and Levene’s Test of Equal Variance p>0.05. Therefore, Parametric Unpaired Test was used with equal variance assumed.  ** Shapiro-Wilks Test of Normality p>0.05 and Levene’s Test of Equal Variance p<0.05. Therefore, Parametric Unpaired Test was used with equal variance not assumed. | | | | | | |

Table 8.3 Related-Samples Paired t- Tests to Determine If There Were Within Product Differences In Change In PSS Score From Baseline/Week 0 To Week 24 in the ITT (N=66) and PP Populations (N=58).

|  |  | **95% Confidence Interval**  **of the Difference** | |  |  |  | **Effect Size** |
| --- | --- | --- | --- | --- | --- | --- | --- |
| **Product** |  | **Lower** | **Upper** | **t** | **df** | **Two-Sided**  **p-Value** | **Cohen’s d** |
| **PSS Total Score** | |  |  |  |  |  |  |
| **Week 0 - Week 12** | |  |  |  |  |  |  |
| PT extract ITT | | -5.056 | -.015 | -2.06 | 27.00 | .049 | .390 |
| Placebo ITT | | -3.807 | .682 | -1.42 | 31.00 | .166 | .251 |
| PT extract PP | | -5.056 | -.015 | -2.06 | 27 | .049 | .390 |
| Placebo PP | | -3.886 | .619 | -1.48 | 29 | .149 | .271 |
| **Week 0 - Week 24** | |  |  |  |  |  |  |
| PT extract ITT | | -5.664 | -.122 | -2.14 | 27.00 | .041 | .405 |
| Placebo ITT | | -3.946 | 1.043 | -1.19 | 30.00 | .244 | .213 |
| PT extract PP | | -5.664 | -.122 | -2.14 | 27 | .041 | .405 |
| Placebo PP | | -4.308 | .308 | -1.77 | 29 | .087 | .324 |
| **Week 12 - Week 24** | |  |  |  |  |  |  |
| PT extract ITT | | -2.675 | 1.960 | -.32 | 27.00 | .754 | .060 |
| Placebo ITT | | -2.081 | 1.823 | -.14 | 30.00 | .894 | .024 |
| PT extract PP | | -2.675 | 1.960 | -.32 | 27 | .754 | .060 |
| Placebo PP | | -2.324 | 1.591 | -.38 | 29 | .704 | .070 |

# EXPLORATORY ENDPOINT – BOND-LADER MOOD RATING SCALE

**Descriptive Analysis**

Table 9.1 Summary Descriptive Statistics for Bond-Lader Mood Rating Subscale of Alertness by product at Baseline (Week 0; Visit 2) and End of Intervention (Week 24; Visit 4) in the ITT Population (N=66)

| **Product Alertness** | | **N** | | **Mean** | **SEM** | **SD** | **Min** | **Quartiles** | | | **Max** |
| --- | --- | --- | --- | --- | --- | --- | --- | --- | --- | --- | --- |
|  |  | **Valid** | **Missing** |  |  |  |  | **Q1** | **Mdn** | **Q3** |  |
| Pt extract | Week 0 | 33 | 0 | 67.38 | 2.44 | 13.99 | 43.67 | 57.44 | 68.11 | 77.22 | 96.11 |
|  | Week 12 | 28 | 5 | 73.48 | 2.94 | 15.55 | 44.00 | 62.36 | 74.56 | 83.14 | 99.78 |
|  | Week 24 | 28 | 5 | 67.38 | 3.46 | 18.30 | 21.56 | 52.31 | 68.89 | 80.03 | 99.11 |
|  | Change Week 12 | 28 | 5 | 5.23 | 3.09 | 16.36 | -21.55 | -4.77 | 2.88 | 13.00 | 51.56 |
|  | Change Week 24 | 28 | 5 | -.87 | 2.80 | 14.84 | -37.22 | -8.23 | .73 | 8.01 | 39.11 |
| Placebo | Week 0 | 33 | 0 | 68.17 | 2.76 | 15.83 | 41.33 | 56.89 | 64.67 | 82.34 | 97.67 |
|  | Week 12 | 32 | 1 | 71.01 | 2.94 | 16.61 | 37.00 | 57.69 | 71.72 | 83.14 | 99.11 |
|  | Week 24 | 31 | 2 | 68.75 | 2.98 | 16.59 | 23.67 | 62.11 | 65.78 | 80.22 | 99.44 |
|  | Change Week 12 | 32 | 1 | 2.73 | 2.06 | 11.64 | -16.22 | -5.17 | 1.05 | 10.81 | 29.67 |
|  | Change Week 24 | 31 | 2 | .87 | 2.73 | 15.19 | -28.22 | -10.55 | .22 | 7.11 | 37.33 |

Table 9.2 Summary Descriptive Statistics for Bond-Lader Mood Rating Subscale of Contentment by product at Baseline (Week 0; Visit 2) and End of Intervention (Week 24; Visit 4) in the ITT Population (N=66)

| **Product Contentment** | | **N** | | **Mean** | **SEM** | **SD** | **Min** | **Quartiles** | | | **Max** |
| --- | --- | --- | --- | --- | --- | --- | --- | --- | --- | --- | --- |
|  |  | **Valid** | **Missing** |  |  |  |  | **Q1** | **Mdn** | **Q3** |  |
| Pt extract | Week 0 | 33 | 0 | 77.72 | 2.49 | 14.33 | 41.00 | 67.50 | 77.80 | 90.90 | 99.80 |
|  | Week 12 | 28 | 5 | 83.29 | 2.70 | 14.30 | 49.20 | 73.45 | 87.70 | 94.80 | 99.80 |
|  | Week 24 | 28 | 5 | 75.80 | 3.64 | 19.27 | 17.00 | 67.05 | 80.90 | 87.30 | 99.60 |
|  | Change Week 12 | 28 | 5 | 5.14 | 2.51 | 13.27 | -13.80 | -6.10 | 3.10 | 12.10 | 39.40 |
|  | Change Week 24 | 28 | 5 | -2.35 | 2.77 | 14.68 | -43.40 | -8.65 | -2.10 | 6.25 | 28.60 |
| Placebo | Week 0 | 33 | 0 | 78.18 | 2.27 | 13.03 | 41.80 | 71.40 | 77.40 | 87.50 | 99.60 |
|  | Week 12 | 32 | 1 | 77.99 | 2.96 | 16.74 | 25.40 | 66.80 | 79.70 | 89.60 | 99.80 |
|  | Week 24 | 31 | 2 | 75.33 | 2.93 | 16.32 | 15.40 | 68.00 | 75.00 | 87.00 | 99.80 |
|  | Change Week 12 | 32 | 1 | .20 | 2.27 | 12.87 | -37.60 | -5.30 | .10 | 4.15 | 41.80 |
|  | Change Week 24 | 31 | 2 | -2.17 | 2.44 | 13.60 | -26.40 | -10.60 | -2.00 | 6.00 | 31.80 |

Table 9.3 Summary Descriptive Statistics for Bond-Lader Mood Rating Subscale of Calmness by product at Baseline (Week 0; Visit 2) and End of Intervention (Week 24; Visit 4) in the ITT Population (N=66)

| **Product Calmness** | | **N** | | **Mean** | **SEM** | **SD** | **Min** | **Quartiles** | | | **Max** |
| --- | --- | --- | --- | --- | --- | --- | --- | --- | --- | --- | --- |
|  |  | **Valid** | **Missing** |  |  |  |  | **Q1** | **Mdn** | **Q3** |  |
| Pt extract | Week 0 | 33 | 0 | 68.56 | 3.06 | 17.60 | 33.00 | 52.75 | 71.00 | 80.50 | 97.00 |
|  | Week 12 | 28 | 5 | 79.16 | 3.36 | 17.80 | 44.50 | 58.88 | 87.25 | 94.13 | 99.50 |
|  | Week 24 | 28 | 5 | 73.07 | 3.63 | 19.22 | 23.00 | 56.25 | 76.75 | 90.38 | 99.50 |
|  | Change Week 12 | 28 | 5 | 8.05 | 3.12 | 16.49 | -27.50 | -1.87 | 7.00 | 17.75 | 46.00 |
|  | Change Week 24 | 28 | 5 | 1.96 | 2.56 | 13.56 | -30.50 | -7.00 | 2.50 | 11.63 | 26.00 |
| Placebo | Week 0 | 33 | 0 | 66.67 | 3.62 | 20.79 | 8.50 | 48.75 | 72.00 | 83.50 | 98.00 |
|  | Week 12 | 32 | 1 | 70.73 | 3.80 | 21.50 | 20.50 | 59.38 | 77.25 | 84.63 | 100.00 |
|  | Week 24 | 31 | 2 | 67.82 | 2.88 | 16.05 | 37.50 | 58.00 | 65.50 | 82.50 | 100.00 |
|  | Change Week 12 | 32 | 1 | 2.25 | 2.99 | 16.93 | -53.50 | -5.87 | 1.50 | 12.00 | 49.00 |
|  | Change Week 24 | 31 | 2 | -.29 | 2.89 | 16.10 | -31.00 | -13.00 | .50 | 7.50 | 33.50 |

**Primary Analysis**

Table 9.4 Mann-Whitney U test to Determine If There Were Between Product Differences In Change In Bond-Lader (1) Alertness, (2) Contentment, (3) Calmness From Baseline (Week 0) To Week 12, From Baseline (Week 0) To Week 24, and From Week 12 To Week 24 In The ITT (N=66) and PP (N=58) Populations.

| **Between Groups*** | **Total**  **N** | **Mann-Whitney**  **U** | **Standardized**  **Test Statistic** | **Asymptotic Sig.**  **(2-sided test)** | **Effect**  **Size** |
| --- | --- | --- | --- | --- | --- |
| **Bond-Lader Alertness** |  |  |  |  |  |
| Change Week 0 - Week 12 ITT | 60 | 472.000 | .356 | .722 | .178 |
| Change Week 0 - Week 24 ITT | 59 | 430.500 | -.053 | .958 | .116 |
| Change Week 12 - Week 24 ITT | 59 | 410.000 | -.364 | .716 | .223 |
| Change Week 0 - Week 12 PP | 58 | 442.000 | .342 | .732 | .172 |
| Change Week 0 - Week 24 PP | 58 | 421.500 | .023 | .981 | .106 |
| Change Week 12 - Week 24 PP | 58 | 389.000 | -.482 | .630 | .231 |
| **Bond-Lader Contentment** |  |  |  |  |  |
| Change Week 0 - Week 12 ITT | 60 | 538.000 | 1.334 | .182 | .379 |
| Change Week 0 - Week 24 ITT | 59 | 444.000 | .152 | .879 | .012 |
| Change Week 12 - Week 24 ITT | 59 | 401.000 | -.501 | .616 | .317 |
| Change Week 0 - Week 12 PP | 58 | 503.000 | 1.292 | .196 | .363 |
| Change Week 0 - Week 24 PP | 58 | 428.500 | .132 | .895 | .016 |
| Change Week 12 - Week 24 PP | 58 | 385.000 | -.545 | .586 | .321 |
| **Bond-Lader Calmness** |  |  |  |  |  |
| Change Week 0 - Week 12 ITT | 60 | 535.000 | 1.289 | .197 | .347 |
| Change Week 0 - Week 24 ITT | 59 | 481.000 | .714 | .475 | .151 |
| Change Week 12 - Week 24 ITT | 59 | 400.500 | -.509 | .611 | .228 |
| Change Week 0 - Week 12 PP | 58 | 517.000 | 1.510 | .131 | .398 |
| Change Week 0 - Week 24 PP | 58 | 476.000 | .872 | .383 | .189 |
| Change Week 12 - Week 24 PP | 58 | 382.000 | -.591 | .554 | .282 |
| *Shapiro-Wilks Test of Normality p<0.05. Therefore Non-Parametric Mann-Whitney U Test was used. | | | | | |

Table 9.5 Related-Samples Paired t-tests to Determine If There Were Within Product Differences In Within Change In Bond-Lader Alertness From Baseline/Week 0 To Week 24 in the ITT (N=66) and PP Populations (N=58).

|  |  | **95% Confidence Interval**  **of the Difference** | |  |  |  | **Effect Size** |
| --- | --- | --- | --- | --- | --- | --- | --- |
| **Product** |  | **Lower** | **Upper** | **t** | **df** | **Two-Sided**  **p-Value** | **Cohen’s d** |
| **Alertness** | |  |  |  |  |  |  |
| **Week 0 - Week 12** | |  |  |  |  |  |  |
| PT extract ITT | | -1.115 | 11.575 | 1.691 | 27 | .102 | .320 |
| Placebo ITT | | -1.469 | 6.926 | 1.326 | 31 | .195 | .234 |
| PT extract PP | | -1.115 | 11.575 | 1.691 | 27 | .102 | .320 |
| Placebo PP | | -1.579 | 7.170 | 1.307 | 29 | .201 | .239 |
| **Week 0 - Week 24** | |  |  |  |  |  |  |
| PT extract ITT | | -6.623 | 4.885 | -.310 | 27 | .759 | .059 |
| Placebo ITT | | -4.706 | 6.441 | .318 | 30 | .753 | .057 |
| PT extract PP | | -6.623 | 4.885 | -.310 | 27 | .759 | .059 |
| Placebo PP | | -5.024 | 6.505 | .263 | 29 | .795 | .048 |
| **Week 12 - Week 24** | |  |  |  |  |  |  |
| PT extract ITT | | -13.727 | 1.529 | -1.641 | 27 | .112 | .310 |
| Placebo ITT | | -7.741 | 3.276 | -.828 | 30 | .414 | .149 |
| PT extract PP | | -13.727 | 1.529 | -1.641 | 27 | .112 | .310 |
| Placebo PP | | -7.746 | 3.636 | -.739 | 29 | .466 | .135 |

Table 9.6 Related-Samples Wilcoxon Signed Rank to Determine If There Were Within Product Differences In Within Change In Bond-Lader Contentment From Baseline/Week 0 To Week 24 in the ITT (N=66) and PP Populations (N=58).

| **Within Group*** | **Total**  **N** | **Standardized**  **Test Statistic** | **Asymptotic Sig.**  **(2-sided test)** | **Effect**  **Size** |
| --- | --- | --- | --- | --- |
| **Contentment** |  |  |  |  |
| **Week 0 - Week 12** |  |  |  |  |
| PT extract ITT | 28 | -1.594 | .111 | 0.213 |
| Placebo ITT | 32 | .059 | .953 | 0.007 |
| PT extract PP | 28 | -1.594 | .111 | 0.213 |
| Placebo PP | 30 | -.076 | .940 | 0.010 |
| **Week 0 - Week 24** |  |  |  |  |
| PT extract ITT | 28 | .991 | .322 | 0.132 |
| Placebo ITT | 31 | 1.107 | .268 | 0.141 |
| PT extract PP | 28 | .991 | .322 | 0.132 |
| Placebo PP | 30 | 1.008 | .314 | 0.130 |
| **Week 12 – Week 24** |  |  |  |  |
| PT extract ITT | 28 | 2.323 | .020 | 0.310 |
| Placebo ITT | 31 | 1.224 | .221 | 0.155 |
| PT extract PP | 28 | 2.323 | .020 | 0.310 |
| Placebo PP | 30 | 1.114 | .265 | 0.144 |
| *Shapiro-Wilks Test of Normality p<0.05. Therefore Non-Parametric Wilcoxon Signed Ranks Tests was used. | | | |  |

Table 9.7 Related-Samples Wilcoxon Signed Rank to Determine If There Were Within Product Differences In Within Change In Bond-Lader Calmness From Baseline/Week 0 To Week 24 in the ITT (N=66) and PP Populations (N=58).

| **Within Group*** | **Total**  **N** | **Standardized**  **Test Statistic** | **Asymptotic Sig.**  **(2-sided test)** | **Effect**  **Size** |
| --- | --- | --- | --- | --- |
| **Calmness** |  |  |  |  |
| **Week 0 - Week 12** |  |  |  |  |
| PT extract ITT | 28 | -2.403 | .016 | 0.321 |
| Placebo ITT | 32 | -1.029 | .304 | 0.129 |
| PT extract PP | 28 | -2.403 | .016 | 0.321 |
| Placebo PP | 30 | -.648 | .517 | 0.084 |
| **Week 0 - Week 24** |  |  |  |  |
| PT extract ITT | 28 | -.901 | .368 | 0.120 |
| Placebo ITT | 31 | .010 | .992 | 0.001 |
| PT extract PP | 28 | -.901 | .368 | 0.120 |
| Placebo PP | 30 | .249 | .804 | 0.032 |
| **Week 12 – Week 24** |  |  |  |  |
| PT extract ITT | 28 | -1.594 | .111 | 0.213 |
| Placebo ITT | 31 | -.802 | .422 | 0.102 |
| PT extract PP | 28 | 1.594 | .111 | 0.213 |
| Placebo PP | 30 | .660 | .510 | 0.085 |
| *Shapiro-Wilks Test of Normality p<0.05. Therefore Non-Parametric Wilcoxon Signed Ranks Tests was used. | | | |  |

# EXPLORATORY ENDPOINT – LEEDS SLEEP EVALUATION QUESTIONNAIRE

**Descriptive Analysis**

Table 10.1 Summary Descriptive Statistics for Leeds Sleep Evaluation Questionnaire (LSEQ) Subscale of Getting to Sleep (GTS) by product at Baseline (Week 0; Visit 2) and End of Intervention (Week 24; Visit 4) in the ITT (N=66) Population

| **Product Getting to Sleep** | | **N** | | **Mean** | **SEM** | **SD** | **Min** | **Quartiles** | | | **Max** |
| --- | --- | --- | --- | --- | --- | --- | --- | --- | --- | --- | --- |
|  |  | **Valid** | **Missing** |  |  |  |  | **Q1** | **Mdn** | **Q3** |  |
| Pt Extract | Week 12 | 28 | 5 | 44.88 | 2.44 | 12.92 | 3.67 | 40.00 | 49.67 | 50.83 | 64.67 |
|  | Week 24 | 28 | 5 | 43.02 | 2.26 | 11.97 | 13.00 | 37.33 | 48.50 | 50.25 | 54.67 |
|  | Change Week 12 – Week 24 | 28 | 5 | -1.86 | 2.78 | 14.70 | -47.33 | -3.58 | .17 | 1.92 | 30.00 |
| Placebo | Week 12 | 32 | 1 | 47.53 | 2.47 | 13.95 | 11.33 | 37.75 | 49.33 | 52.67 | 88.67 |
|  | Week 24 | 31 | 2 | 47.05 | 2.55 | 14.18 | 4.33 | 47.67 | 49.00 | 50.67 | 81.00 |
|  | Change Week 12 – Week 24 | 31 | 2 | .11 | 3.25 | 18.10 | -57.00 | -3.33 | .67 | 9.67 | 32.00 |

Table 10.2 Summary Descriptive Statistics for Leeds Sleep Evaluation Questionnaire (LSEQ) Subscale of Quality of Sleep (QOS) by product at Baseline (Week 0; Visit 2) and End of Intervention (Week 24; Visit 4) in the ITT Population (N=66)

| **Product Quality of Sleep** | | **N** | | **Mean** | **SEM** | **SD** | **Min** | **Quartiles** | | | **Max** |
| --- | --- | --- | --- | --- | --- | --- | --- | --- | --- | --- | --- |
|  |  | **Valid** | **Missing** |  |  |  |  | **Q1** | **Mdn** | **Q3** |  |
| Pt Extract | Week 12 | 28 | 5 | 44.38 | 2.94 | 15.55 | .00 | 44.63 | 48.00 | 52.38 | 66.00 |
|  | Week 24 | 28 | 5 | 43.02 | 3.09 | 16.36 | .00 | 37.13 | 47.75 | 52.88 | 70.00 |
|  | Change Week 12 – Week 24 | 28 | 5 | -1.36 | 2.78 | 14.69 | -39.50 | -9.00 | 1.00 | 5.13 | 27.00 |
| Placebo | Week 12 | 32 | 1 | 50.97 | 2.78 | 15.71 | 14.00 | 42.75 | 50.75 | 61.13 | 77.00 |
|  | Week 24 | 31 | 2 | 48.63 | 2.85 | 15.85 | 8.50 | 43.00 | 49.50 | 56.50 | 84.50 |
|  | Change Week 12 – Week 24 | 31 | 2 | -2.44 | 2.79 | 15.54 | -29.50 | -14.00 | .00 | 7.00 | 42.50 |

Table 10.3 Summary Descriptive Statistics for Leeds Sleep Evaluation Questionnaire (LSEQ) Subscale of Awakening from Sleep (AFS) by product at Baseline (Week 0; Visit 2) and End of Intervention (Week 24; Visit 4) in the ITT Population (N=66)

| **Product Awakening from Sleep** | | **N** | | **Mean** | **SEM** | **SD** | **Min** | **Quartiles** | | | **Max** |
| --- | --- | --- | --- | --- | --- | --- | --- | --- | --- | --- | --- |
|  |  | **Valid** | **Missing** |  |  |  |  | **Q1** | **Mdn** | **Q3** |  |
| Pt Extract | Week 12 | 28 | 5 | 45.42 | 2.67 | 14.14 | 10.67 | 73.33 | 37.75 | 47.33 | 51.75 |
|  | Week 24 | 28 | 5 | 43.69 | 2.91 | 15.41 | 6.00 | 77.00 | 39.50 | 44.17 | 51.17 |
|  | Change Week 12 – Week 24 | 28 | 5 | -1.73 | 2.38 | 12.60 | -29.00 | 22.33 | -8.33 | -1.33 | 6.25 |
| Placebo | Week 12 | 32 | 1 | 45.51 | 1.97 | 11.14 | 16.33 | 66.67 | 38.08 | 46.83 | 53.92 |
|  | Week 24 | 31 | 2 | 42.51 | 2.59 | 14.40 | 5.67 | 69.33 | 35.33 | 44.00 | 52.00 |
|  | Change Week 12 – Week 24 | 31 | 2 | -3.44 | 1.92 | 10.69 | -19.33 | 32.67 | -9.00 | -4.67 | 2.67 |

Table 10.4 Summary Descriptive Statistics for Leeds Sleep Evaluation Questionnaire (LSEQ) Subscale Behaviour following Wakefulness (BFW) by product at Baseline (Week 0; Visit 2) and End of Intervention (Week 24; Visit 4) in the ITT Population (N=66)

| **Product Behaviour following Wakefulness** | | **N** | | **Mean** | **SEM** | **SD** | **Min** | **Quartiles** | | | **Max** |
| --- | --- | --- | --- | --- | --- | --- | --- | --- | --- | --- | --- |
|  |  | **Valid** | **Missing** |  |  |  |  | **Q1** | **Mdn** | **Q3** |  |
| Pt Extract | Week 12 | 28 | 5 | 41.46 | 2.46 | 13.04 | 5.50 | 35.63 | 43.50 | 49.63 | 73.50 |
|  | Week 24 | 28 | 5 | 40.96 | 3.01 | 15.94 | 7.50 | 29.88 | 39.50 | 51.38 | 76.00 |
|  | Change Week 12 – Week 24 | 28 | 5 | -.50 | 2.65 | 14.05 | -29.50 | -10.87 | -.75 | 11.50 | 27.00 |
| Placebo | Week 12 | 32 | 1 | 40.20 | 2.04 | 11.51 | 19.00 | 31.63 | 39.50 | 49.75 | 72.00 |
|  | Week 24 | 31 | 2 | 38.85 | 3.00 | 16.68 | 4.50 | 26.00 | 39.50 | 50.50 | 75.00 |
|  | Change Week 12 – Week 24 | 31 | 2 | -1.23 | 3.01 | 16.76 | -38.00 | -14.50 | -1.00 | 10.50 | 40.00 |

**Primary Analysis**

Table 10.5 Mann-Whitney U test to Determine If There Were Between Product Differences In Leeds Sleep Evaluation Questionnaire (LSEQ) (1) Getting to Sleep (GTS), (2) Quality of Sleep (QOS), (3) Awakening from Sleep (AFS), (4) Behaviour following Wakefulness (BFW) At Week 12 and at Week 24, and change From Week 12 To Week 24 In The ITT (N=66) and PP (N=58) Populations.

| **Between Groups*** | **Total**  **N** | **Mann-Whitney**  **U** | **Standardized**  **Test Statistic** | **Asymptotic Sig.**  **(2-sided test)** | **Effect**  **Size** |
| --- | --- | --- | --- | --- | --- |
| **LSEQ Getting to Sleep** |  |  |  |  |  |
| Week 12 ITT | 60 | 431.500 | -.245 | .807 | .197 |
| Week 24 ITT | 59 | 364.000 | -1.064 | .287 | .306 |
| Change Week 12 - Week 24 ITT | 59 | 394.500 | -.600 | .549 | .119 |
| Week 12 PP | 58 | 429.500 | .148 | .882 | .116 |
| Week 24 PP | 58 | 364.000 | -.873 | .383 | .274 |
| Change Week 12 - Week 24 PP | 58 | 373.500 | -.724 | .469 | .124 |
| **LSEQ Quality of Sleep** |  |  |  |  |  |
| Week 12 ITT | 60 | 363.000 | -1.260 | .208 | .422 |
| Week 24 ITT | 59 | 357.500 | -1.161 | .245 | .349 |
| Change Week 12 - Week 24 ITT | 59 | 459.500 | .387 | .699 | .071 |
| Week 12 PP | 58 | 349.000 | -1.105 | .269 | .376 |
| Week 24 PP | 58 | 354.500 | -1.019 | .308 | .330 |
| Change Week 12 - Week 24 PP | 58 | 434.500 | .226 | .821 | .033 |
| **LSEQ Awakening from Sleep** |  |  |  |  |  |
| Week 12 ITT | 60 | 438.500 | -.141 | .888 | .007 |
| Week 24 ITT | 59 | 459.500 | .387 | .699 | .080 |
| Change Week 12 - Week 24 ITT | 59 | 506.500 | 1.101 | .271 | .147 |
| Week 12 PP | 58 | 391.500 | -.444 | .657 | .067 |
| Week 24 PP | 58 | 435.000 | .233 | .815 | .046 |
| Change Week 12 - Week 24 PP | 58 | 483.500 | .988 | .323 | .131 |
| **LSEQ Behaviour following Wakefulness** |  |  |  |  |  |
| Week 12 ITT | 60 | 496.500 | .719 | .472 | .103 |
| Week 24 ITT | 59 | 456.500 | .342 | .733 | .129 |
| Change Week 12 - Week 24 ITT | 59 | 452.500 | .281 | .779 | .049 |
| Week 12 PP | 58 | 459.000 | .607 | .544 | .078 |
| Week 24 PP | 58 | 428.500 | .132 | .895 | .061 |
| Change Week 12 - Week 24 PP | 58 | 425.500 | .086 | .932 | .000 |
| *Shapiro-Wilks Test of Normality p<0.05. Therefore Non-Parametric Mann-Whitney U Test was used. | | | | | |

# EXPLORATORY ENDPOINT – hs-CRP

**Descriptive Analysis**

Table 11.1 Summary Descriptive Statistics for hs-CRP by product at Baseline (Week 0; Visit 2) and End of Intervention (Week 24; Visit 4) in the ITT Population (N=66)

| **Product hs-CRP** | | **N** | | **Mean** | **SEM** | **SD** | **Min** | **Quartiles** | | | |
| --- | --- | --- | --- | --- | --- | --- | --- | --- | --- | --- | --- |
|  |  | **Valid** | **Missing** |  |  |  |  | **Q1** | **Mdn** | **Q3** | **Max** |
| PT extract | Week 0 | 33 | 0 | 3.92 | 1.04 | 5.95 | 0.30 | 0.95 | 2.30 | 3.35 | 31.60 |
|  | Week 12 | 28 | 5 | 2.64 | 0.50 | 2.67 | 0.30 | 0.90 | 1.80 | 3.90 | 13.20 |
|  | Week 24 | 28 | 5 | 2.13 | 0.49 | 2.57 | 0.30 | 0.73 | 1.20 | 2.88 | 12.90 |
|  | Change Week 12 | 28 | 5 | -0.47 | 0.33 | 1.73 | -5.50 | -0.95 | -0.20 | 0.20 | 2.40 |
|  | Change Week 24 | 28 | 5 | -0.98 | 0.33 | 1.74 | -7.30 | -1.45 | -0.30 | 0.00 | 1.00 |
| Placebo | Week 0 | 33 | 0 | 3.43 | 1.02 | 5.87 | 0.20 | 0.60 | 0.90 | 2.95 | 25.00 |
|  | Week 12 | 32 | 1 | 2.43 | 0.43 | 2.46 | 0.30 | 0.73 | 1.40 | 3.18 | 10.80 |
|  | Week 24 | 31 | 2 | 3.26 | 0.85 | 4.74 | 0.20 | 0.60 | 1.50 | 2.60 | 22.30 |
|  | Change Week 12 | 32 | 1 | -1.08 | 0.92 | 5.23 | -19.00 | -0.45 | 0.10 | 0.30 | 10.00 |
|  | Change Week 24 | 31 | 2 | 0.36 | 0.90 | 5.01 | -13.90 | 0.00 | 0.20 | 0.80 | 20.90 |

**Primary Analysis**

Table 11.2 Mann-Whitney U test to Determine If There Were Between Product Differences In Change In hsCRP From Baseline (Week 0) To Week 12, From Baseline (Week 0) To Week 24, and From Week 12 To Week 24 In The ITT (N=66) and PP (N=58) Populations.

| **Between Groups*** | **Total**  **N** | **Mann-Whitney**  **U** | **Standardized**  **Test Statistic** | **Asymptotic Sig.**  **(2-sided test)** | **Effect**  **Size** |
| --- | --- | --- | --- | --- | --- |
| **hs-CRP** |  |  |  |  |  |
| Change Week 0 - Week 12 ITT | 60 | 387.00 | -.906 | .365 | 0.117 |
| Change Week 0 - Week 24 ITT | 59 | 209.500 | -3.414 | <.001 | 0.444 |
| Change Week 12 - Week 24 ITT | 59 | 277.000 | -2.387 | .017 | 0.311 |
| Change Week 0 - Week 12 PP | 58 | 331.000 | -1.388 | .165 | 0.182 |
| Change Week 0 - Week 24 PP | 58 | 181.500 | -3.718 | <.001 | 0.488 |
| Change Week 12 - Week 24 PP | 58 | 277.000 | -2.229 | .026 | 0.293 |
| *Shapiro-Wilks Test of Normality p<0.05. Therefore Non-Parametric Mann-Whitney U Test was used. | | | | | |

Table 11.3 Related-Samples Wilcoxon Signed Ranks Tests to Determine If There Were Within Product Differences In In Change In hsCRP From Baseline (Week 0) To Week 12, From Baseline (Week 0) To Week 24, and From Week 12 To Week 24 In The ITT (N=66) and PP (N=58) Populations

| **Within Group*** | **Total**  **N** | **Standardized**  **Test Statistic** | **Asymptotic Sig.**  **(2-sided test)** | **Effect**  **Size** |
| --- | --- | --- | --- | --- |
| **hs-CRP** |  |  |  |  |
| **Week 0 - Week 12** |  |  |  |  |
| PT extract ITT | 28 | 1.408 | .159 | 0.188 |
| Placebo ITT | 32 | .059 | .953 | 0.007 |
| PT extract PP | 28 | 1.408 | .159 | 0.188 |
| Placebo PP | 30 | -.597 | .550 | 0.077 |
| **Week 0 - Week 24** |  |  |  |  |
| PT extract ITT | 28 | 3.098 | .002 | 0.414 |
| Placebo ITT | 31 | -1.828 | .068 | 0.232 |
| PT extract PP | 28 | 3.098 | .002 | 0.414 |
| Placebo PP | 30 | -2.251 | .024 | 0.291 |
| **Week 12 – Week 24** |  |  |  |  |
| PT extract ITT | 28 | 2.924 | .003 | 0.391 |
| Placebo ITT | 31 | -.890 | .374 | 0.113 |
| PT extract PP | 28 | 2.924 | .003 | 0.391 |
| Placebo PP | 30 | -.626 | .531 | 0.081 |
| *Shapiro-Wilks Test of Normality p<0.05. Therefore Non-Parametric Wilcoxon Signed Ranks Tests was used. | | | |  |

# ADDITIONAL VARIABLE – INFLAMMATORY BLOOD BIOMARKERS – IL-6 (pg/mL)

Table 12.1 Summary Descriptive Statistics for IL-6 (pg/mL) by product at Baseline (Week 0; Visit 2) and End of Intervention (Week 24; Visit 4) in the ITT Population (N=66)

| **Product IL-6 (pg/mL)** | | **N** | | **Mean** | **SEM** | **SD** | **Min** | **Quartiles** | | | |
| --- | --- | --- | --- | --- | --- | --- | --- | --- | --- | --- | --- |
|  |  | **Valid** | **Missing** |  |  |  |  | **Q1** | **Mdn** | **Q3** | **Max** |
| PT extract | Week 0 | 33 | 0 | 57.60 | 4.35 | 24.96 | 32.56 | 39.74 | 48.57 | 66.64 | 131.23 |
|  | Week 12 | 28 | 5 | 40.61 | 2.84 | 15.05 | 25.69 | 28.19 | 34.45 | 51.71 | 71.30 |
|  | Week 24 | 28 | 5 | 47.02 | 4.46 | 23.62 | 24.21 | 33.10 | 39.07 | 55.07 | 118.02 |
|  | Change Week 12 | 28 | 5 | -11.44 | 2.42 | 12.82 | -36.84 | -18.81 | -12.66 | -5.62 | 28.64 |
|  | Change Week 24 | 28 | 5 | -5.03 | 3.10 | 16.38 | -46.90 | -12.19 | -4.93 | 0.17 | 33.09 |
| Placebo | Week 0 | 33 | 0 | 53.27 | 3.25 | 18.65 | 20.15 | 39.26 | 47.72 | 73.37 | 90.31 |
|  | Week 12 | 32 | 1 | 34.88 | 2.46 | 13.92 | 16.01 | 25.06 | 31.94 | 43.00 | 73.40 |
|  | Week 24 | 31 | 2 | 45.82 | 4.04 | 22.52 | 21.14 | 32.13 | 37.70 | 55.46 | 124.12 |
|  | Change Week 12 | 32 | 1 | -17.71 | 2.35 | 13.30 | -53.81 | -22.84 | -15.82 | -6.00 | -3.64 |
|  | Change Week 24 | 31 | 2 | -6.80 | 2.89 | 16.08 | -58.18 | -14.03 | -7.83 | 2.56 | 36.82 |

*Table 12.2 Independent-Samples T-test to Determine If There Were Between Product Differences In Change In IL-6 (pg/mL) From Baseline/Week 0 To Week 12 in the ITT (N=66) and PP (N=58) Populations.*

|  |  |  | **95% CI of Difference** | | **2 sided** | **Effect Size** |
| --- | --- | --- | --- | --- | --- | --- |
| **Between Groups*** | **t** | **df** | **Lower** | **Upper** | **P value** | **Cohen's d** |
| **IL-6 (pg/Ml)** |  |  |  |  |  |  |
| Change Week 0 - Week 12 ITT | -1.852 | 58.000 | -13.042 | 0.508 | 0.069 | .479 |
| Change Week 0 - Week 24 ITT | -0.419 | 57.000 | -10.240 | 6.697 | 0.677 | .109 |
| Change Week 12 - Week 24 ITT | 1.114 | 57.000 | -3.926 | 13.770 | 0.270 | .290 |
| Change Week 0 - Week 12 PP | -2.079 | 56.000 | -14.014 | -0.260 | 0.042 | .546 |
| Change Week 0 - Week 24 PP | -0.348 | 56.000 | -10.086 | 7.102 | 0.729 | .091 |
| Change Week 12 - Week 24 PP | 1.275 | 56.000 | -3.222 | 14.511 | 0.207 | .335 |
| * Shapiro-Wilks Test of Normality p>0.05 and Levene’s Test of Equal Variance p>0.05. Therefore, Parametric Unpaired Test was used with equal variance assumed.  ** Shapiro-Wilks Test of Normality p>0.05 and Levene’s Test of Equal Variance p<0.05. Therefore, Parametric Unpaired Test was used with equal variance not assumed. | | | | | | |

Table 12.3 Related-Samples Paired t- Tests to Determine If There Were Within Product Differences In Change In IL-6 (pg/mL) From Baseline/Week 0 To Week 24 in the ITT Population (N=66)

|  |  |  | **Paired Differences** | | **95% Confidence Interval**  **of the Difference** | |  |  |  | **Effect Size** |
| --- | --- | --- | --- | --- | --- | --- | --- | --- | --- | --- |
| **Product** |  |  | **Mean** | **SD** | **Lower** | **Upper** | **t** | **df** | **Two-Sided**  **p-Value** | **Cohen’s d** |
| Pt extract | Pair 1 | Week 0 – Week 12 | -11.442 | 12.818 | -16.412 | -6.471 | -4.723 | 27.000 | <.001 | .893 |
|  | Pair 2 | Week 0 – Week 24 | -5.033 | 16.382 | -11.385 | 1.319 | -1.626 | 27.000 | 0.116 | .307 |
|  | Pair 3 | Week 12 – Week 24 | 6.409 | 19.000 | -0.959 | 13.776 | 1.785 | 27.000 | 0.086 | .337 |
| Placebo | Pair 1 | Week 0 – Week 12 | -17.708 | 13.302 | -22.504 | -12.913 | -7.531 | 31.000 | <.001 | 1.331 |
|  | Pair 2 | Week 0 – Week 24 | -6.805 | 16.075 | -12.701 | -0.908 | -2.357 | 30.000 | 0.025 | .423 |
|  | Pair 3 | Week 12 – Week 24 | 11.331 | 14.860 | 5.880 | 16.782 | 4.245 | 30.000 | <.001 | .762 |

# ADDITIONAL VARIABLE – INFLAMMATORY BLOOD BIOMARKERS – TNF-α (pg/mL)

Table 13.1 Descriptive Statistics for TNF-α (pg/mL) by product at Baseline (Week 0; Visit 2) and End of Intervention (Week 24; Visit 4) in the ITT Population (N=66)

| **Product TNF-α (pg/mL)** | | **N** | | **Mean** | **SEM** | **SD** | **Min** | **Quartiles** | | | |
| --- | --- | --- | --- | --- | --- | --- | --- | --- | --- | --- | --- |
|  |  | **Valid** | **Missing** |  |  |  |  | **Q1** | **Mdn** | **Q3** | **Max** |
| PT extract | Week 0 | 33 | 0 | 51.88 | 6.84 | 39.27 | 19.64 | 25.74 | 41.56 | 54.82 | 159.97 |
|  | Week 12 | 28 | 5 | 27.54 | 5.36 | 28.38 | 7.58 | 13.97 | 17.69 | 30.40 | 140.01 |
|  | Week 24 | 28 | 5 | 17.03 | 3.12 | 16.52 | 6.65 | 8.34 | 10.78 | 17.59 | 73.95 |
|  | Change Week 12 | 28 | 5 | -18.72 | 1.87 | 9.90 | -48.54 | -23.94 | -16.52 | -11.59 | -3.35 |
|  | Change Week 24 | 28 | 5 | -29.23 | 3.82 | 20.23 | -90.92 | -36.61 | -26.07 | -15.04 | 0.01 |
| Placebo | Week 0 | 33 | 0 | 58.80 | 13.44 | 77.23 | 13.66 | 24.82 | 38.01 | 60.24 | 447.05 |
|  | Week 12 | 32 | 1 | 47.19 | 22.69 | 128.34 | 4.85 | 10.83 | 16.12 | 30.92 | 732.89 |
|  | Week 24 | 31 | 2 | 21.01 | 7.90 | 43.96 | 5.99 | 8.18 | 10.46 | 17.24 | 253.21 |
|  | Change Week 12 | 32 | 1 | -11.48 | 9.77 | 55.25 | -59.46 | -24.42 | -18.85 | -13.25 | 285.84 |
|  | Change Week 24 | 31 | 2 | -38.76 | 7.11 | 39.56 | -193.84 | -37.76 | -27.92 | -16.82 | 3.81 |

Table 13.2 Whitney U Tests to Determine If There Were Between Product Differences In Change In TNF-α (pg/mL) From Baseline (Week 0) To Week 12, From Baseline (Week 0) To Week 24, and From Week 12 To Week 24 In The ITT (N=66) and PP (N=58) Populations.

| **Between Groups*** | **Total**  **N** | **Mann-Whitney**  **U** | **Standardized**  **Test Statistic** | **Asymptotic Sig.**  **(2-sided test)** | **Effect**  **Size** |
| --- | --- | --- | --- | --- | --- |
| **TNF-α (pg/mL)** |  |  |  |  |  |
| Change Week 0 - Week 12 ITT | 60 | 497 | 0.726 | 0.468 | 0.094 |
| Change Week 0 - Week 24 ITT | 59 | 475 | 0.622 | 0.534 | 0.081 |
| Change Week 12 - Week 24 ITT | 59 | 410 | -0.364 | 0.716 | 0.047 |
| Change Week 0 - Week 12 PP | 58 | 485 | 1.011 | 0.312 | 0.133 |
| Change Week 0 - Week 24 PP | 58 | 468 | 0.747 | 0.455 | 0.098 |
| Change Week 12 - Week 24 PP | 58 | 394 | -0.405 | 0.686 | 0.053 |
| *Shapiro-Wilks Test of Normality p<0.05. Therefore Non-Parametric Mann-Whitney U Test was used. | | | | | |

Table 13.3 Related-Samples Wilcoxon Signed Ranks Tests to Determine If There Were Within Product Differences In Change TNF-α (pg/mL) From Baseline/Week 0 To Week 24 in the ITT (N=66) and PP (N=58) Populations.

| **Within Group*** | **Total**  **N** | **Standardized**  **Test Statistic** | **Asymptotic Sig.**  **(2-sided test)** | **Effect**  **Size** |
| --- | --- | --- | --- | --- |
| **TNF-α (pg/mL)** |  |  |  |  |
| **Week 0 - Week 12** |  |  |  |  |
| PT extract ITT | 28 | 4.623 | <.001 | 0.618 |
| Placebo ITT | 32 | 4.338 | <.001 | 0.542 |
| PT extract PP | 28 | 4.623 | <.001 | 0.618 |
| Placebo PP | 30 | 4.165 | <.001 | 0.538 |
| **Week 0 - Week 24** |  |  |  |  |
| PT extract ITT | 28 | 4.600 | <.001 | 0.615 |
| Placebo ITT | 31 | 4.840 | <.001 | 0.615 |
| PT extract PP | 28 | 4.600 | <.001 | 0.615 |
| Placebo PP | 30 | 4.762 | <.001 | 0.615 |
| **Week 12 – Week 24** |  |  |  |  |
| PT extract ITT | 28 | 4.053 | <.001 | 0.542 |
| Placebo ITT | 31 | 4.056 | <.001 | 0.515 |
| PT extract PP | 28 | 4.053 | <.001 | 0.542 |
| Placebo PP | 30 | 3.959 | <.001 | 0.511 |
| *Shapiro-Wilks Test of Normality p<0.05. Therefore Non-Parametric Wilcoxon Signed Ranks Tests was used. | | | |  |

# SAFETY ASSESSMENT

Table 14.1 Summary of all Events in the Safety population set (N=66)

| **Description** | **PT extract**  **N: E** | **Placebo**  **N: E** | **Total**  **N: E** |
| --- | --- | --- | --- |
| Total SAE | - | 1:4 | 1:4 |
| Total Product-Related SAEs | - | - | - |
| Total AE | 23:42 | 22:47 | 45:89 |
| Total Product-Related AEs | 8:10 | 2:2 | 10:12 |
| Total Events leading to IP discontinuation | - | - | - |
| Total Events leading to early termination of study | - | - | - |
| N=Participant Count; E=Event Count | | | |

Table 14.2 Frequency Table for Number of Adverse Events by MedDRA SOC and PT in PT extract group the Safety population set (N=33)

|  |  | **Related E (%)** | | | **Not related E (%)** | | | **Total E (%)** | | | **Total E(%)** |
| --- | --- | --- | --- | --- | --- | --- | --- | --- | --- | --- | --- |
| **SOC** | **PT** | **Mild** | **Moderate** | **Severe** | **Mild** | **Moderate** | **Severe** | **Mild** | **Moderate** | **Severe** |  |
| Cardiac disorders | Dizziness | 1(12.5) | - | - | - | - | - | 1(3.3) | 0(0.0) | - | 1(2.4) |
| Endocrine disorders | Glucose tolerance impaired | - | - | - | 4(18.2) | - | - | 4(13.3) | 0(0.0) | - | 4(9.5) |
| Gastrointestinal disorders | Change of bowel habit | 2(25.0) | 0(0.0) | - | - | - | - | 2(6.7) | 0(0.0) | - | 2(4.8) |
|  | Constipation | 0(0.0) | 1(50.0) | - | - | - | - | 0(0.0) | 1(8.3) | - | 1(2.4) |
|  | Nausea | 1(12.5) | 0(0.5) | - | - | - | - | 1(3.3) | 0(0.0) | - | 1(2.4) |
|  | Abdominal discomfort | - | - | - | 1(4.5) | - | - | 1(3.3) | 0(0.0) | - | 1(2.4) |
|  | Diarrhoea | 0(0.0) | 1(50.0) | - | 1(4.5) | - | - | 1(3.3) | 1(8.3) | - | 2(4.8) |
|  | Helicobacter gastritis | - | - | - | 1(4.5) | - | - | 1(3.3) | 0(0.0) | - | 1(2.4) |
|  | Vomiting | - | - | - | 1(4.5) | - | - | 1(3.3) | 0(0.0) | - | 1(2.4) |
| General disorders and administration site conditions | Influenza like illness | - | - | - | 1(4.5) | 2(20.0) | - | 1(3.3) | 2(16.7) | - | 3(7.1) |
| Immune system disorders | Immunisation reaction | - | - | - | 1(4.5) | - | - | 1(3.3) | 0(0.0) | - | 1(2.4) |
| Infections and infestations | Coronavirus infection | - | - | - | 1(4.5) | 1(10.0) | - | 1(3.3) | 1(8.3) | - | 2(4.8) |
|  | Nasopharyngitis | - | - | - | 2(9.1) | 0(0.0) | - | 2(6.7) | 0(0.0) | - | 2(4.8) |
|  | Sinusitis | - | - | - | 0(0.0) | 1(10.0) | - | 0(0.0) | 1(8.3) | - | 1(2.4) |
| Injury, poisoning and procedural complications | Burns first degree | - | - | - | 1(4.5) | 0(0.0) | - | 1(3.3) | 0(0.0) | - | 1(2.4) |
|  | Chemical burn | - | - | - | 1(4.5) | 0(0.0) | - | 1(3.3) | 0(0.0) | - | 1(2.4) |
|  | Contusion | - | - | - | 0(0.0) | 1(10.0) | - | 0(0.0) | 1(8.3) | - | 1(2.4) |
| Investigations | Aspartate aminotransferase increased | 1(12.5) | - | - | - | - | - | 1(3.3) | 0(0.0) | - | 1(2.4) |
|  | Blood pressure increased | - | - | - | 1(4.5) | - | - | 1(3.3) | 0(0.0) | - | 1(2.4) |
|  | Blood urea increased | - | - | - | 1(4.5) | - | - | 1(3.3) | 0(0.0) | - | 1(2.4) |
|  | Gamma-glutamyltransferase increased | 1(12.5) | - | - | 1(4.5) | - | - | 2(6.7) | 0(0.0) | - | 2(4.8) |
|  | Lymphocyte count decreased | - | - | - | 1(4.5) | - | - | 1(3.3) | 0(0.0) | - | 1(2.4) |
|  | Monocyte count increased | - | - | - | 1(4.5) | - | - | 1(3.3) | 0(0.0) | - | 1(2.4) |
| Metabolism and nutrition disorders | Increased appetite | 1(12.5) | - | - | - | - | - | 1(3.3) | 0(0.0) | - | 1(2.4) |
| Nervous system disorders | Headache | 1(12.5) | - | - | - | - | - | 1(3.3) | 0(0.0) | - | 1(2.4) |
| Musculoskeletal and connective tissue disorders | Back pain | - | - | - | 1(4.5) | - | - | 1(3.3) | 0(0.0) | - | 1(2.4) |
| Neoplasms benign, malignant and unspecified (incl cysts and polyps) | Nasal polyps | - | - | - | 0 | 1(10.0) | - | 0(0.0) | 1(8.3) | - | 1(2.4) |
| Renal and urinary disorders | Kidney infection | - | - | - | 0 | 1(10.0) | - | 0(0.0) | 1(8.3) | - | 1(2.4) |
| Respiratory, thoracic and mediastinal disorders | Cough | - | - | - | 1(4.5) | 0(0.0) | - | 1(3.3) | 0(0.0) | - | 1(2.4) |
|  | Lower respiratory tract infection | - | - | - | 0(0.0) | 3(30.0) | - | 0(0.0) | 3(25.0) | - | 3(7.1) |
| Total |  | 8(100.0) | 2(100.0) | - | 22(100.0) | 10(100.0) | - | 30(100.0) | 12(100.0) | - | 42(100.0) |
| E= Event Count The categories definite, probable and possibly related are collapsed into the related category. The categories unlikely to be related and not related are collapsed into the not related category. | | | | | | | | | | | |

Table 14.3 Frequency Table for Number of Adverse Events by MedDRA SOC and PT in Placebo group the Safety population set (N=66)

|  |  | **Related e (%)** | | | **Not related E (%)** | | | **Total E (%)** | | | **Total E(%)** |
| --- | --- | --- | --- | --- | --- | --- | --- | --- | --- | --- | --- |
| **SOC** | **PT** | **Mild** | **Moderate** | **Severe** | **Mild** | **Moderate** | **Severe** | **Mild** | **Moderate** | **Severe** |  |
| Ear and labyrinth disorders | Ear infection | - | - | - | 0(0.0) | 1(4.0) | - | 0(0.0) | 1(3.8) | - | 1(2.1) |
|  | Vertigo | - | - | - | 0(0.0) | 1(4.0) | - | 0(0.0) | 1(3.8) | - | 1(2.1) |
| Endocrine disorders | Glucose tolerance impaired | - | - | - | 1(5.0) | 2(8.0) | - | 1(4.8) | 2(7.7) | - | 3(6.4) |
| Gastrointestinal disorders | Abdominal discomfort | - | 1(100.0) | - | 0(0.0) | - | - | 0(0.0) | 1(3.8) | - | 1(2.1) |
|  | Diarrhoea | - | - | - | 0(0.0) | 1(4.0) | - | 0(0.0) | 1(3.8) | - | 1(2.1) |
|  | Dyspepsia | - | - | - | 1(5.0) | 0(0.0) | - | 1(4.8) | 0(0.0) | - | 1(2.1) |
|  | Gastrooesophageal reflux disease | - | - | - | 1(5.0) | 0(0.0) | - | 1(4.8) | 0(0.0) | - | 1(2.1) |
| General disorders and administration site conditions | Influenza like illness | - | - | - | 4(20.0) | 3(12.0) | - | 4(19.0) | 3(11.5) | - | 7(14.9) |
| Immune system disorders | Seasonal allergy | - | - | - | 0(0.0) | 1(4.0) | - | 0(0.0) | 1(3.8) | - | 1(2.1) |
| Infections and infestations | Coronavirus infection | - | - | - | 1(5.0) | 1(4.0) | - | 1(4.8) | 1(3.8) | - | 2(4.3) |
|  | Nasopharyngitis | - | - | - | 0(0.0) | 1(4.0) | - | 0(0.0) | 1(3.8) | - | 1(2.1) |
|  | Sinusitis | - | - | - | 0(0.0) | 2(8.0) | - | 0(0.0) | 2(7.7) | - | 2(4.3) |
|  | Tooth infection | - | - | - | 0(0.0) | 1(4.0) | - | 0(0.0) | 1(3.8) | - | 1(2.1) |
|  | Tracheitis | - | - | - | 0(0.0) | 1(4.0) | - | 0(0.0) | 1(3.8) | - | 1(2.1) |
| Injury, poisoning and procedural complications | Procedural pain | - | - | - | 1(5.0) | 0(0.0) | - | 1(4.8) | 0(0.0) | - | 1(2.1) |
|  | Road traffic accident | - | - | - | 0(0.0) | 1(4.0) | - | 0(0.0) | 1(3.8) | - | 1(2.1) |
| Investigations | Blood pressure increased | - | - | - | 2(10.0) | - | - | 2(9.5) | 0(0.0) | - | 2(4.3) |
|  | C-reactive protein increased | - | - | - | 2(10.0) | - | - | 2(9.5) | 0(0.0) | - | 2(4.3) |
|  | Gamma-glutamyltransferase increased | 1(100.0) | - | - | 1(5.0) | - | - | 2(9.5) | 0(0.0) | - | 2(4.3) |
|  | Heart rate increased | - | - | - | 1(5.0) | - | - | 1(4.8) | 0(0.0) | - | 1(2.1) |
|  | Red blood cell count increased | - | - | - | 1(5.0) | - | - | 1(4.8) | 0(0.0) | - | 1(2.1) |
| Metabolism and nutrition disorders | Hypercholesterolaemia | - | - | - | 1(5.0) | - | - | 1(4.8) | 0(0.0) | - | 1(2.1) |
|  | Vitamin B12 deficiency | - | - | - | 1(5.0) | - | - | 1(4.8) | 0(0.0) | - | 1(2.1) |
|  | Vitamin D deficiency | - | - | - | 1(5.0) | - | - | 1(4.8) | 0(0.0) | - | 1(2.1) |
| Renal and urinary disorders | Kidney infection | - | - | - | 0(0.0) | 1(4.0) | - | 0(0.0) | 1(3.8) | - | 1(2.1) |
|  | Urinary tract infection | - | - | - | 0(0.0) | 1(4.0) | - | 0(0.0) | 1(3.8) | - | 1(2.1) |
| Respiratory, thoracic and mediastinal disorders | Lower respiratory tract infection | - | - | - | 0(0.0) | 6(24.0) | - | 0(0.0) | 6(23.1) | - | 6(12.8) |
| Surgical and medical procedures | Uterine dilation and curettage | - | - | - | 1(5.0) | - | - | 1(4.8) | 0(0.0) | - | 1(2.1) |
| Vascular disorders | Peripheral coldness | - | - | - | 1(5.0) | 1(4.0) | - | 0(0.0) | 1(3.8) | - | 1(2.1) |
| Total | | 1(100.0) | 1(100.0) | - | 20(100.0) | 25(100.0) | - | 21(100.0) | 26(100.0) | - | 47(100.0) |

E= Event Count The categories definite, probable and possibly related are collapsed into the related category. The categories unlikely to be related and not related are collapsed into the not related category.

Table 14.4 Vital Signs – Summary Descriptive Statistics for Systolic Blood Pressure (SBP) by Product from Baseline (Week 0; Visit 2) to End of Intervention (Week 24; Visit 4) in the Safety Population (N=66)

| **Product SBP** | | **N** | | **Mean** | **SEM** | **SD** | **Min** | **Quartiles** | | | |
| --- | --- | --- | --- | --- | --- | --- | --- | --- | --- | --- | --- |
|  |  | **Valid** | **Missing** |  |  |  |  | **Q1** | **Mdn** | **Q3** | **Max** |
| PT extract | Week 0 | 33 | 0 | 131.36 | 2.15 | 12.34 | 101.00 | 125.50 | 132.00 | 138.00 | 160.00 |
|  | Week 12 | 28 | 5 | 132.21 | 2.01 | 10.61 | 112.00 | 125.00 | 130.00 | 138.00 | 153.00 |
|  | Week 24 | 28 | 5 | 135.89 | 2.48 | 13.14 | 121.00 | 125.25 | 134.00 | 141.50 | 171.00 |
|  | Change Week 12 | 28 | 5 | -0.46 | 1.81 | 9.59 | -26.00 | -6.75 | 0.50 | 5.00 | 16.00 |
|  | Change Week 24 | 28 | 5 | 3.21 | 2.15 | 11.40 | -15.00 | -2.50 | 2.50 | 9.75 | 37.00 |
| Placebo | Week 0 | 33 | 0 | 127.30 | 1.90 | 10.90 | 109.00 | 120.50 | 128.00 | 137.00 | 145.00 |
|  | Week 12 | 32 | 1 | 129.53 | 2.09 | 11.81 | 103.00 | 121.00 | 130.50 | 136.50 | 153.00 |
|  | Week 24 | 31 | 2 | 130.77 | 2.29 | 12.77 | 106.00 | 120.00 | 131.00 | 142.00 | 159.00 |
|  | Change Week 12 | 32 | 1 | 2.28 | 1.73 | 9.81 | -21.00 | -4.00 | 1.00 | 9.75 | 22.00 |
|  | Change Week 24 | 31 | 2 | 3.65 | 1.75 | 9.72 | -11.00 | -3.00 | 2.00 | 9.00 | 29.00 |

Table 14.5 Vital Signs- Summary Descriptive Statistics for Diastolic Blood Pressure (DBP) by Product from Baseline (Week 0; Visit 2) to End of Intervention (Week 24; Visit 4) in the Safety Population (N=66)

| **Product DBP** | | **N** | | **Mean** | **SEM** | **SD** | **Min** | **Quartiles** | | | |
| --- | --- | --- | --- | --- | --- | --- | --- | --- | --- | --- | --- |
|  |  | **Valid** | **Missing** |  |  |  |  | **Q1** | **Mdn** | **Q3** | **Max** |
| PT extract | Week 0 | 33 | 0 | 78.24 | 1.45 | 8.34 | 60.00 | 73.00 | 79.00 | 84.00 | 96.00 |
|  | Week 12 | 28 | 5 | 77.61 | 1.75 | 9.23 | 60.00 | 71.00 | 77.00 | 84.75 | 96.00 |
|  | Week 24 | 28 | 5 | 80.14 | 1.58 | 8.37 | 63.00 | 74.25 | 81.50 | 88.00 | 96.00 |
|  | Change Week 12 | 28 | 5 | -1.82 | 1.19 | 6.27 | -13.00 | -6.00 | -2.00 | 2.00 | 9.00 |
|  | Change Week 24 | 28 | 5 | 0.71 | 1.18 | 6.26 | -15.00 | -3.75 | 1.00 | 4.00 | 13.00 |
| Placebo | Week 0 | 33 | 0 | 77.91 | 1.53 | 8.77 | 60.00 | 74.00 | 80.00 | 83.00 | 97.00 |
|  | Week 12 | 32 | 1 | 79.91 | 1.47 | 8.30 | 55.00 | 73.25 | 82.00 | 86.00 | 93.00 |
|  | Week 24 | 31 | 2 | 79.87 | 1.45 | 8.07 | 60.00 | 72.00 | 81.00 | 84.00 | 101.00 |
|  | Change Week 12 | 32 | 1 | 1.44 | 1.82 | 10.29 | -13.00 | -7.00 | -0.50 | 6.50 | 29.00 |
|  | Change Week 24 | 31 | 2 | 1.32 | 1.54 | 8.60 | -15.00 | -4.00 | 1.00 | 6.00 | 21.00 |

Table 14.6 Vital Signs – Summary Descriptive Statistics for Heart Rate by Product from Baseline (Week 0; Visit 2) to End of Intervention (Week 24; Visit 4) in the Safety Population (N=66)

| **Product Heart Rate** | | **N** | | **Mean** | **SEM** | **SD** | **Min** | **Quartiles** | | | |
| --- | --- | --- | --- | --- | --- | --- | --- | --- | --- | --- | --- |
|  |  | **Valid** | **Missing** |  |  |  |  | **Q1** | **Mdn** | **Q3** | **Max** |
| PT extract | Week 0 | 33 | 0 | 73.48 | 1.70 | 9.77 | 55.00 | 66.00 | 73.00 | 82.00 | 91.00 |
|  | Week 12 | 28 | 5 | 70.25 | 1.25 | 6.60 | 58.00 | 66.00 | 70.50 | 75.50 | 82.00 |
|  | Week 24 | 28 | 5 | 70.64 | 1.77 | 9.36 | 52.00 | 66.25 | 70.00 | 78.50 | 89.00 |
|  | Change Week 12 | 28 | 5 | -3.36 | 1.35 | 7.15 | -18.00 | -7.75 | -4.00 | 2.50 | 14.00 |
|  | Change Week 24 | 28 | 5 | -2.96 | 1.57 | 8.32 | -18.00 | -8.00 | -4.00 | 1.00 | 27.00 |
| Placebo | Week 0 | 33 | 0 | 68.45 | 1.72 | 9.90 | 49.00 | 60.50 | 70.00 | 76.00 | 87.00 |
|  | Week 12 | 32 | 1 | 69.81 | 1.59 | 9.02 | 53.00 | 62.25 | 70.00 | 77.75 | 90.00 |
|  | Week 24 | 31 | 2 | 68.45 | 2.04 | 11.35 | 52.00 | 59.00 | 69.00 | 78.00 | 107.00 |
|  | Change Week 12 | 32 | 1 | 1.44 | 1.28 | 7.23 | -10.00 | -4.00 | 0.50 | 6.50 | 25.00 |
|  | Change Week 24 | 31 | 2 | 0.48 | 1.84 | 10.24 | -19.00 | -4.00 | -1.00 | 6.00 | 31.00 |

Table 14.7 Vital Signs – Summary Descriptive Statistics for Temperature by Product from Baseline (Week 0; Visit 2) to End of Intervention (Week 24; Visit 4) in the Safety Population (N=66)

| **Product Temperature** | | **N** | | **Mean** | **SEM** | **SD** | **Min** | **Quartiles** | | | |
| --- | --- | --- | --- | --- | --- | --- | --- | --- | --- | --- | --- |
|  |  | **Valid** | **Missing** |  |  |  |  | **Q1** | **Mdn** | **Q3** | **Max** |
| PT extract | Week 0 | 33 | 0 | 36.55 | 0.08 | 0.45 | 35.50 | 36.20 | 36.60 | 36.90 | 37.30 |
|  | Week 12 | 28 | 5 | 36.37 | 0.08 | 0.44 | 35.50 | 36.13 | 36.35 | 36.50 | 37.40 |
|  | Week 24 | 28 | 5 | 36.44 | 0.07 | 0.38 | 35.50 | 36.20 | 36.40 | 36.70 | 37.10 |
|  | Change Week 12 | 28 | 5 | -0.19 | 0.10 | 0.52 | -1.40 | -0.58 | -0.15 | 0.20 | 0.90 |
|  | Change Week 24 | 28 | 5 | -0.12 | 0.10 | 0.51 | -1.10 | -0.48 | -0.20 | 0.20 | 0.90 |
| Placebo | Week 0 | 33 | 0 | 36.35 | 0.07 | 0.38 | 35.50 | 36.10 | 36.20 | 36.70 | 37.00 |
|  | Week 12 | 32 | 1 | 36.38 | 0.07 | 0.37 | 35.60 | 36.20 | 36.30 | 36.50 | 37.30 |
|  | Week 24 | 31 | 2 | 36.43 | 0.05 | 0.29 | 35.90 | 36.20 | 36.50 | 36.60 | 37.10 |
|  | Change Week 12 | 32 | 1 | 0.03 | 0.09 | 0.51 | -0.90 | -0.40 | 0.10 | 0.40 | 1.10 |
|  | Change Week 24 | 31 | 2 | 0.10 | 0.07 | 0.39 | -0.90 | -0.10 | 0.20 | 0.40 | 0.80 |

Table 14.8 Safety Blood Parameters – Summary Descriptive Statistics for Blood electrolytes –Sodium by Product from Baseline (Week 0; Visit 2) to End of Intervention (Week 24; Visit 4) in the Safety Population (N=66)

| **Product Sodium [mmol/L]** | | **N** | | **Mean** | **SEM** | **SD** | **Min** | **Quartiles** | | | |
| --- | --- | --- | --- | --- | --- | --- | --- | --- | --- | --- | --- |
|  |  | **Valid** | **Missing** |  |  |  |  | **Q1** | **Mdn** | **Q3** | **Max** |
| PT extract | Week 0 | 33 | 0 | 139.52 | 0.33 | 1.91 | 135.00 | 138.50 | 140.00 | 141.00 | 143.00 |
|  | Week 12 | 28 | 5 | 140.36 | 0.26 | 1.37 | 138.00 | 139.00 | 140.00 | 142.00 | 143.00 |
|  | Week 24 | 28 | 5 | 140.14 | 0.37 | 1.96 | 136.00 | 139.00 | 140.00 | 142.00 | 144.00 |
|  | Change Week 12 | 28 | 5 | 0.46 | 0.27 | 1.45 | -2.00 | -0.75 | 1.00 | 1.00 | 3.00 |
|  | Change Week 24 | 28 | 5 | 0.25 | 0.35 | 1.88 | -3.00 | -1.00 | 0.00 | 2.00 | 4.00 |
| Placebo | Week 0 | 33 | 0 | 139.42 | 0.28 | 1.62 | 137.00 | 138.00 | 139.00 | 140.00 | 143.00 |
|  | Week 12 | 32 | 1 | 139.59 | 0.31 | 1.74 | 137.00 | 138.00 | 139.00 | 141.00 | 144.00 |
|  | Week 24 | 31 | 2 | 139.55 | 0.39 | 2.16 | 134.00 | 139.00 | 140.00 | 141.00 | 143.00 |
|  | Change Week 12 | 32 | 1 | 0.19 | 0.36 | 2.04 | -3.00 | -1.00 | 0.00 | 1.75 | 6.00 |
|  | Change Week 24 | 31 | 2 | 0.13 | 0.39 | 2.16 | -6.00 | -1.00 | 0.00 | 2.00 | 3.00 |

Table 14.10 Safety Blood Parameters – Summary Descriptive Statistics for Blood electrolytes – Potassium by Product from Baseline (Week 0; Visit 2) to End of Intervention (Week 24; Visit 4) in the Safety Population (N=66)

| **Product Potassium [mmol/L]** | | **N** | | **Mean** | **SEM** | **SD** | **Min** | **Quartiles** | | | |
| --- | --- | --- | --- | --- | --- | --- | --- | --- | --- | --- | --- |
|  |  | **Valid** | **Missing** |  |  |  |  | **Q1** | **Mdn** | **Q3** | **Max** |
| PT extract | Week 0 | 33 | 0 | 4.49 | 0.06 | 0.35 | 3.50 | 4.25 | 4.50 | 4.65 | 5.50 |
|  | Week 12 | 28 | 5 | 4.44 | 0.08 | 0.40 | 3.80 | 4.13 | 4.35 | 4.80 | 5.30 |
|  | Week 24 | 28 | 5 | 4.46 | 0.06 | 0.33 | 4.00 | 4.30 | 4.40 | 4.60 | 5.50 |
|  | Change Week 12 | 28 | 5 | -0.01 | 0.07 | 0.36 | -0.90 | -0.28 | -0.10 | 0.20 | 0.70 |
|  | Change Week 24 | 28 | 5 | 0.01 | 0.07 | 0.39 | -0.70 | -0.28 | -0.05 | 0.30 | 0.90 |
| Placebo | Week 0 | 33 | 0 | 4.52 | 0.06 | 0.35 | 4.00 | 4.25 | 4.50 | 4.75 | 5.30 |
|  | Week 12 | 32 | 1 | 4.58 | 0.07 | 0.38 | 4.00 | 4.30 | 4.50 | 4.70 | 5.50 |
|  | Week 24 | 31 | 2 | 4.49 | 0.05 | 0.27 | 4.00 | 4.30 | 4.50 | 4.70 | 5.00 |
|  | Change Week 12 | 32 | 1 | 0.06 | 0.06 | 0.32 | -0.60 | -0.18 | 0.00 | 0.28 | 0.80 |
|  | Change Week 24 | 31 | 2 | -0.04 | 0.06 | 0.34 | -0.80 | -0.30 | 0.00 | 0.20 | 1.00 |

Table 14.11 Safety Blood Parameters – Summary Descriptive Statistics for Blood electrolytes – Chloride by Product from Baseline (Week 0; Visit 2) to End of Intervention (Week 24; Visit 4) in the Safety Population (N=66)

| **Product Chloride [mmol/L]** | | **N** | | **Mean** | **SEM** | **SD** | **Min** | **Quartiles** | | | |
| --- | --- | --- | --- | --- | --- | --- | --- | --- | --- | --- | --- |
|  |  | **Valid** | **Missing** |  |  |  |  | **Q1** | **Mdn** | **Q3** | **Max** |
| PT extract | Week 0 | 33 | 0 | 104.18 | 0.34 | 1.96 | 101.00 | 102.50 | 104.00 | 106.00 | 107.00 |
|  | Week 12 | 28 | 5 | 104.86 | 0.27 | 1.41 | 102.00 | 104.00 | 105.00 | 106.00 | 107.00 |
|  | Week 24 | 28 | 5 | 104.64 | 0.33 | 1.77 | 102.00 | 103.00 | 104.50 | 106.00 | 108.00 |
|  | Change Week 12 | 28 | 5 | 0.39 | 0.36 | 1.93 | -3.00 | -1.00 | 0.50 | 2.00 | 4.00 |
|  | Change Week 24 | 28 | 5 | 0.18 | 0.41 | 2.16 | -3.00 | -1.00 | 0.00 | 2.00 | 4.00 |
| Placebo | Week 0 | 33 | 0 | 104.00 | 0.37 | 2.11 | 100.00 | 103.00 | 104.00 | 105.00 | 108.00 |
|  | Week 12 | 32 | 1 | 104.66 | 0.33 | 1.84 | 101.00 | 103.25 | 105.00 | 106.00 | 108.00 |
|  | Week 24 | 31 | 2 | 104.87 | 0.38 | 2.14 | 101.00 | 103.00 | 105.00 | 107.00 | 109.00 |
|  | Change Week 12 | 32 | 1 | 0.69 | 0.32 | 1.82 | -3.00 | -1.00 | 1.00 | 2.00 | 4.00 |
|  | Change Week 24 | 31 | 2 | 0.81 | 0.36 | 2.01 | -2.00 | -1.00 | 1.00 | 2.00 | 6.00 |

Table 14.12 Safety Blood Parameters – Summary Descriptive Statistics for Blood electrolytes – Calcium by Product from Baseline (Week 0; Visit 2) to End of Intervention (Week 24; Visit 4) in the Safety Population (N=66)

| **Product Calcium [mmol/L]** | | **N** | | **Mean** | **SEM** | **SD** | **Min** | **Quartiles** | | | |
| --- | --- | --- | --- | --- | --- | --- | --- | --- | --- | --- | --- |
|  |  | **Valid** | **Missing** |  |  |  |  | **Q1** | **Mdn** | **Q3** | **Max** |
| PT extract | Week 0 | 33 | 0 | 2.42 | 0.02 | 0.10 | 2.23 | 2.37 | 2.40 | 2.47 | 2.70 |
|  | Week 12 | 28 | 5 | 2.40 | 0.02 | 0.08 | 2.25 | 2.33 | 2.40 | 2.45 | 2.58 |
|  | Week 24 | 28 | 5 | 2.40 | 0.01 | 0.08 | 2.26 | 2.34 | 2.41 | 2.45 | 2.58 |
|  | Change Week 12 | 28 | 5 | -0.03 | 0.02 | 0.10 | -0.22 | -0.10 | -0.04 | 0.04 | 0.19 |
|  | Change Week 24 | 28 | 5 | -0.02 | 0.02 | 0.10 | -0.18 | -0.09 | -0.05 | 0.03 | 0.21 |
| Placebo | Week 0 | 33 | 0 | 2.42 | 0.01 | 0.08 | 2.29 | 2.36 | 2.42 | 2.46 | 2.60 |
|  | Week 12 | 32 | 1 | 2.38 | 0.02 | 0.10 | 2.18 | 2.30 | 2.38 | 2.46 | 2.55 |
|  | Week 24 | 31 | 2 | 2.39 | 0.02 | 0.09 | 2.25 | 2.32 | 2.39 | 2.44 | 2.56 |
|  | Change Week 12 | 32 | 1 | -0.05 | 0.02 | 0.09 | -0.27 | -0.10 | -0.06 | 0.02 | 0.12 |
|  | Change Week 24 | 31 | 2 | -0.03 | 0.01 | 0.08 | -0.26 | -0.07 | -0.02 | 0.01 | 0.10 |

Table 14.13 Safety Blood Parameters – Summary Descriptive Statistics for Blood electrolytes – Phosphate by Product from Baseline (Week 0; Visit 2) to End of Intervention (Week 24; Visit 4) in the Safety Population (N=66)

| **Product Phosphate [mmol/L]** | | **N** | | **Mean** | **SEM** | **SD** | **Min** | **Quartiles** | | | |
| --- | --- | --- | --- | --- | --- | --- | --- | --- | --- | --- | --- |
|  |  | **Valid** | **Missing** |  |  |  |  | **Q1** | **Mdn** | **Q3** | **Max** |
| PT extract | Week 0 | 33 | 0 | 1.14 | 0.03 | 0.18 | 0.59 | 1.09 | 1.16 | 1.26 | 1.35 |
|  | Week 12 | 28 | 5 | 1.09 | 0.03 | 0.16 | 0.77 | 1.01 | 1.10 | 1.17 | 1.39 |
|  | Week 24 | 28 | 5 | 1.15 | 0.03 | 0.15 | 0.88 | 1.03 | 1.15 | 1.28 | 1.39 |
|  | Change Week 12 | 28 | 5 | -0.03 | 0.03 | 0.15 | -0.28 | -0.16 | -0.04 | 0.06 | 0.30 |
|  | Change Week 24 | 28 | 5 | 0.03 | 0.03 | 0.17 | -0.27 | -0.07 | 0.02 | 0.14 | 0.39 |
| Placebo | Week 0 | 33 | 0 | 1.17 | 0.03 | 0.15 | 0.86 | 1.11 | 1.16 | 1.25 | 1.58 |
|  | Week 12 | 32 | 1 | 1.15 | 0.03 | 0.18 | 0.77 | 1.04 | 1.16 | 1.33 | 1.43 |
|  | Week 24 | 31 | 2 | 1.19 | 0.03 | 0.15 | 0.92 | 1.07 | 1.17 | 1.29 | 1.52 |
|  | Change Week 12 | 32 | 1 | -0.02 | 0.03 | 0.18 | -0.47 | -0.14 | -0.02 | 0.10 | 0.34 |
|  | Change Week 24 | 31 | 2 | 0.02 | 0.03 | 0.15 | -0.42 | -0.07 | 0.02 | 0.12 | 0.27 |

Table 14.14 Safety Blood Parameters – Summary Descriptive Statistics for Blood electrolytes – Magnesium by Product from Baseline (Week 0; Visit 2) to End of Intervention (Week 24; Visit 4) in the Safety Population (N=66)

| **Product Magnesium [mmol/L]** | | **N** | | **Mean** | **SEM** | **SD** | **Min** | **Quartiles** | | | |
| --- | --- | --- | --- | --- | --- | --- | --- | --- | --- | --- | --- |
|  |  | **Valid** | **Missing** |  |  |  |  | **Q1** | **Mdn** | **Q3** | **Max** |
| PT extract | Week 0 | 33 | 0 | 0.85 | 0.01 | 0.06 | 0.77 | 0.81 | 0.85 | 0.89 | 0.99 |
|  | Week 12 | 28 | 5 | 0.85 | 0.01 | 0.06 | 0.72 | 0.81 | 0.86 | 0.90 | 0.98 |
|  | Week 24 | 28 | 5 | 0.85 | 0.01 | 0.07 | 0.69 | 0.80 | 0.84 | 0.90 | 1.00 |
|  | Change Week 12 | 28 | 5 | 0.00 | 0.01 | 0.07 | -0.13 | -0.06 | 0.00 | 0.04 | 0.20 |
|  | Change Week 24 | 28 | 5 | 0.00 | 0.01 | 0.05 | -0.11 | -0.05 | -0.01 | 0.02 | 0.11 |
| Placebo | Week 0 | 33 | 0 | 0.84 | 0.01 | 0.06 | 0.68 | 0.80 | 0.83 | 0.87 | 0.97 |
|  | Week 12 | 32 | 1 | 0.85 | 0.01 | 0.06 | 0.68 | 0.80 | 0.85 | 0.89 | 0.93 |
|  | Week 24 | 31 | 2 | 0.86 | 0.02 | 0.09 | 0.63 | 0.82 | 0.85 | 0.89 | 1.17 |
|  | Change Week 12 | 32 | 1 | 0.01 | 0.01 | 0.05 | -0.09 | -0.03 | 0.02 | 0.04 | 0.10 |
|  | Change Week 24 | 31 | 2 | 0.02 | 0.01 | 0.06 | -0.09 | -0.01 | 0.02 | 0.04 | 0.24 |

Table 14.15 Safety Blood Parameters – Summary Descriptive Statistics for Renal Profile – Urea by Product from Baseline (Week 0; Visit 2) to End of Intervention (Week 24; Visit 4) in the Safety Population (N=66)

| **Product Urea [mmol/L]** | | **N** | | **Mean** | **SEM** | **SD** | **Min** | **Quartiles** | | | |
| --- | --- | --- | --- | --- | --- | --- | --- | --- | --- | --- | --- |
|  |  | **Valid** | **Missing** |  |  |  |  | **Q1** | **Mdn** | **Q3** | **Max** |
| PT extract | Week 0 | 33 | 0 | 5.33 | 0.18 | 1.06 | 2.90 | 4.50 | 5.50 | 6.00 | 7.80 |
|  | Week 12 | 28 | 5 | 5.62 | 0.25 | 1.35 | 3.90 | 4.90 | 5.35 | 5.90 | 9.80 |
|  | Week 24 | 28 | 5 | 6.05 | 0.26 | 1.38 | 3.70 | 4.65 | 5.95 | 7.30 | 9.40 |
|  | Change Week 12 | 28 | 5 | 0.23 | 0.29 | 1.53 | -2.60 | -0.60 | 0.05 | 0.88 | 5.30 |
|  | Change Week 24 | 28 | 5 | 0.65 | 0.25 | 1.30 | -1.50 | -0.10 | 0.50 | 1.28 | 4.90 |
| Placebo | Week 0 | 33 | 0 | 5.40 | 0.23 | 1.30 | 2.90 | 4.65 | 5.10 | 6.65 | 8.10 |
|  | Week 12 | 32 | 1 | 5.52 | 0.20 | 1.15 | 3.40 | 4.83 | 5.25 | 6.45 | 8.10 |
|  | Week 24 | 31 | 2 | 5.41 | 0.21 | 1.19 | 2.90 | 4.70 | 5.40 | 6.00 | 7.90 |
|  | Change Week 12 | 32 | 1 | 0.10 | 0.22 | 1.25 | -3.30 | -0.48 | 0.30 | 1.15 | 2.00 |
|  | Change Week 24 | 31 | 2 | -0.09 | 0.19 | 1.08 | -2.50 | -0.90 | -0.20 | 0.60 | 2.20 |

Table 14.16 Safety Blood Parameters – Summary Descriptive Statistics for Renal Profile – Creatinine by Product from Baseline (Week 0; Visit 2) to End of Intervention (Week 24; Visit 4) in the Safety Population (N=66)

| **Product Creatinine [umol/L]** | | **N** | | **Mean** | **SEM** | **SD** | **Min** | **Quartiles** | | | |
| --- | --- | --- | --- | --- | --- | --- | --- | --- | --- | --- | --- |
|  |  | **Valid** | **Missing** |  |  |  |  | **Q1** | **Mdn** | **Q3** | **Max** |
| PT extract | Week 0 | 33 | 0 | 72.55 | 2.00 | 11.47 | 60.00 | 64.00 | 67.00 | 81.00 | 104.00 |
|  | Week 12 | 28 | 5 | 71.57 | 1.96 | 10.39 | 56.00 | 64.00 | 72.00 | 77.50 | 104.00 |
|  | Week 24 | 28 | 5 | 73.68 | 2.07 | 10.96 | 58.00 | 65.00 | 71.50 | 78.75 | 102.00 |
|  | Change Week 12 | 28 | 5 | -1.04 | 1.25 | 6.60 | -22.00 | -2.00 | 0.00 | 2.00 | 13.00 |
|  | Change Week 24 | 28 | 5 | 1.07 | 0.98 | 5.17 | -10.00 | -2.00 | 2.00 | 4.50 | 15.00 |
| Placebo | Week 0 | 33 | 0 | 71.73 | 2.17 | 12.47 | 50.00 | 64.50 | 71.00 | 75.50 | 115.00 |
|  | Week 12 | 32 | 1 | 74.03 | 2.64 | 14.91 | 47.00 | 63.25 | 72.00 | 82.50 | 124.00 |
|  | Week 24 | 31 | 2 | 74.84 | 2.09 | 11.63 | 58.00 | 67.00 | 75.00 | 80.00 | 113.00 |
|  | Change Week 12 | 32 | 1 | 1.88 | 1.18 | 6.70 | -10.00 | -2.75 | 1.50 | 6.75 | 21.00 |
|  | Change Week 24 | 31 | 2 | 1.97 | 0.80 | 4.48 | -9.00 | -1.00 | 2.00 | 4.00 | 12.00 |

Table 14.17 Safety Blood Parameters – Summary Descriptive Statistics for Renal Profile – Uric Acid by Product from Baseline (Week 0; Visit 2) to End of Intervention (Week 24; Visit 4) in the Safety Population (N=66)

| **Product Uric Acid [umol/L]** | | **N** | | **Mean** | **SEM** | **SD** | **Min** | **Quartiles** | | | |
| --- | --- | --- | --- | --- | --- | --- | --- | --- | --- | --- | --- |
|  |  | **Valid** | **Missing** |  |  |  |  | **Q1** | **Mdn** | **Q3** | **Max** |
| PT extract | Week 0 | 33 | 0 | 321.18 | 13.73 | 78.84 | 168.00 | 266.00 | 325.00 | 372.00 | 461.00 |
|  | Week 12 | 28 | 5 | 310.75 | 13.05 | 69.03 | 195.00 | 260.25 | 287.00 | 360.00 | 451.00 |
|  | Week 24 | 28 | 5 | 326.75 | 13.65 | 72.22 | 209.00 | 266.00 | 331.50 | 394.00 | 443.00 |
|  | Change Week 12 | 28 | 5 | -20.14 | 9.31 | 49.25 | -136.00 | -48.25 | -15.50 | 23.25 | 50.00 |
|  | Change Week 24 | 28 | 5 | -4.14 | 9.36 | 49.51 | -102.00 | -46.00 | 7.00 | 30.25 | 69.00 |
| Placebo | Week 0 | 33 | 0 | 318.79 | 13.60 | 78.14 | 213.00 | 243.00 | 312.00 | 363.00 | 475.00 |
|  | Week 12 | 32 | 1 | 320.34 | 14.60 | 82.58 | 176.00 | 251.00 | 327.50 | 352.75 | 508.00 |
|  | Week 24 | 31 | 2 | 330.68 | 14.05 | 78.24 | 194.00 | 280.00 | 311.00 | 399.00 | 449.00 |
|  | Change Week 12 | 32 | 1 | -1.66 | 5.15 | 29.14 | -40.00 | -29.00 | -7.00 | 22.25 | 71.00 |
|  | Change Week 24 | 31 | 2 | 6.03 | 4.85 | 27.00 | -54.00 | -9.00 | 6.00 | 28.00 | 62.00 |

Table 14.18 Safety Blood Parameters – Summary Descriptive Statistics for Liver function tests–ALT by Product from Baseline (Week 0; Visit 2) to End of Intervention (Week 24; Visit 4) in the Safety Population (N=66)

| **Product Alanine Aminotransferase [IU/L]** | | **N** | | **Mean** | **SEM** | **SD** | **Min** | **Quartiles** | | | |
| --- | --- | --- | --- | --- | --- | --- | --- | --- | --- | --- | --- |
|  |  | **Valid** | **Missing** |  |  |  |  | **Q1** | **Mdn** | **Q3** | **Max** |
| PT extract | Week 0 | 33 | 0 | 21.12 | 1.59 | 9.14 | 9.00 | 14.50 | 19.00 | 28.00 | 47.00 |
|  | Week 12 | 28 | 5 | 21.11 | 2.03 | 10.73 | 8.00 | 13.25 | 17.00 | 26.50 | 45.00 |
|  | Week 24 | 28 | 5 | 20.29 | 1.92 | 10.14 | 9.00 | 13.00 | 17.00 | 24.75 | 48.00 |
|  | Change Week 12 | 28 | 5 | -0.93 | 1.39 | 7.38 | -24.00 | -4.75 | -0.50 | 3.00 | 13.00 |
|  | Change Week 24 | 28 | 5 | -1.75 | 1.61 | 8.51 | -26.00 | -5.75 | -2.00 | 1.75 | 16.00 |
| Placebo | Week 0 | 33 | 0 | 22.42 | 2.15 | 12.33 | 10.00 | 16.00 | 20.00 | 23.00 | 76.00 |
|  | Week 12 | 32 | 1 | 19.69 | 1.31 | 7.43 | 6.00 | 14.00 | 18.50 | 24.00 | 39.00 |
|  | Week 24 | 31 | 2 | 20.10 | 1.36 | 7.56 | 9.00 | 14.00 | 18.00 | 25.00 | 38.00 |
|  | Change Week 12 | 32 | 1 | -2.91 | 2.23 | 12.61 | -62.00 | -4.75 | -1.00 | 1.00 | 22.00 |
|  | Change Week 24 | 31 | 2 | -2.90 | 2.36 | 13.14 | -63.00 | -5.00 | -1.00 | 2.00 | 20.00 |

Table 14.19 Safety Blood Parameters – Summary Descriptive Statistics for Liver function tests–AST by Product from Baseline (Week 0; Visit 2) to End of Intervention (Week 24; Visit 4) in the Safety Population (N=66)

| **Product Aspartate Aminotransferase [IU/L]** | | **N** | | **Mean** | **SEM** | **SD** | **Min** | **Quartiles** | | | |
| --- | --- | --- | --- | --- | --- | --- | --- | --- | --- | --- | --- |
|  |  | **Valid** | **Missing** |  |  |  |  | **Q1** | **Mdn** | **Q3** | **Max** |
| PT extract | Week 0 | 33 | 0 | 21.27 | 1.02 | 5.86 | 15.00 | 18.00 | 20.00 | 23.00 | 41.00 |
|  | Week 12 | 28 | 5 | 22.68 | 1.39 | 7.33 | 14.00 | 17.00 | 21.00 | 26.75 | 48.00 |
|  | Week 24 | 28 | 5 | 21.61 | 1.03 | 5.47 | 15.00 | 17.00 | 20.50 | 25.00 | 34.00 |
|  | Change Week 12 | 28 | 5 | 1.25 | 1.33 | 7.04 | -18.00 | -2.75 | 1.00 | 4.75 | 22.00 |
|  | Change Week 24 | 28 | 5 | 0.18 | 1.05 | 5.54 | -19.00 | -2.75 | 1.00 | 3.00 | 10.00 |
| Placebo | Week 0 | 33 | 0 | 22.94 | 1.26 | 7.24 | 13.00 | 18.50 | 22.00 | 26.00 | 51.00 |
|  | Week 12 | 32 | 1 | 22.16 | 0.80 | 4.54 | 13.00 | 18.00 | 21.50 | 26.00 | 31.00 |
|  | Week 24 | 31 | 2 | 21.39 | 0.91 | 5.06 | 12.00 | 18.00 | 21.00 | 24.00 | 38.00 |
|  | Change Week 12 | 32 | 1 | -0.72 | 1.23 | 6.97 | -33.00 | -2.00 | 0.50 | 2.00 | 9.00 |
|  | Change Week 24 | 31 | 2 | -1.81 | 1.37 | 7.64 | -39.00 | -3.00 | -1.00 | 2.00 | 6.00 |

Table 14.20 Safety Blood Parameters – Summary Descriptive Statistics for Liver function tests–ALP by Product from Baseline (Week 0; Visit 2) to End of Intervention (Week 24; Visit 4) in the Safety Population (N=66)

| **Product Alkaline Phosphatase [IU/L]** | | **N** | | **Mean** | **SEM** | **SD** | **Min** | **Quartiles** | | | |
| --- | --- | --- | --- | --- | --- | --- | --- | --- | --- | --- | --- |
|  |  | **Valid** | **Missing** |  |  |  |  | **Q1** | **Mdn** | **Q3** | **Max** |
| PT extract | Week 0 | 33 | 0 | 82.97 | 3.75 | 21.54 | 51.00 | 62.00 | 83.00 | 99.50 | 131.00 |
|  | Week 12 | 28 | 5 | 82.00 | 4.07 | 21.55 | 51.00 | 64.50 | 79.50 | 101.25 | 132.00 |
|  | Week 24 | 28 | 5 | 75.43 | 3.67 | 19.40 | 52.00 | 58.50 | 71.50 | 86.25 | 119.00 |
|  | Change Week 12 | 28 | 5 | 0.32 | 1.77 | 9.36 | -20.00 | -4.00 | 0.00 | 4.00 | 21.00 |
|  | Change Week 24 | 28 | 5 | -6.25 | 1.84 | 9.75 | -29.00 | -12.75 | -5.00 | 0.50 | 15.00 |
| Placebo | Week 0 | 33 | 0 | 71.27 | 3.24 | 18.60 | 41.00 | 59.00 | 69.00 | 87.00 | 112.00 |
|  | Week 12 | 32 | 1 | 73.81 | 3.78 | 21.39 | 39.00 | 56.75 | 71.00 | 89.25 | 133.00 |
|  | Week 24 | 31 | 2 | 65.35 | 3.43 | 19.12 | 20.00 | 52.00 | 65.00 | 78.00 | 103.00 |
|  | Change Week 12 | 32 | 1 | 2.19 | 1.44 | 8.13 | -14.00 | -3.00 | 1.50 | 7.75 | 21.00 |
|  | Change Week 24 | 31 | 2 | -5.39 | 2.61 | 14.56 | -73.00 | -9.00 | -2.00 | 2.00 | 9.00 |

Table 14.21 Safety Blood Parameters – Summary Descriptive Statistics for Liver function tests–GGT by Product from Baseline (Week 0; Visit 2) to End of Intervention (Week 24; Visit 4) in the Safety Population (N=66)

| **Product Gamma‐Glutamyl Transferase [IU/L]** | | **N** | | **Mean** | **SEM** | **SD** | **Min** | **Quartiles** | | | |
| --- | --- | --- | --- | --- | --- | --- | --- | --- | --- | --- | --- |
|  |  | **Valid** | **Missing** |  |  |  |  | **Q1** | **Mdn** | **Q3** | **Max** |
| PT extract | Week 0 | 33 | 0 | 22.94 | 2.47 | 14.16 | 10.00 | 13.50 | 20.00 | 25.00 | 81.00 |
|  | Week 12 | 28 | 5 | 24.54 | 3.03 | 16.01 | 9.00 | 14.00 | 19.00 | 27.00 | 81.00 |
|  | Week 24 | 28 | 5 | 24.46 | 3.61 | 19.09 | 9.00 | 13.00 | 17.00 | 27.25 | 87.00 |
|  | Change Week 12 | 28 | 5 | 1.54 | 1.06 | 5.63 | -15.00 | 0.00 | 2.00 | 3.00 | 20.00 |
|  | Change Week 24 | 28 | 5 | 1.46 | 2.14 | 11.32 | -13.00 | -3.75 | -1.00 | 2.75 | 49.00 |
| Placebo | Week 0 | 33 | 0 | 23.97 | 1.99 | 11.44 | 12.00 | 15.50 | 22.00 | 29.00 | 68.00 |
|  | Week 12 | 32 | 1 | 25.13 | 2.01 | 11.39 | 11.00 | 16.25 | 22.00 | 34.00 | 58.00 |
|  | Week 24 | 31 | 2 | 24.90 | 2.32 | 12.92 | 11.00 | 15.00 | 20.00 | 37.00 | 67.00 |
|  | Change Week 12 | 32 | 1 | 0.81 | 1.60 | 9.03 | -31.00 | -2.00 | 0.00 | 3.00 | 23.00 |
|  | Change Week 24 | 31 | 2 | 0.81 | 1.79 | 9.95 | -28.00 | -2.00 | -1.00 | 2.00 | 36.00 |

Table 14.22 Safety Blood Parameters – Summary Descriptive Statistics for Liver function tests–TP by Product from Baseline (Week 0; Visit 2) to End of Intervention (Week 24; Visit 4) in the Safety Population (N=66)

| **Product Total Protein [g/L]** | | **N** | | **Mean** | **SEM** | **SD** | **Min** | **Quartiles** | | | |
| --- | --- | --- | --- | --- | --- | --- | --- | --- | --- | --- | --- |
|  |  | **Valid** | **Missing** |  |  |  |  | **Q1** | **Mdn** | **Q3** | **Max** |
| PT extract | Week 0 | 33 | 0 | 70.91 | 0.72 | 4.13 | 63.00 | 68.00 | 71.00 | 73.00 | 84.00 |
|  | Week 12 | 28 | 5 | 71.32 | 0.76 | 4.03 | 64.00 | 69.00 | 72.00 | 73.00 | 81.00 |
|  | Week 24 | 28 | 5 | 71.36 | 0.64 | 3.40 | 65.00 | 69.00 | 71.00 | 73.75 | 80.00 |
|  | Change Week 12 | 28 | 5 | 0.50 | 0.49 | 2.60 | -5.00 | -1.00 | 1.00 | 2.00 | 5.00 |
|  | Change Week 24 | 28 | 5 | 0.54 | 0.55 | 2.89 | -6.00 | -1.00 | 0.00 | 3.00 | 7.00 |
| Placebo | Week 0 | 33 | 0 | 70.52 | 0.63 | 3.62 | 64.00 | 68.00 | 71.00 | 73.00 | 80.00 |
|  | Week 12 | 32 | 1 | 70.31 | 0.68 | 3.82 | 63.00 | 67.25 | 71.00 | 72.00 | 79.00 |
|  | Week 24 | 31 | 2 | 70.87 | 0.62 | 3.47 | 64.00 | 68.00 | 71.00 | 73.00 | 79.00 |
|  | Change Week 12 | 32 | 1 | -0.28 | 0.54 | 3.04 | -7.00 | -2.00 | 0.00 | 1.00 | 8.00 |
|  | Change Week 24 | 31 | 2 | 0.06 | 0.48 | 2.68 | -6.00 | -1.00 | 0.00 | 2.00 | 4.00 |

Table 14.23 Safety Blood Parameters – Summary Descriptive Statistics for Liver function tests– Albumin by Product from Baseline (Week 0; Visit 2) to End of Intervention (Week 24; Visit 4) in the Safety Population (N=66)

| **Product Albumin [g/L]** | | **N** | | **Mean** | **SEM** | **SD** | **Min** | **Quartiles** | | | |
| --- | --- | --- | --- | --- | --- | --- | --- | --- | --- | --- | --- |
|  |  | **Valid** | **Missing** |  |  |  |  | **Q1** | **Mdn** | **Q3** | **Max** |
| PT extract | Week 0 | 33 | 0 | 43.45 | 0.40 | 2.28 | 39.00 | 42.00 | 44.00 | 45.00 | 49.00 |
|  | Week 12 | 28 | 5 | 44.00 | 0.40 | 2.13 | 40.00 | 42.00 | 45.00 | 46.00 | 47.00 |
|  | Week 24 | 28 | 5 | 44.29 | 0.47 | 2.46 | 39.00 | 42.25 | 45.00 | 46.00 | 50.00 |
|  | Change Week 12 | 28 | 5 | 0.36 | 0.33 | 1.75 | -3.00 | -1.00 | 0.00 | 1.00 | 4.00 |
|  | Change Week 24 | 28 | 5 | 0.64 | 0.35 | 1.85 | -2.00 | -1.00 | 1.00 | 2.00 | 4.00 |
| Placebo | Week 0 | 33 | 0 | 43.94 | 0.38 | 2.21 | 40.00 | 42.00 | 44.00 | 46.00 | 47.00 |
|  | Week 12 | 32 | 1 | 43.47 | 0.36 | 2.03 | 40.00 | 42.00 | 43.00 | 45.00 | 48.00 |
|  | Week 24 | 31 | 2 | 44.13 | 0.47 | 2.62 | 40.00 | 43.00 | 44.00 | 46.00 | 50.00 |
|  | Change Week 12 | 32 | 1 | -0.53 | 0.33 | 1.88 | -4.00 | -2.00 | 0.00 | 0.75 | 5.00 |
|  | Change Week 24 | 31 | 2 | 0.03 | 0.40 | 2.24 | -5.00 | -2.00 | 0.00 | 1.00 | 4.00 |

Table 14.24 Safety Blood Parameters – Summary Descriptive Statistics for Liver function tests– Globulin by Product from Baseline (Week 0; Visit 2) to End of Intervention (Week 24; Visit 4) in the Safety Population (N=66)

| **Product Globulins [g/L]** | | **N** | | **Mean** | **SEM** | **SD** | **Min** | **Quartiles** | | | |
| --- | --- | --- | --- | --- | --- | --- | --- | --- | --- | --- | --- |
|  |  | **Valid** | **Missing** |  |  |  |  | **Q1** | **Mdn** | **Q3** | **Max** |
| PT extract | Week 0 | 33 | 0 | 27.27 | 0.60 | 3.45 | 19.00 | 25.00 | 27.00 | 30.00 | 35.00 |
|  | Week 12 | 28 | 5 | 27.32 | 0.67 | 3.55 | 19.00 | 25.00 | 27.00 | 29.75 | 35.00 |
|  | Week 24 | 28 | 5 | 27.07 | 0.62 | 3.25 | 21.00 | 25.25 | 27.00 | 28.75 | 34.00 |
|  | Change Week 12 | 28 | 5 | 0.25 | 0.35 | 1.84 | -4.00 | -0.75 | 0.00 | 1.75 | 5.00 |
|  | Change Week 24 | 28 | 5 | 0.00 | 0.38 | 2.04 | -4.00 | -2.00 | 0.00 | 1.00 | 4.00 |
| Placebo | Week 0 | 33 | 0 | 26.58 | 0.46 | 2.65 | 22.00 | 24.50 | 26.00 | 28.00 | 33.00 |
|  | Week 12 | 32 | 1 | 26.84 | 0.54 | 3.05 | 22.00 | 24.00 | 26.00 | 29.75 | 32.00 |
|  | Week 24 | 31 | 2 | 26.74 | 0.43 | 2.37 | 23.00 | 25.00 | 27.00 | 28.00 | 31.00 |
|  | Change Week 12 | 32 | 1 | 0.25 | 0.41 | 2.30 | -7.00 | -1.00 | 0.00 | 2.00 | 4.00 |
|  | Change Week 24 | 31 | 2 | 0.03 | 0.29 | 1.62 | -3.00 | -1.00 | 0.00 | 2.00 | 3.00 |

Table 14.25 Safety Blood Parameters – Summary Descriptive Statistics for Liver function tests– Total Bilirubin by Product from Baseline (Week 0; Visit 2) to End of Intervention (Week 24; Visit 4) in the Safety Population (N=66)

| **Product Total Bilirubin [umol/L]** | | **N** | | **Mean** | **SEM** | **SD** | **Min** | **Quartiles** | | | |
| --- | --- | --- | --- | --- | --- | --- | --- | --- | --- | --- | --- |
|  |  | **Valid** | **Missing** |  |  |  |  | **Q1** | **Mdn** | **Q3** | **Max** |
| PT extract | Week 0 | 33 | 0 | 10.36 | 1.02 | 5.84 | 3.20 | 6.20 | 7.80 | 11.75 | 26.90 |
|  | Week 12 | 28 | 5 | 9.50 | 0.68 | 3.59 | 5.20 | 6.50 | 9.00 | 11.38 | 18.50 |
|  | Week 24 | 28 | 5 | 10.12 | 0.86 | 4.56 | 4.80 | 6.43 | 8.85 | 12.45 | 20.90 |
|  | Change Week 12 | 28 | 5 | -1.35 | 0.89 | 4.70 | -11.90 | -4.73 | -0.65 | 1.85 | 7.20 |
|  | Change Week 24 | 28 | 5 | -0.73 | 0.68 | 3.59 | -11.40 | -2.60 | -0.20 | 1.03 | 7.40 |
| Placebo | Week 0 | 33 | 0 | 9.42 | 0.50 | 2.88 | 5.60 | 6.65 | 9.80 | 11.10 | 15.90 |
|  | Week 12 | 32 | 1 | 9.24 | 0.68 | 3.86 | 4.20 | 6.93 | 8.45 | 11.10 | 24.30 |
|  | Week 24 | 31 | 2 | 9.40 | 0.65 | 3.63 | 4.20 | 6.90 | 8.70 | 10.70 | 22.20 |
|  | Change Week 12 | 32 | 1 | -0.15 | 0.48 | 2.69 | -5.20 | -2.38 | -0.15 | 1.48 | 8.40 |
|  | Change Week 24 | 31 | 2 | -0.10 | 0.46 | 2.58 | -4.20 | -2.00 | -0.30 | 1.40 | 6.30 |

Table 14.26 Safety Blood Parameters – Summary Descriptive Statistics for Differentials– White Cell Count by Product from Baseline (Week 0; Visit 2) to End of Intervention (Week 24; Visit 4) in the Safety Population (N=66)

| **Product White Cell Count [10e9/L]** | | **N** | | **Mean** | **SEM** | **SD** | **Min** | **Quartiles** | | | |
| --- | --- | --- | --- | --- | --- | --- | --- | --- | --- | --- | --- |
|  |  | **Valid** | **Missing** |  |  |  |  | **Q1** | **Mdn** | **Q3** | **Max** |
| PT extract | Week 0 | 33 | 0 | 6.41 | 0.28 | 1.61 | 2.84 | 5.22 | 6.38 | 7.28 | 10.12 |
|  | Week 12 | 28 | 5 | 6.36 | 0.26 | 1.38 | 3.27 | 5.61 | 6.39 | 7.48 | 8.86 |
|  | Week 24 | 28 | 5 | 6.29 | 0.26 | 1.36 | 3.81 | 5.34 | 6.13 | 7.26 | 9.35 |
|  | Change Week 12 | 28 | 5 | -0.02 | 0.20 | 1.03 | -2.17 | -0.76 | 0.15 | 0.69 | 1.77 |
|  | Change Week 24 | 28 | 5 | -0.09 | 0.22 | 1.18 | -2.67 | -1.23 | -0.11 | 0.95 | 1.68 |
| Placebo | Week 0 | 33 | 0 | 6.13 | 0.23 | 1.35 | 4.00 | 5.23 | 5.89 | 7.35 | 9.98 |
|  | Week 12 | 32 | 1 | 6.12 | 0.25 | 1.41 | 3.89 | 4.98 | 6.11 | 6.63 | 10.04 |
|  | Week 24 | 31 | 2 | 6.18 | 0.20 | 1.10 | 4.45 | 5.42 | 6.07 | 7.06 | 8.78 |
|  | Change Week 12 | 32 | 1 | -0.01 | 0.18 | 1.01 | -1.86 | -0.80 | -0.06 | 0.55 | 2.65 |
|  | Change Week 24 | 31 | 2 | 0.18 | 0.21 | 1.15 | -2.44 | -0.57 | 0.20 | 0.87 | 3.51 |

Table 14.27 Safety Blood Parameters – Summary Descriptive Statistics for Differentials– Red Cell Count by Product from Baseline (Week 0; Visit 2) to End of Intervention (Week 24; Visit 4) in the Safety Population (N=66)

| **Product Red Cell Count [10e12/L]** | | **N** | | **Mean** | **SEM** | **SD** | **Min** | **Quartiles** | | | |
| --- | --- | --- | --- | --- | --- | --- | --- | --- | --- | --- | --- |
|  |  | **Valid** | **Missing** |  |  |  |  | **Q1** | **Mdn** | **Q3** | **Max** |
| PT extract | Week 0 | 33 | 0 | 6.41 | 0.28 | 1.61 | 2.84 | 5.22 | 6.38 | 7.28 | 10.12 |
|  | Week 12 | 28 | 5 | 6.36 | 0.26 | 1.38 | 3.27 | 5.61 | 6.39 | 7.48 | 8.86 |
|  | Week 24 | 28 | 5 | 6.29 | 0.26 | 1.36 | 3.81 | 5.34 | 6.13 | 7.26 | 9.35 |
|  | Change Week 12 | 28 | 5 | -0.02 | 0.20 | 1.03 | -2.17 | -0.76 | 0.15 | 0.69 | 1.77 |
|  | Change Week 24 | 28 | 5 | -0.09 | 0.22 | 1.18 | -2.67 | -1.23 | -0.11 | 0.95 | 1.68 |
| Placebo | Week 0 | 33 | 0 | 6.13 | 0.23 | 1.35 | 4.00 | 5.23 | 5.89 | 7.35 | 9.98 |
|  | Week 12 | 32 | 1 | 6.12 | 0.25 | 1.41 | 3.89 | 4.98 | 6.11 | 6.63 | 10.04 |
|  | Week 24 | 31 | 2 | 6.18 | 0.20 | 1.10 | 4.45 | 5.42 | 6.07 | 7.06 | 8.78 |
|  | Change Week 12 | 32 | 1 | -0.01 | 0.18 | 1.01 | -1.86 | -0.80 | -0.06 | 0.55 | 2.65 |
|  | Change Week 24 | 31 | 2 | 0.18 | 0.21 | 1.15 | -2.44 | -0.57 | 0.20 | 0.87 | 3.51 |

Table 14.28 Safety Blood Parameters – Summary Descriptive Statistics for Differentials– Haematocrit by Product from Baseline (Week 0; Visit 2) to End of Intervention (Week 24; Visit 4) in the Safety Population (N=66)

| **Product Haematocrit [L/L]** | | **N** | | **Mean** | **SEM** | **SD** | **Min** | **Quartiles** | | | |
| --- | --- | --- | --- | --- | --- | --- | --- | --- | --- | --- | --- |
|  |  | **Valid** | **Missing** |  |  |  |  | **Q1** | **Mdn** | **Q3** | **Max** |
| PT extract | Week 0 | 33 | 0 | 0.43 | 0.01 | 0.03 | 0.36 | 0.41 | 0.43 | 0.45 | 0.49 |
|  | Week 12 | 28 | 5 | 0.43 | 0.01 | 0.03 | 0.37 | 0.41 | 0.43 | 0.45 | 0.49 |
|  | Week 24 | 28 | 5 | 0.44 | 0.01 | 0.03 | 0.38 | 0.42 | 0.44 | 0.47 | 0.50 |
|  | Change Week 12 | 28 | 5 | 0.00 | 0.00 | 0.02 | -0.03 | -0.01 | 0.00 | 0.01 | 0.03 |
|  | Change Week 24 | 28 | 5 | 0.01 | 0.00 | 0.02 | -0.02 | 0.00 | 0.01 | 0.02 | 0.04 |
| Placebo | Week 0 | 33 | 0 | 0.43 | 0.00 | 0.03 | 0.36 | 0.41 | 0.43 | 0.45 | 0.48 |
|  | Week 12 | 32 | 1 | 0.42 | 0.00 | 0.03 | 0.35 | 0.40 | 0.43 | 0.44 | 0.48 |
|  | Week 24 | 31 | 2 | 0.43 | 0.00 | 0.03 | 0.37 | 0.42 | 0.43 | 0.45 | 0.48 |
|  | Change Week 12 | 32 | 1 | -0.01 | 0.00 | 0.02 | -0.03 | -0.02 | -0.01 | 0.00 | 0.03 |
|  | Change Week 24 | 31 | 2 | 0.01 | 0.00 | 0.01 | -0.03 | 0.00 | 0.01 | 0.01 | 0.02 |

Table 14.29 Safety Blood Parameters – Summary Descriptive Statistics for Differentials– MCV by Product from Baseline (Week 0; Visit 2) to End of Intervention (Week 24; Visit 4) in the Safety Population (N=66)

| **Product Mean Corpuscular Volume [fL]** | | **N** | | **Mean** | **SEM** | **SD** | **Min** | **Quartiles** | | | |
| --- | --- | --- | --- | --- | --- | --- | --- | --- | --- | --- | --- |
|  |  | **Valid** | **Missing** |  |  |  |  | **Q1** | **Mdn** | **Q3** | **Max** |
| PT extract | Week 0 | 33 | 0 | 93.81 | 0.60 | 3.46 | 87.60 | 90.55 | 93.50 | 95.75 | 102.20 |
|  | Week 12 | 28 | 5 | 93.26 | 0.79 | 4.19 | 86.80 | 90.00 | 92.85 | 96.13 | 101.50 |
|  | Week 24 | 28 | 5 | 93.65 | 0.72 | 3.82 | 87.60 | 90.58 | 93.10 | 96.73 | 101.00 |
|  | Change Week 12 | 28 | 5 | -0.30 | 0.38 | 2.03 | -3.50 | -1.65 | -0.70 | 0.68 | 6.00 |
|  | Change Week 24 | 28 | 5 | 0.09 | 0.41 | 2.15 | -7.30 | -0.88 | 0.30 | 1.45 | 3.60 |
| Placebo | Week 0 | 33 | 0 | 94.15 | 0.62 | 3.57 | 85.40 | 91.35 | 94.70 | 96.60 | 101.20 |
|  | Week 12 | 32 | 1 | 93.86 | 0.70 | 3.93 | 84.10 | 91.53 | 94.40 | 96.83 | 101.90 |
|  | Week 24 | 31 | 2 | 94.41 | 0.70 | 3.90 | 84.50 | 91.00 | 95.50 | 97.60 | 101.70 |
|  | Change Week 12 | 32 | 1 | -0.31 | 0.26 | 1.47 | -3.20 | -1.38 | -0.20 | 0.48 | 4.50 |
|  | Change Week 24 | 31 | 2 | 0.23 | 0.24 | 1.33 | -2.40 | -0.50 | 0.30 | 1.50 | 2.50 |

Table 14.30 Safety Blood Parameters – Summary Descriptive Statistics for Differentials– Mean Corpuscular Haemoglobin (MCH) by Product from Baseline (Week 0; Visit 2) to End of Intervention (Week 24; Visit 4) in the Safety Population (N=66)

| **Product Mean Corpuscular Haemoglobin [pg]** | | **N** | | **Mean** | **SEM** | **SD** | **Min** | **Quartiles** | | | |
| --- | --- | --- | --- | --- | --- | --- | --- | --- | --- | --- | --- |
|  |  | **Valid** | **Missing** |  |  |  |  | **Q1** | **Mdn** | **Q3** | **Max** |
| PT extract | Week 0 | 33 | 0 | 30.56 | 0.25 | 1.41 | 28.20 | 29.40 | 30.20 | 31.60 | 33.90 |
|  | Week 12 | 28 | 5 | 30.52 | 0.32 | 1.68 | 28.00 | 29.10 | 30.45 | 31.65 | 34.10 |
|  | Week 24 | 28 | 5 | 30.13 | 0.24 | 1.29 | 28.10 | 29.33 | 29.85 | 31.28 | 32.60 |
|  | Change Week 12 | 28 | 5 | 0.01 | 0.12 | 0.61 | -1.20 | -0.30 | 0.00 | 0.30 | 1.60 |
|  | Change Week 24 | 28 | 5 | -0.39 | 0.13 | 0.69 | -1.50 | -0.98 | -0.35 | 0.10 | 1.20 |
| Placebo | Week 0 | 33 | 0 | 30.96 | 0.23 | 1.34 | 27.80 | 30.35 | 31.00 | 32.20 | 32.90 |
|  | Week 12 | 32 | 1 | 30.91 | 0.24 | 1.34 | 28.00 | 29.95 | 31.05 | 31.90 | 33.10 |
|  | Week 24 | 31 | 2 | 30.59 | 0.27 | 1.53 | 26.90 | 29.30 | 30.90 | 31.60 | 32.90 |
|  | Change Week 12 | 32 | 1 | -0.07 | 0.10 | 0.55 | -0.90 | -0.48 | -0.10 | 0.20 | 1.40 |
|  | Change Week 24 | 31 | 2 | -0.40 | 0.12 | 0.67 | -1.70 | -0.80 | -0.60 | 0.00 | 1.20 |

Table 16

Table 14.31 Safety Blood Parameters – Summary Descriptive Statistics for Differentials– Mean Corpuscular Haemoglobin Concentration (MCHC) by Product from Baseline (Week 0; Visit 2) to End of Intervention (Week 24; Visit 4) in the Safety Population (N=66)

| **Product MCHC [g/dL]** | | **N** | | **Mean** | **SEM** | **SD** | **Min** | **Quartiles** | | | |
| --- | --- | --- | --- | --- | --- | --- | --- | --- | --- | --- | --- |
|  |  | **Valid** | **Missing** |  |  |  |  | **Q1** | **Mdn** | **Q3** | **Max** |
| PT extract | Week 0 | 33 | 0 | 32.57 | 0.14 | 0.79 | 30.70 | 32.05 | 32.60 | 33.10 | 34.60 |
|  | Week 12 | 28 | 5 | 32.72 | 0.15 | 0.81 | 31.30 | 32.20 | 32.65 | 33.18 | 34.50 |
|  | Week 24 | 28 | 5 | 32.18 | 0.16 | 0.87 | 30.30 | 31.75 | 32.25 | 32.68 | 33.90 |
|  | Change Week 12 | 28 | 5 | 0.12 | 0.14 | 0.72 | -1.80 | -0.38 | 0.10 | 0.75 | 1.20 |
|  | Change Week 24 | 28 | 5 | -0.43 | 0.15 | 0.82 | -2.20 | -1.00 | -0.45 | 0.15 | 1.30 |
| Placebo | Week 0 | 33 | 0 | 32.88 | 0.12 | 0.72 | 31.60 | 32.30 | 32.80 | 33.40 | 34.20 |
|  | Week 12 | 32 | 1 | 32.95 | 0.16 | 0.92 | 31.40 | 32.23 | 32.80 | 33.75 | 34.50 |
|  | Week 24 | 31 | 2 | 32.42 | 0.18 | 1.00 | 31.00 | 31.60 | 32.20 | 33.20 | 34.60 |
|  | Change Week 12 | 32 | 1 | 0.06 | 0.11 | 0.63 | -1.10 | -0.40 | -0.10 | 0.50 | 1.60 |
|  | Change Week 24 | 31 | 2 | -0.48 | 0.15 | 0.81 | -2.00 | -1.10 | -0.70 | 0.10 | 1.20 |

Table 14.32 Safety Blood Parameters – Summary Descriptive Statistics for Differentials– Red Cell Distribution Width (RDW) by Product from Baseline (Week 0; Visit 2) to End of Intervention (Week 24; Visit 4) in the Safety Population (N=66)

| **Product RDW [%]** | | **N** | | **Mean** | **SEM** | **SD** | **Min** | **Quartiles** | | | |
| --- | --- | --- | --- | --- | --- | --- | --- | --- | --- | --- | --- |
|  |  | **Valid** | **Missing** |  |  |  |  | **Q1** | **Mdn** | **Q3** | **Max** |
| PT extract | Week 0 | 33 | 0 | 12.96 | 0.14 | 0.80 | 11.80 | 12.40 | 13.00 | 13.30 | 14.90 |
|  | Week 12 | 28 | 5 | 12.95 | 0.12 | 0.64 | 12.00 | 12.53 | 12.70 | 13.30 | 14.60 |
|  | Week 24 | 28 | 5 | 12.92 | 0.15 | 0.78 | 11.90 | 12.40 | 12.75 | 13.20 | 14.80 |
|  | Change Week 12 | 28 | 5 | -0.05 | 0.09 | 0.48 | -1.20 | -0.38 | -0.10 | 0.30 | 0.80 |
|  | Change Week 24 | 28 | 5 | -0.08 | 0.08 | 0.40 | -1.00 | -0.30 | -0.10 | 0.18 | 0.60 |
| Placebo | Week 0 | 33 | 0 | 13.02 | 0.10 | 0.57 | 12.00 | 12.65 | 12.80 | 13.35 | 14.20 |
|  | Week 12 | 32 | 1 | 12.97 | 0.12 | 0.66 | 11.90 | 12.48 | 12.90 | 13.38 | 14.90 |
|  | Week 24 | 31 | 2 | 13.03 | 0.12 | 0.70 | 12.00 | 12.50 | 13.00 | 13.40 | 14.50 |
|  | Change Week 12 | 32 | 1 | -0.06 | 0.06 | 0.34 | -0.70 | -0.30 | -0.10 | 0.18 | 0.70 |
|  | Change Week 24 | 31 | 2 | 0.00 | 0.07 | 0.38 | -0.60 | -0.30 | 0.00 | 0.20 | 0.70 |

Table 14.33 Safety Blood Parameters – Summary Descriptive Statistics for Differentials– Platelets by Product from Baseline (Week 0; Visit 2) to End of Intervention (Week 24; Visit 4) in the Safety Population (N=66)

| **Product Platelets [10e9/L]** | | **N** | | **Mean** | **SEM** | **SD** | **Min** | **Quartiles** | | | |
| --- | --- | --- | --- | --- | --- | --- | --- | --- | --- | --- | --- |
|  |  | **Valid** | **Missing** |  |  |  |  | **Q1** | **Mdn** | **Q3** | **Max** |
| PT extract | Week 0 | 33 | 0 | 278.42 | 8.28 | 47.55 | 187.00 | 246.00 | 279.00 | 307.50 | 370.00 |
|  | Week 12 | 28 | 5 | 268.75 | 9.24 | 48.88 | 167.00 | 230.50 | 269.00 | 307.50 | 370.00 |
|  | Week 24 | 28 | 5 | 269.96 | 8.94 | 47.31 | 168.00 | 244.50 | 270.00 | 303.50 | 373.00 |
|  | Change Week 12 | 28 | 5 | -7.68 | 5.94 | 31.43 | -94.00 | -20.00 | -4.00 | 10.50 | 59.00 |
|  | Change Week 24 | 28 | 5 | -6.46 | 5.41 | 28.63 | -101.00 | -26.75 | 1.50 | 17.25 | 32.00 |
| Placebo | Week 0 | 33 | 0 | 280.39 | 13.61 | 78.21 | 134.00 | 228.50 | 265.00 | 316.50 | 507.00 |
|  | Week 12 | 32 | 1 | 278.13 | 12.72 | 71.93 | 135.00 | 236.25 | 261.50 | 309.00 | 443.00 |
|  | Week 24 | 31 | 2 | 281.06 | 12.14 | 67.56 | 150.00 | 238.00 | 265.00 | 330.00 | 426.00 |
|  | Change Week 12 | 32 | 1 | -4.00 | 4.41 | 24.92 | -83.00 | -21.00 | -1.50 | 16.50 | 35.00 |
|  | Change Week 24 | 31 | 2 | -0.19 | 4.92 | 27.39 | -81.00 | -18.00 | 3.00 | 16.00 | 53.00 |

Table 14.34 Safety Blood Parameters – Summary Descriptive Statistics for Differentials– Neutrophils by Product from Baseline (Week 0; Visit 2) to End of Intervention (Week 24; Visit 4) in the Safety Population (N=66)

| **Product Neutrophils [10e9/L]** | | **N** | | **Mean** | **SEM** | **SD** | **Min** | **Quartiles** | | | |
| --- | --- | --- | --- | --- | --- | --- | --- | --- | --- | --- | --- |
|  |  | **Valid** | **Missing** |  |  |  |  | **Q1** | **Mdn** | **Q3** | **Max** |
| PT extract | Week 0 | 33 | 0 | 3.93 | 0.22 | 1.26 | 1.48 | 3.05 | 3.78 | 4.65 | 7.76 |
|  | Week 12 | 28 | 5 | 3.83 | 0.20 | 1.07 | 1.62 | 3.29 | 3.68 | 4.52 | 6.40 |
|  | Week 24 | 28 | 5 | 3.74 | 0.19 | 1.01 | 1.94 | 3.14 | 3.67 | 4.27 | 6.16 |
|  | Change Week 12 | 28 | 5 | -0.04 | 0.19 | 1.00 | -2.25 | -0.61 | 0.09 | 0.69 | 1.80 |
|  | Change Week 24 | 28 | 5 | -0.13 | 0.19 | 1.00 | -2.39 | -0.56 | -0.05 | 0.69 | 1.22 |
| Placebo | Week 0 | 33 | 0 | 3.56 | 0.18 | 1.05 | 1.77 | 2.97 | 3.46 | 4.16 | 6.68 |
|  | Week 12 | 32 | 1 | 3.54 | 0.18 | 1.00 | 1.71 | 2.95 | 3.36 | 4.25 | 6.51 |
|  | Week 24 | 31 | 2 | 3.56 | 0.19 | 1.06 | 2.19 | 2.82 | 3.22 | 4.11 | 6.45 |
|  | Change Week 12 | 32 | 1 | -0.02 | 0.13 | 0.73 | -1.70 | -0.50 | -0.03 | 0.51 | 1.75 |
|  | Change Week 24 | 31 | 2 | 0.10 | 0.19 | 1.08 | -2.42 | -0.60 | 0.01 | 0.66 | 3.74 |

Table 14.35 Safety Blood Parameters – Summary Descriptive Statistics for Differentials– Lymphocytes by Product from Baseline (Week 0; Visit 2) to End of Intervention (Week 24; Visit 4) in the Safety Population (N=66)

| **Product Lymphocytes [10e9/L]** | | **N** | | **Mean** | **SEM** | **SD** | **Min** | **Quartiles** | | | |
| --- | --- | --- | --- | --- | --- | --- | --- | --- | --- | --- | --- |
|  |  | **Valid** | **Missing** |  |  |  |  | **Q1** | **Mdn** | **Q3** | **Max** |
| PT extract | Week 0 | 33 | 0 | 1.74 | 0.08 | 0.47 | 0.78 | 1.33 | 1.86 | 1.94 | 2.97 |
|  | Week 12 | 28 | 5 | 1.78 | 0.09 | 0.47 | 0.51 | 1.43 | 1.83 | 1.98 | 2.77 |
|  | Week 24 | 28 | 5 | 1.79 | 0.06 | 0.33 | 1.30 | 1.49 | 1.75 | 2.10 | 2.29 |
|  | Change Week 12 | 28 | 5 | 0.00 | 0.07 | 0.35 | -1.51 | -0.13 | 0.05 | 0.15 | 0.40 |
|  | Change Week 24 | 28 | 5 | 0.01 | 0.05 | 0.27 | -0.73 | -0.14 | 0.06 | 0.20 | 0.35 |
| Placebo | Week 0 | 33 | 0 | 1.80 | 0.09 | 0.51 | 0.97 | 1.43 | 1.76 | 2.16 | 2.81 |
|  | Week 12 | 32 | 1 | 1.74 | 0.08 | 0.48 | 0.90 | 1.42 | 1.69 | 2.10 | 2.76 |
|  | Week 24 | 31 | 2 | 1.80 | 0.09 | 0.48 | 0.97 | 1.48 | 1.73 | 2.16 | 2.85 |
|  | Change Week 12 | 32 | 1 | -0.05 | 0.06 | 0.35 | -0.99 | -0.22 | -0.05 | 0.14 | 0.85 |
|  | Change Week 24 | 31 | 2 | 0.03 | 0.05 | 0.29 | -0.90 | -0.03 | 0.04 | 0.21 | 0.62 |

Table 14.36 Safety Blood Parameters – Summary Descriptive Statistics for Differentials– Monocytes by Product from Baseline (Week 0; Visit 2) to End of Intervention (Week 24; Visit 4) in the Safety Population (N=66)

| **Product Monocytes [10e9/L]** | | **N** | | **Mean** | **SEM** | **SD** | **Min** | **Quartiles** | | | |
| --- | --- | --- | --- | --- | --- | --- | --- | --- | --- | --- | --- |
|  |  | **Valid** | **Missing** |  |  |  |  | **Q1** | **Mdn** | **Q3** | **Max** |
| PT extract | Week 0 | 33 | 0 | 0.54 | 0.03 | 0.17 | 0.32 | 0.40 | 0.48 | 0.68 | 0.90 |
|  | Week 12 | 28 | 5 | 0.54 | 0.03 | 0.15 | 0.31 | 0.43 | 0.51 | 0.63 | 0.91 |
|  | Week 24 | 28 | 5 | 0.54 | 0.03 | 0.17 | 0.31 | 0.43 | 0.51 | 0.61 | 1.06 |
|  | Change Week 12 | 28 | 5 | 0.01 | 0.02 | 0.09 | -0.21 | -0.06 | 0.04 | 0.08 | 0.17 |
|  | Change Week 24 | 28 | 5 | 0.02 | 0.02 | 0.12 | -0.25 | -0.04 | 0.03 | 0.08 | 0.44 |
| Placebo | Week 0 | 33 | 0 | 0.58 | 0.05 | 0.31 | 0.35 | 0.45 | 0.51 | 0.57 | 2.17 |
|  | Week 12 | 32 | 1 | 0.62 | 0.11 | 0.62 | 0.32 | 0.43 | 0.50 | 0.59 | 3.98 |
|  | Week 24 | 31 | 2 | 0.61 | 0.07 | 0.38 | 0.36 | 0.45 | 0.50 | 0.64 | 2.51 |
|  | Change Week 12 | 32 | 1 | 0.04 | 0.06 | 0.34 | -0.29 | -0.07 | -0.02 | 0.08 | 1.81 |
|  | Change Week 24 | 31 | 2 | 0.02 | 0.02 | 0.10 | -0.17 | -0.05 | 0.02 | 0.09 | 0.34 |

Table 14.37 Safety Blood Parameters – Summary Descriptive Statistics for Differentials– Eosinophils by Product from Baseline (Week 0; Visit 2) to End of Intervention (Week 24; Visit 4) in the Safety Population (N=66)

| **Product Eosinophils [10e9/L]** | | **N** | | **Mean** | **SEM** | **SD** | **Min** | **Quartiles** | | | |
| --- | --- | --- | --- | --- | --- | --- | --- | --- | --- | --- | --- |
|  |  | **Valid** | **Missing** |  |  |  |  | **Q1** | **Mdn** | **Q3** | **Max** |
| PT extract | Week 0 | 33 | 0 | 0.15 | 0.01 | 0.08 | 0.00 | 0.08 | 0.14 | 0.22 | 0.29 |
|  | Week 12 | 28 | 5 | 0.17 | 0.02 | 0.09 | 0.00 | 0.10 | 0.17 | 0.23 | 0.35 |
|  | Week 24 | 28 | 5 | 0.17 | 0.02 | 0.11 | 0.00 | 0.09 | 0.16 | 0.22 | 0.47 |
|  | Change Week 12 | 28 | 5 | 0.01 | 0.01 | 0.06 | -0.08 | -0.03 | 0.02 | 0.05 | 0.12 |
|  | Change Week 24 | 28 | 5 | 0.01 | 0.01 | 0.07 | -0.11 | -0.03 | 0.00 | 0.05 | 0.24 |
| Placebo | Week 0 | 33 | 0 | 0.15 | 0.02 | 0.10 | 0.03 | 0.09 | 0.13 | 0.17 | 0.53 |
|  | Week 12 | 32 | 1 | 0.16 | 0.02 | 0.10 | 0.04 | 0.09 | 0.14 | 0.20 | 0.48 |
|  | Week 24 | 31 | 2 | 0.16 | 0.02 | 0.12 | 0.04 | 0.09 | 0.14 | 0.17 | 0.55 |
|  | Change Week 12 | 32 | 1 | 0.01 | 0.01 | 0.05 | -0.07 | -0.03 | 0.01 | 0.03 | 0.19 |
|  | Change Week 24 | 31 | 2 | 0.02 | 0.01 | 0.05 | -0.11 | -0.01 | 0.01 | 0.03 | 0.20 |

Table 14.38 Safety Blood Parameters – Summary Descriptive Statistics for Differentials– Basophils by Product from Baseline (Week 0; Visit 2) to End of Intervention (Week 24; Visit 4) in the Safety Population (N=66)

| **Product Basophils [10e9/L]** | | **N** | | **Mean** | **SEM** | **SD** | **Min** | **Quartiles** | | | |
| --- | --- | --- | --- | --- | --- | --- | --- | --- | --- | --- | --- |
|  |  | **Valid** | **Missing** |  |  |  |  | **Q1** | **Mdn** | **Q3** | **Max** |
| PT extract | Week 0 | 33 | 0 | 0.05 | 0.00 | 0.02 | 0.01 | 0.03 | 0.04 | 0.06 | 0.10 |
|  | Week 12 | 28 | 5 | 0.05 | 0.00 | 0.02 | 0.01 | 0.03 | 0.04 | 0.05 | 0.11 |
|  | Week 24 | 28 | 5 | 0.05 | 0.00 | 0.02 | 0.01 | 0.03 | 0.04 | 0.06 | 0.10 |
|  | Change Week 12 | 28 | 5 | 0.00 | 0.00 | 0.01 | -0.02 | -0.01 | 0.00 | 0.01 | 0.04 |
|  | Change Week 24 | 28 | 5 | 0.00 | 0.00 | 0.01 | -0.03 | -0.01 | 0.00 | 0.01 | 0.04 |
| Placebo | Week 0 | 33 | 0 | 0.04 | 0.00 | 0.02 | 0.01 | 0.03 | 0.04 | 0.06 | 0.09 |
|  | Week 12 | 32 | 1 | 0.05 | 0.00 | 0.02 | 0.02 | 0.03 | 0.05 | 0.07 | 0.12 |
|  | Week 24 | 31 | 2 | 0.05 | 0.00 | 0.02 | 0.01 | 0.03 | 0.05 | 0.06 | 0.10 |
|  | Change Week 12 | 32 | 1 | 0.00 | 0.00 | 0.02 | -0.02 | -0.01 | 0.01 | 0.02 | 0.04 |
|  | Change Week 24 | 31 | 2 | 0.00 | 0.00 | 0.01 | -0.04 | -0.01 | 0.00 | 0.01 | 0.03 |

**AE Listing for The Eight Participants With AE associated to Protocol Deviations (and removed from ITT analysis)**

| **SubjectID** | **Product** | **PT (SOC)** | **Start Date** | **End Date** | **IP start date** | **Outcome** | **Causality** | **Severity** |
| --- | --- | --- | --- | --- | --- | --- | --- | --- |
| 10001 | Pt extract | Dizziness (Cardiac disorders) | 06/Oct/2022 | 26/Oct/2022 | 04/Oct/2022 | Recovered | Definitely related | Mild |
| 10002 | Placebo | Tooth infection (Infections and infestations( | 04/Nov/2022 | 30/Nov/2022 | 27/Sep/2022 | Recovered | Not related | Moderate |
| 10003 | Pt extract | Constipation (Gastrointestinal disorders) | 29/Sep/2022 | 19/Oct/2022 | 28/Sep/2022 | Recovered | Definitely related | Moderate |
| 10004 | Pt extract | Diarrhoea (Gastrointestinal disorders) | 09/Oct/2022 | 12/Oct/2022 | 06/Oct/2022 | Recovered | Possibly related | Moderate |
| 10004 | Pt extract | Nausea (Gastrointestinal disorders) | 09/Oct/2022 |  | 06/Oct/2022 | Ongoing | Possibly related | Mild |
| 10005 | Pt extract | Monocyte count increased (Investigations) | 25/Nov/2022 |  | 11/Oct/2022 | Ongoing | Unlikely related | Mild |
| 10006 | Placebo | Lower respiratory tract infection (Respiratory, thoracic and mediastinal disorders) | 08/Mar/2023 | 11/Apr/2023 | 04/Nov/2022 | Increase in Severity | Not related | Moderate |
| 10007 | Placebo | Lower respiratory tract infection (Respiratory, thoracic and mediastinal disorders) | 24/Feb/2023 | 05/Mar/2023 | 07/Nov/2022 | Recovered | Not related | Moderate |
| 10008 | Pt extract | Abdominal discomfort (Gastrointestinal disorders) | 25/Nov/2022 |  | 28/Nov/2022 | Ongoing | Not related | Mild |
